# Transient distortions of the South Atlantic Anomaly radiation environments driven by electric fields

**DOI:** 10.1038/s41467-026-74984-z

**Published:** 2026-07-03

**Authors:** Ze-Fan Yin, Yi-Xin Sun, Xu-Zhi Zhou, Qiu-Gang Zong, Ying Liu, Ze-Jun Hu, Yoshiharu Omura, Robert Rankin, Hong Zou, Yu-Guang Ye

**Affiliations:** 1 School of Earth and Space Sciences, Peking University, Beijing, China; 2 State Key Laboratory of Lunar and Planetary Sciences, Macau University of Science and Technology, Macau, China; 3 Center for Space Physics and Astronomy, Polar Research Institute of China, Shanghai, China; 4 Research Institute for Sustainable Humanosphere, Kyoto University, Kyoto, Japan; 5 Department of Physics, University of Alberta, Edmonton, AB Canada

**Keywords:** Magnetospheric physics, Natural hazards

## Abstract

Energetic electrons in Earth’s inner radiation belt pose significant hazards to spacecraft systems, with the strongest radiation in low-Earth orbit (LEO) mostly confined to the South Atlantic Anomaly (SAA) region. Once considered stable, the inner belt is now understood to exhibit significant variability. Using data from the low-Earth-orbit Macau Science Satellite-1 mission, we report transient distortions of the SAA radiation environments, observationally characterized by enhanced fluxes of energetic electrons outside the traditional SAA radiation region, appearing either attached to or detached from its boundary. We show that these distortions can be explained by large-scale electric-field perturbations that adiabatically alter the electron mirror heights, which can be further modulated by ultra-low-frequency waves. Test-particle simulations successfully reproduce the observational features and provide crucial constraints on properties of the associated electric fields. These findings reveal a distinct manifestation of inner-belt variability, extending the electron radiation risks beyond the expected boundaries of the SAA radiation environments.

## Abstract (web summary)

The South Atlantic Anomaly presents a radiation hazard to low-Earth-orbit satellites. Here, the authors use Macau Science Satellite-1 data to show that large-scale electric fields can transiently extend this hazard beyond its nominal boundaries.

## Introduction

**Fig. 1:**
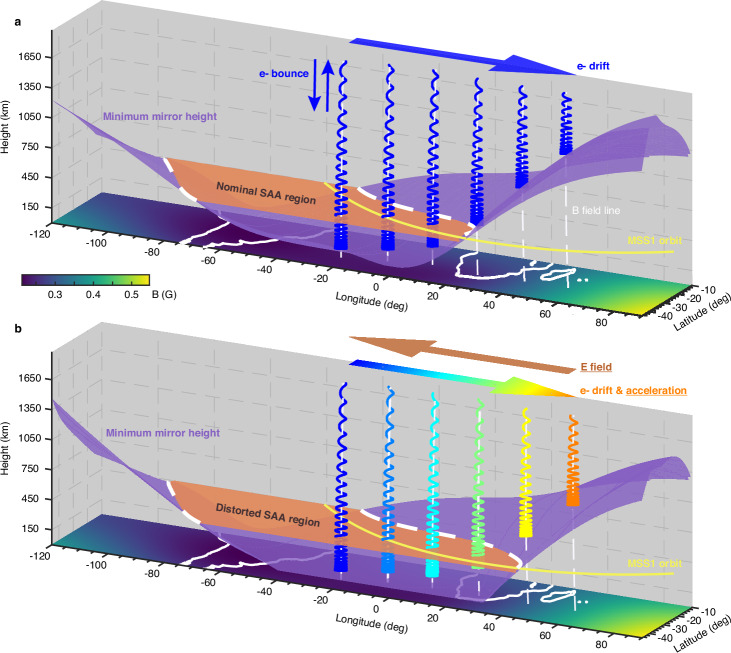
Schematic illustration of the transient distortion of the SAA radiation environments driven by electric-field perturbations. **a** The unperturbed state. The purple surface represents the lower boundary of the region with stably-trapped electrons, defined by the mirror points of electrons that graze the 100-km atmospheric precipitation boundary at their lowest altitudes along their respective drift-bounce shells. Electrons mirroring above this surface are stably trapped, whereas those mirroring below are within the drift loss cone. The MSS1 orbit (yellow line) is shown at an altitude of 450 km. The SAA radiation environments observed at the satellite altitude correspond to the area where the purple surface falls below the satellite orbital plane (orange plane), with its boundary defined by the intersection of the two surfaces (white dashed lines). **b** The perturbed state. A westward-directed electric field accelerates the eastward-drifting electrons (represented by the colored helical lines), adiabatically lowering their mirror points. This process enables the purple surface to dive further below the satellite altitude in the eastern sector, manifested by an eastward extension of the SAA radiation region in satellite observations (the extended orange plane).

The Van Allen radiation belts consist of charged particles ranging from tens of keV to hundreds of MeV trapped by Earth’s geomagnetic field^1–3^, posing significant hazards to spacecraft and astronauts in near-Earth space^4^. The electron radiation belts feature an inner (with geocentric distances between approximately 1.2 and 2 Earth radii (*R*_*E*_)) and an outer zone (beyond about 3*R*_*E*_), separated by a slot region of reduced fluxes^5,6^. Compared with the highly-variable outer zone, the inner belt is generally considered more stable, with particle dynamics primarily governed by the geomagnetic field configuration. A prominent feature of the inner belt is the South Atlantic Anomaly (SAA), where the magnetic field strength is significantly weaker than that of an ideal Earth-centered dipole. This depression allows the stably-trapped electrons in the inner belt to descend towards altitudes of a few hundred kilometers (yet still above the 100-km atmospheric precipitation boundary, see illustrations in Fig. 1a)^7,8^. For spacecraft observations in low-Earth orbit (LEO), the SAA radiation region can thus be defined as the area where these electrons are accessible to the spacecraft, with its boundary given by the intersection of the minimum mirror-altitudes for stably-trapped electrons (purple surface in Fig. 1a) with the spacecraft orbital plane (orange plane in Fig. 1a). Thus, trapped electrons can be expected inside, but not outside, the SAA radiation region, posing severe risks to LEO spacecraft^9,10^. While the relativistic population (>1 MeV) has long been associated with deep dielectric charging, electrons at lower energies (tens to hundreds of keV) still pose critical risks. They are the primary drivers of surface charging and of internal charging in thin spacecraft materials such as thermal blanket layers^11^, and they contribute significantly to the cumulative total ionizing dose (TID) degradation^12^ and to the interference for sensitive scientific payloads like X-ray and gamma-ray detectors^13,14^.

Although more stable than the outer zone, the inner belt has increasingly been recognized as susceptible to dynamic influences from natural and anthropogenic sources, including lightning-induced electron precipitation^15–17^, very-low-frequency transmitter emissions^18–20^, and large-scale electric-field perturbations^21,22^. The latter have been proposed to originate from the penetration of high-latitude electric fields toward the equator^23–26^. Westward-directed electric fields, in particular, can drive energetic electrons radially inward, contributing to the flux enhancements of forbidden energetic electrons (FEE) below approximately 1.2 *R*_*E*_^27,28^. The electric-field perturbations can also induce inhomogeneous particle distributions, producing drift-periodic peaks and valleys in particle spectrograms known as zebra stripes^29^. The drift-frequency separation between successive zebra stripes encodes temporal information about the structure’s formation, which can help infer the approximate onset time of electric-field perturbations^21,30,31^.

While numerous observations support the existence of inner-belt electric-field perturbations, their properties remain poorly constrained due to the difficulty of direct measurements. Indirect inferences from observational signatures also face significant limitations. For example, onset-time estimates derived from zebra-stripe observations are typically associated with uncertainties on the order of hours. They provide even less insight into the field duration or amplitude, since both short-lived and long-lasting perturbations can produce nearly identical stripe patterns^32,33^. Moreover, while FEE enhancements suggest electric-field strengths of several mV/m^27^, models for zebra-stripe formation often yield electric fields of less than 1 mV/m^32,34^. This discrepancy highlights the substantial uncertainties in our understanding of transient electric fields in near-Earth space.

Here, we show that such perturbations also distort the spatio-temporal distributions of energetic electrons in the SAA. Specifically, we examine the immediate response of the SAA radiation environments to these perturbations using electron observations from the Macau Science Satellite-1 (MSS1) mission, which operates in LEO at an inclination of 41^∘^ and an altitude of approximately 450–500 km (see Methods subsection “Macau Science Satellite-1” for details). The transient distortions appear as longitudinal extensions of the energetic electron distributions. They manifest as either abrupt, short-duration flux enhancements of energetic electrons outside the nominal SAA boundary (the radiation boundary in the unperturbed state), forming vertical stripes in energy spectrograms, or seamless expansions of the SAA radiation region. We demonstrate that these features are driven by large-scale electric-field perturbations, which adiabatically modulate the mirror points of inner-belt electrons, effectively distorting the electron distributions at LEO. Unlike zebra stripes, which have long served as indirect evidence of electric-field perturbations yielding weak constraints on field amplitude and duration, the transient distortions of the SAA radiation environments offer sharper constraints on properties of these electric-field perturbations, thereby advancing our understanding of transient space weather dynamics.

## Results

### Event I: Observations of the vertical stripe

**Fig. 2:**
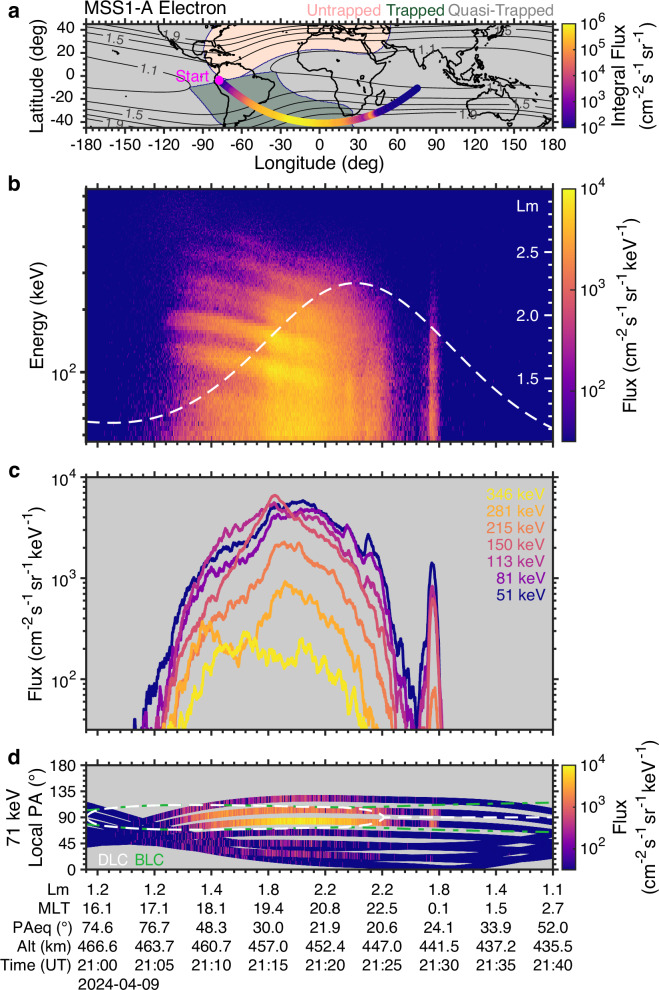
Overview of MSS1-A observations for the vertical stripe event on April 09, 2024 (Event I). **a** Satellite ground track, with the color representing the energy-integral fluxes of electrons with local pitch angle around 90^∘^. The background colormap indicates different trapping regions at the satellite’s orbital altitude, accompanied by McIlwain L-parameter contours with an interval of 0.2. **b** Energy-time spectrogram for electrons near 90^∘^ pitch angle, with the dashed white line representing L-shell variations. **c** Temporal variations of electron fluxes for several energy channels in (**b**). **d** Local pitch-angle (PA) distributions for 71-keV electrons, measured by nine detectors. The white dashed and green dash-dotted lines represent the drift loss cone (DLC) and bounce loss cone (BLC), respectively. The ephemeris data listed at the bottom include the McIlwain L-parameter (Lm), magnetic local time (MLT), the equatorial pitch angle (PAeq) of a locally-mirroring electron, and the satellite geodetic altitude (Alt). Source data are provided as a Source data file.

Figure 2 presents MSS1-A observations on April 9, 2024 (hereafter referred to as Event I). When MSS1-A traverses the SAA (see Fig. 2a for the satellite ground track), significant flux enhancements of energetic electrons are observed (see Fig. 2b, c for energy- and time-dependent fluxes of electrons with local pitch angles around 90^∘^; pitch angle denotes the angle between particle velocity and magnetic field). Most of these electrons are concentrated at pitch angles near 90^∘^ (Fig. 2d), corresponding to stably-trapped electrons outside the drift loss cone (DLC, white dashed curve). At pitch angles farther from 90^∘^, the fluxes drop sharply, consistent with theoretical expectations: electrons within the bounce loss cone (BLC, green dash-dotted curve) precipitate within a bounce period, while quasi-trapped electrons (with pitch angles between the BLC and DLC) mirror locally but cannot complete a full drift cycle before precipitation as they drift towards locations with weaker field. The electron energy spectrogram also exhibits distinct zebra stripes (Fig. 2b), likely signatures of electric-field disturbances several hours earlier.

After crossing the nominal SAA boundary around 21:25 UT, where the DLC expands to include electrons at 90^∘^ pitch angle, MSS1-A observes the expected sharp reduction in electron fluxes (Fig. 2b–d). A few minutes later, however, MSS1-A detects a sudden, orders-of-magnitude flux enhancement of electrons within the energy range of approximately 40–200 keV (see observations around 21:30 UT in Fig. 2b–d). The flux enhancement persists for about 2 min with minimal energy dispersion, forming a vertical stripe in the energy spectrogram (Fig. 2b). The pitch-angle coverage of these enhanced fluxes is predominantly around 90^∘^, inside the DLC but outside the BLC (between the white and green curves). Both the flux levels and angular distributions of the electrons within the vertical stripe are similar to those within the nominal SAA region, as if the SAA radiation environments are transiently distorted to encompass a wider area.

Note that during this time interval, MSS1-A experiences a slight decrease in geodetic altitude (see ephemeris labels in Fig. 2). Such a gradual altitude variation cannot account for the sudden flux enhancements, as the satellite stays outside the nominal SAA boundary since around 21:25 UT (the DLC remains 90^∘^ in Fig. 2d), indicating that the vertical stripe represents a distortion of electron distributions rather than an orbital artifact. Also, the vertical stripe duration (on the order of minutes) far exceeds the time scale of lightning-induced electron precipitation (typically <1 s)^15^, which rules out lightning-induced whistler waves as the cause.

We propose that the vertical stripe is an immediate response of the inner-belt electrons to large-scale electric-field perturbations. In the presence of westward-directed electric fields, eastward-drifting electrons in the inner belt would be accelerated while conserving the first and second adiabatic invariants^2^. Based on the conservation of the first adiabatic invariant, *μ* = *P*^2^/(2*m*_0_*B*_*m*_), where *P* is the electron momentum, *m*_0_ is the electron rest mass, and *B*_*m*_ is the mirror-point magnetic strength, an increase in energy (momentum) corresponds to a stronger *B*_*m*_, which shifts the mirror point to lower altitudes^35^. Consequently, when stably-trapped electrons drift beyond the nominal SAA boundary and encounter a westward-directed electric field, they would be accelerated to reach a mirror altitude lower than the unperturbed levels (see illustrations of particle trajectories in Fig. 1b). This process explains the electron flux enhancements at satellite altitude, which is below the mirror points of stably-trapped electrons should there be no electric-field perturbations (compare the green trajectory in Fig. 1b and its counterpart in Fig. 1a).

To examine this hypothesis, we note that the same electric field responsible for vertical-stripe generation would also lead to the gradual formation of zebra stripes in the forthcoming hours. Such structures are indeed observed (see energy spectrograms from both MSS1-A and the other MSS1 satellite, MSS1-B, during consecutive orbits in Supplementary Figs. 1 and 2). In the next subsection, we analyze the detailed observations, especially those from MSS1-B 7 h later, when zebra-stripe patterns are fully developed, to further support our proposed mechanism.

### Event I: Follow-up observations for zebra stripes

**Fig. 3:**
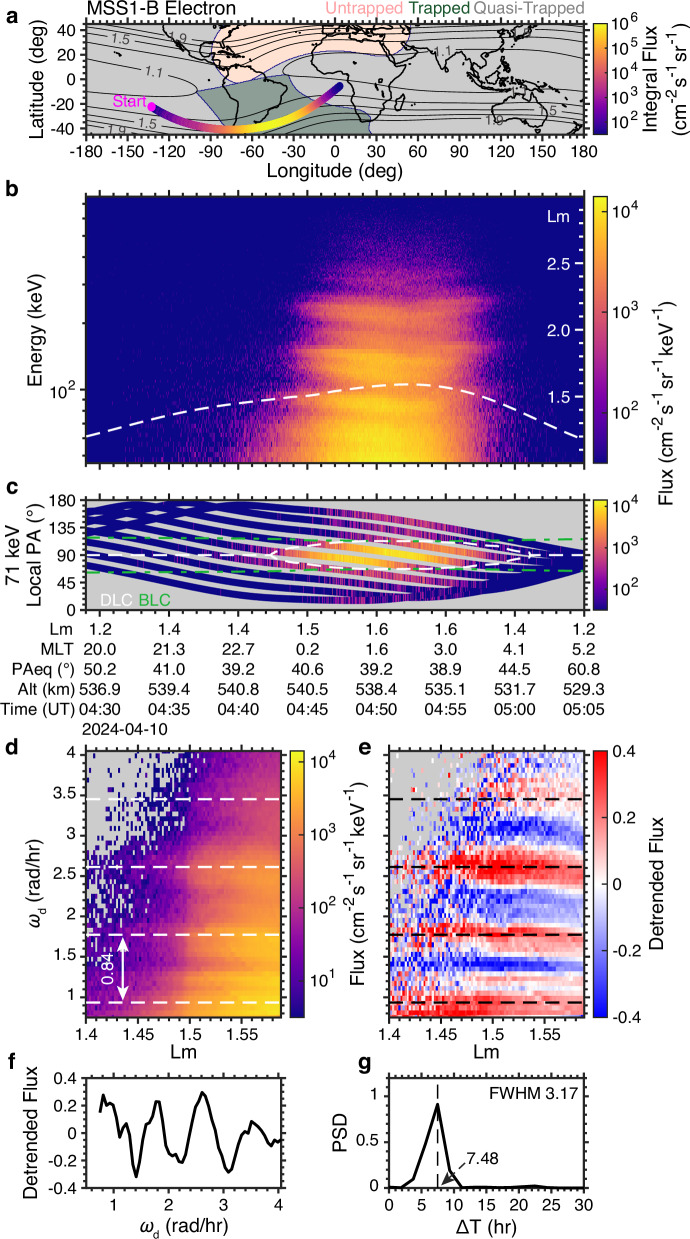
Overview of MSS1-B observations for the zebra stripes on April 10, 2024. **a** Satellite ground track, color-coded by the energy-integral fluxes of electrons with local pitch angle around 90^∘^. The background colormap indicates different trapping regions at the satellite’s orbital altitude, accompanied by McIlwain L-parameter contours with an interval of 0.2. **b** Energy-time spectrogram for electrons near 90^∘^ pitch angles, with the dashed white line representing L-shell variations. **c** Local pitch-angle (PA) distributions for 71-keV electrons. **d**,** e** show the observed electron fluxes and the detrended values as a function of particle drift frequency (*ω*_d_) and L-shell. **f**,** g** represent the L-shell-averaged detrended flux values and the corresponding power spectral density (PSD; in arbitrary units) from Fourier analysis. The full-width half-maximum (FWHM) is also indicated. Source data are provided as a Source data file.

Seven hours after the appearance of the vertical stripe, MSS1-B traverses the SAA and observes enhanced fluxes of energetic electrons (Fig. 3a–c). The energy-time spectrogram for electrons with pitch angles around 90^∘^ exhibits clear zebra stripes (Fig. 3b), which can be rearranged as functions of L-shell and electron drift frequency (Fig. 3d; The L-shell parameter represents, in *R*_*E*_, the geocentric distance where a magnetic field line crosses the magnetic equator; see Methods subsection “Zebra stripe analysis”), to display a series of nearly-horizontal stripes. The detrended logarithmic fluxes, calculated as $\ln (J)-\overline{\ln (J)}$ where *J* represents the electron flux and $\overline{\ln (J)}$ denotes the moving average of ln(J), are shown in Fig. 3e illustrating that these stripes are evenly spaced in drift frequency. Figure 3f displays the variation of detrended fluxes averaged over L-shells, followed by its Fourier spectrum in Fig. 3g, confirming the periodicity of zebra stripes as a function of drift frequency. The separation between adjacent stripes is approximately 0.84 rad/h (see horizontal lines in Fig. 3d, e), according to Liu et al.^30^, corresponds to an evolution time of about 7.5 h since the electric-field perturbation onset (Fig. 3g).

In other words, the estimated onset time of the perturbation falls between 21:11 UT and 21:31 UT on April 9, immediately preceding the vertical-stripe observations by MSS1-A (Fig. 2). Similar conclusions can also be made based on the analysis of consecutive zebra-stripe observations in Supplementary Figs. 1 and 2. We should note, however, that the full-width half-maximum (FWHM) of the Fourier power distribution in Fig. 3g, together with those in Supplementary Figs. 1 and 2, indicates an uncertainty of hours in the onset-time determination. Despite this uncertainty, the timing alignment supports our scenario that the vertical stripe results from large-scale electric-field perturbations. In the following subsection, we employ backward-tracing simulations^36^ of test electrons launched from the spacecraft location to reproduce the observational characteristics in Figs. 2 and 3, which reveal the causal relationship of vertical stripes with the perturbed electric field and place important constraints on the perturbation properties.

### Event I: Simulations

The particle-tracing simulations require a geomagnetic field model in the inner belt, which is naturally the International Geomagnetic Reference Field (IGRF)^37^. However, we are obliged by the significant computational cost of electron tracing in the full IGRF field to employ an eccentric tilted dipole model that provides a good approximation to IGRF, especially near the SAA region (see Methods subsection “Simulation setup”). Test electrons are then traced backward in time from the satellite positions within the eccentric-tilted solar magnetic (SM) coordinate system, with a large-scale dawn-to-dusk electric field applied from 21:21 to 21:29 UT (see Methods subsection “Electric field perturbations” for details).

**Fig. 4:**
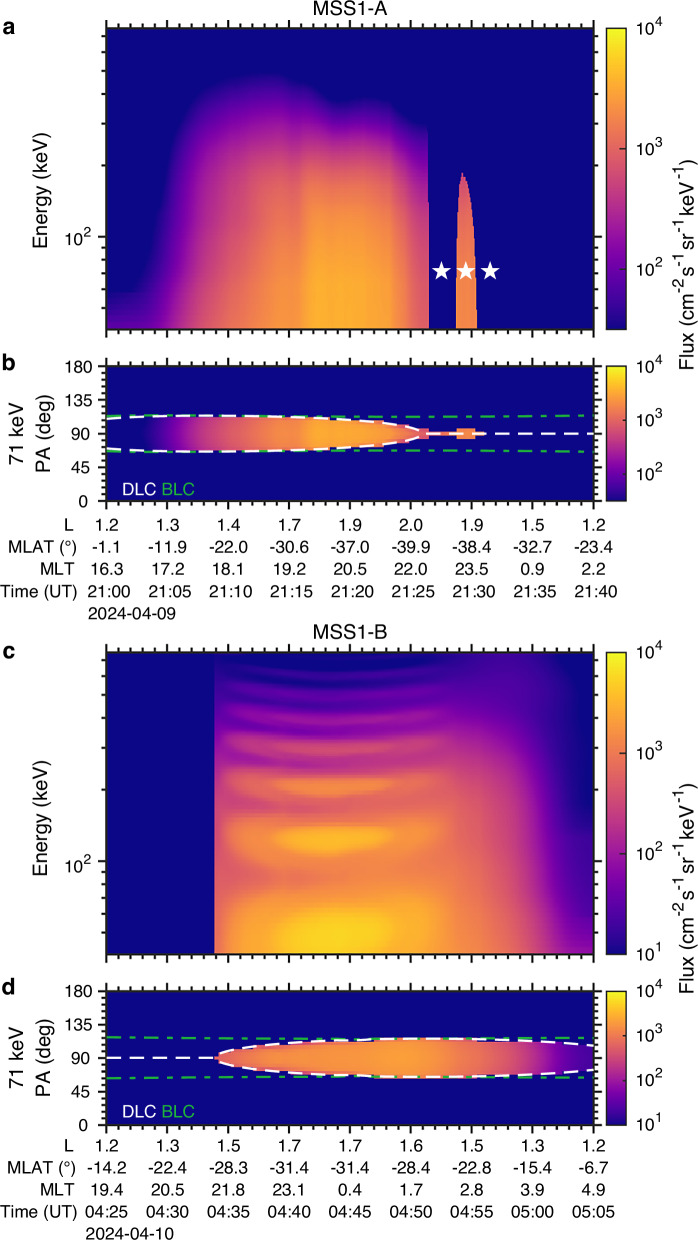
Simulation results for Event I. **a** Simulated energy spectrogram of electron fluxes with local pitch angle 85^∘^ and **b** local pitch-angle (PA) distributions for 71-keV electrons along the MSS1-A trajectory. The star symbols denote three representative test electrons selected for detailed trajectory analysis below. The white dashed and green dash-dotted lines represent the drift loss cone (DLC) and bounce loss cone (BLC), respectively. **c** Simulated energy spectrograms of locally mirroring electrons and **d** local pitch-angle distributions for 71-keV electrons along the MSS1-B trajectory several hours later. The ephemeris data include the L-shell (L), magnetic latitude (MLAT), and magnetic local time (MLT). Source data are provided as a Source data file.

The simulated energy spectrogram for electrons with an 85^∘^ local pitch angle at MSS1-A positions is shown in Fig. 4a, and the pitch-angle distributions for 71-keV electrons are given in Fig. 4b. The simulation reproduces the abrupt appearance of the vertical stripe, characterized by nearly energy-dispersionless, short-duration flux enhancements around local 90^∘^ pitch angle, in good agreement with MSS1-A observations in Fig. 2. Figure 4c, d show the simulation results at MSS1-B locations several hours afterwards, which reproduce the observations of zebra stripes for locally-mirroring electrons and confined pitch-angle distributions outside the local DLC (compared to MSS1-B observations in Fig. 3).

**Fig. 5:**
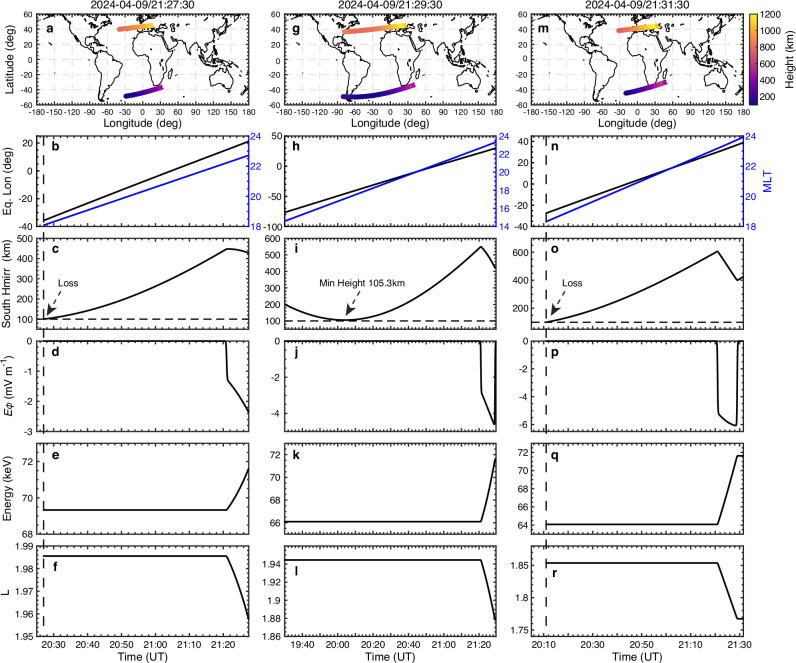
Variations of electrons in the test-particle simulation for Event I. The left column shows the results for electrons encountering the satellite before the vertical stripe appearance, including **a** the geographical locations of mirror points in the northern and southern hemispheres with color representing the mirror heights, and the temporal variations of **b** the geographical longitude at magnetic equator (Eq. Lon; black) and magnetic local time (MLT; blue), **c** the mirror height (Hmirr) in southern hemisphere, **d** the encountered azimuthal electric field *E*_*ϕ*_, **e** the energy change, and **f** the L-shell. The middle column **g-l** and the right column **m-r** are in the same format as the left column, except that they display the simulation results for electrons observed within and after the vertical stripe, respectively. Source data are provided as a Source data file.

To further understand the vertical-stripe generation, we examine the trajectories of representative test electrons encountering MSS1-A before, during, and after the vertical-stripe appearance, as shown in Fig. 5. The observation times and energies of these electrons are marked as star symbols in Fig. 4a. For the electron within the vertical stripe (middle column), the mirror height minimizes during its eastward drift across the SAA at about 105.3 km (Fig. 5g–i), which is higher than the characteristic precipitation boundary at 100 km, indicating that the electron is stably trapped outside the DLC should there be no electric-field perturbations. After that, the mirror height gradually increases to maintain the same mirror-point magnetic strength as the electron drifts eastward, until it encounters the westward-directed electric field (Fig. 5j) in the midnight sector around 21:21 UT. This electric field leads to the acceleration (Fig. 5k), radially-inward motion (Fig. 5l), and more importantly, mirror-height decrease (Fig. 5i) of the electron under the conservation of the first and second adiabatic invariants. It is the reduction in mirror height that allows the satellite at approximately 450 km to observe these originally trapped electrons, whose unperturbed mirror altitudes would be otherwise much higher than the satellite orbit.

For the test electron encountering the satellite before the vertical stripe (left column in Fig. 5), it may also be accelerated by the westward-directed electric field. However, weaker electric fields and shorter interaction duration (Fig. 5d) yield a smaller energy gain (Fig. 5e) and, consequently, a smaller mirror-height reduction (Fig. 5c). The backward-tracing trajectory shows that this electron originates from the precipitation boundary at 100 km. In other words, if an electron could reach the satellite under such a moderate mirror-height decline, its previous mirror height in the SAA region would have been less than 100 km, which is subject to atmospheric precipitation. As a result, the electron energy spectrogram exhibits a period with very low flux levels (set to be zero in simulations) before the vertical stripe (Fig. 4a).

On the other hand, the electron encountering the satellite after the vertical stripe experiences the full electric pulse, resulting in a greater energy enhancement (Fig. 5q) and a more pronounced decrease in mirror height (Fig. 5o). However, because the satellite moves eastward at a near-constant altitude while the mirror heights of stably-trapped electrons increase dramatically as they drift eastward away from the SAA, they require even stronger energy enhancements and larger mirror-height reductions to reach the satellite’s altitude. In other words, as the satellite continues moving eastward, it will eventually reach a longitude where no stably-trapped electrons could descend to the spacecraft’s altitude, given the finite amplitude and duration of the pulsed electric field. In the simulation, this is also manifested by the electron originating from the precipitation boundary (Fig. 5o), which contributes to the very low fluxes after the vertical stripe (Fig. 4a).

Therefore, the finite duration of the vertical stripe and its isolation from the nominal SAA region arise from the combined influence of the large-scale geomagnetic field configurations and the spatio-temporal extents of the electric-field perturbations. In case the electric field exhibits different characteristics (e.g., an earlier onset allowing longer interaction), it is possible that the electrons before the vertical-stripe appearance (left column in Fig. 5) could be traced back to the stably-trapped region rather than the precipitation boundary. The flux enhancements, in turn, would connect seamlessly to the nominal SAA region, forming a contiguous eastward expansion. Such an event is presented in the next subsection.

### Event II: Contiguous expansion of the SAA radiation region

**Fig. 6:**
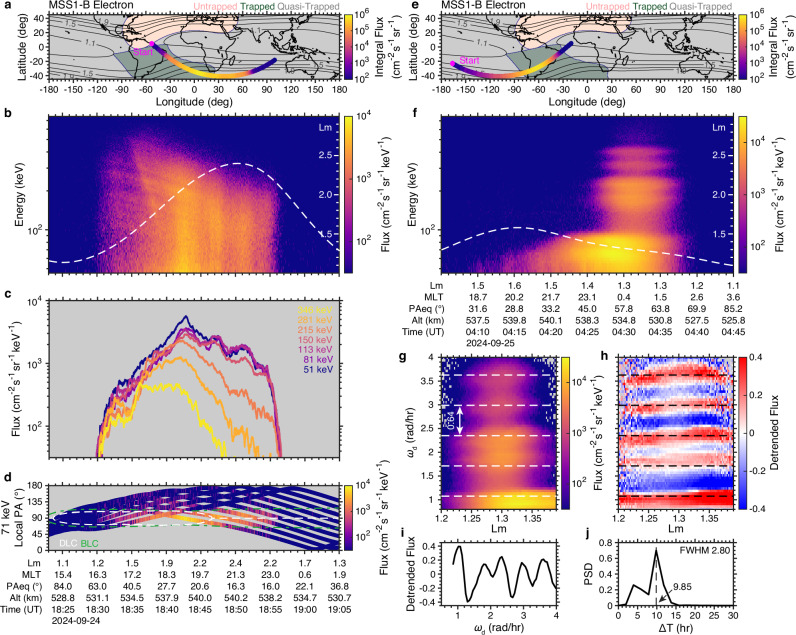
Overview of spacecraft observations for Event II, characterized by a contiguous expansion of the SAA electron environments. The left column displays the observations from MSS1-B on September 24, 2024. **a** Satellite ground track, with the color representing the energy-integral fluxes of electrons with local pitch angle around 90^∘^. The background colormap indicates different trapping regions at the satellite’s orbital altitude, accompanied by McIlwain L-parameter contours with an interval of 0.2. **b** Energy-time spectrogram for electrons near 90^∘^ pitch angle, with the dashed white line representing L-shell variations. **c** Temporal variations of electron fluxes for several energy channels. **d** Local pitch-angle (PA) distributions for 71-keV electrons. The white dashed and green dash-dotted lines represent the drift loss cone (DLC) and bounce loss cone (BLC), respectively. The right panels exhibit its observations for the zebra stripes hours later on September 25, 2024, including **e** satellite ground track and **f** energy-time spectrograms, which are in the same format as (**a**, **b**), **g, h** electron fluxes and detrended values as a function of particle drift frequency (*ω*_d_) and L-shell, and **i**,** j** the L-shell-averaged detrended flux values and the corresponding power spectral density (PSD; in arbitrary units) from Fourier analysis, with the full-width half-maximum (FWHM) labeled. Source data are provided as a Source data file.

Figure 6 shows the MSS1-B observations on September 24, 2024 (Event II). Similar to the case in Event I, the spacecraft traverses the SAA and observes the flux enhancements of stably-trapped electrons (Fig. 6a–d). As MSS1-B approaches the eastern edge of the nominal SAA region, the local DLC gradually increases to 90^∘^, marking its theoretical exit around 18:47 UT (Fig. 6d). However, unlike the sharp flux reduction typically observed at the SAA boundary, the electron energy spectrograms and pitch-angle distributions exhibit no significant variation. The enhanced electron fluxes outside the nominal SAA boundary (where the local DLC is 90^∘^), similar to Event I, are interpreted as the result of electric-field perturbations. Unlike the isolated structure in Event I, the enhanced fluxes here are connected seamlessly to the nominal SAA region, appearing as an overall eastward expansion of the radiation environments.

Also, the presence of large-scale electric-field perturbations during this event is supported by the observations of zebra stripes several hours afterwards, when MSS1-B traverses the western part of the SAA (Fig. 6e, f). Applying the same analysis as in the previous event, Fig. 6g, h display the observed electron fluxes and their detrended values as functions of L-shell and drift frequency. The Fourier results reveal that the zebra stripes are generated by electric-field perturbations about 9.85 h before (with a FWHM of approximately 2.8 h; Fig. 6j), coincident in time with the observations of the contiguous expansion.

Motivated by these observations, we perform the same test-particle simulation as for Event I, to examine whether a pulsed electric field can produce the observed signatures. The results, given in Supplementary Figs. 3 and 4, capture the contiguous expansion of the SAA electron environments. The trajectories of representative test electrons are also similar to those in Event I, except that the minimum mirror heights are higher than 100 km for both test electrons observed before 18:55 UT. This demonstrates that a more sustained or intense electric-field interaction can fill the gap between the vertical stripe and the nominal SAA region observed in Event I, thereby forming the contiguous expansion.

### Event III: Observations of recurrent vertical stripes

The events described above characterize the transient distortions of the SAA radiation environments either as a single, isolated vertical stripe (Event I) or as a contiguous longitudinal expansion (Event II). Interestingly, recurrent vertical stripes may also be observed along the LEO orbit. As will be shown below, the recurrent nature of these stripes indicates periodic electric-field perturbations associated with ultra-low frequency (ULF, in the mHz frequency range) waves^38–41^.

**Fig. 7:**
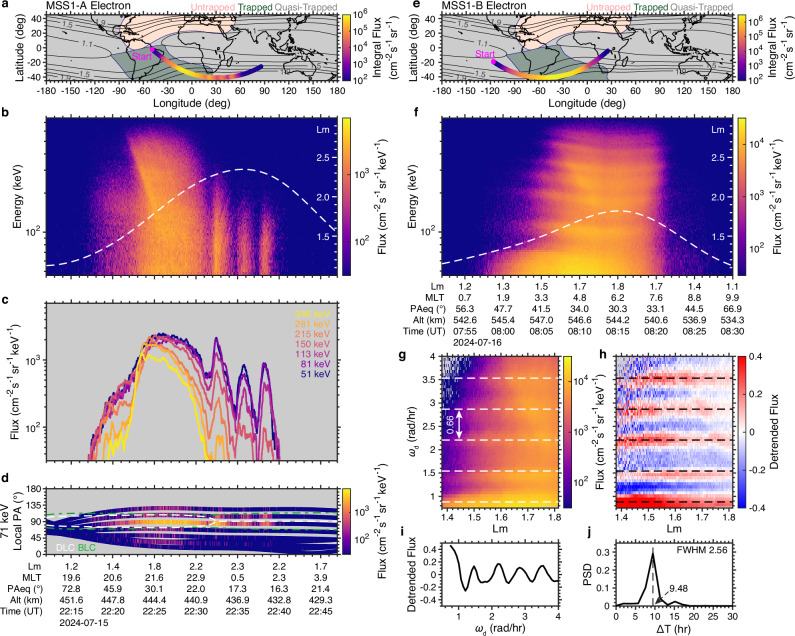
Overview of MSS1 observations for Event III with recurrent vertical stripes. The left panels display the observations from MSS1-A on July 15, 2024. **a** Satellite ground track, color-coded by the energy-integral fluxes of electrons with local pitch angle around 90^∘^. The background colormap indicates different trapping regions at the satellite’s orbital altitude, accompanied by McIlwain L-parameter contours with an interval of 0.2. **b** Energy-time spectrogram for electrons near 90^∘^ pitch angle, with the dashed white line representing L-shell variations. **c** Temporal variations of electron fluxes for several energy channels. **d** Local pitch-angle (PA) distributions for 71-keV electrons. The white dashed and green dash-dotted lines represent the drift loss cone (DLC) and bounce loss cone (BLC), respectively. The right panels exhibit MSS1-B observations for the zebra stripes several hours later on July 16, 2024, including **e** satellite ground track and **f** energy-time spectrograms, which are in the same format as (**a**, **b**), **g**,** h** electron fluxes and detrended values as a function of particle drift frequency (*ω*_d_) and L-shell, and **i**,** j** the L-shell-averaged detrended flux values and the corresponding power spectral density (PSD; in arbitrary units) from Fourier analysis, with the full-width half-maximum (FWHM) labeled. Source data are provided as a Source data file.

Figure 7 shows the MSS1-A observations on July 15, 2024 (Event III). Similar to previous events, MSS1-A first traverses the SAA and observes the enhanced fluxes of stably-trapped electrons (Fig. 7a–d). A sudden electron-flux reduction is observed around 22:31 UT, which occurs unexpectedly before the spacecraft reaches the nominal SAA boundary around 22:32 UT as indicated by the DLC expansion to 90^∘^ (see the dashed lines in Fig. 7d). More interestingly, MSS1-A observes another flux enhancement upon crossing the nominal SAA boundary around 22:32 UT, forming a vertical stripe in the energy-time spectrogram (Fig. 7b). This pattern of electron flux reduction and subsequent enhancement repeats two more times outside the nominal SAA boundary, manifesting as recurrent vertical stripes until the eventual flux depletion around 22:40 UT. These flux enhancements outside the nominal SAA region, as in previous events, are likely driven by electric-field perturbations. However, in the absence of other perturbations, such a westward-directed electric field would be expected to produce a contiguous extension (like in Event II). The fact that this extension is fragmented into periodic stripes implies a modulation mechanism that periodically inhibits and facilitates the electron descent to the satellite orbital altitude. Given that the observed periodicity (approximately 175 s) falls within the ULF range, we hypothesize that this modulation is driven, at least partially, by ULF waves. This hypothesis will be examined later.

Like in previous events, the presence of electric-field perturbations during the MSS1-A observations is supported by zebra-stripe observations from MSS1-B several hours afterwards (Fig. 7e, f). The same analysis as in previous events results in the observed electron fluxes and their detrended values as functions of L-shell and drift frequency, shown in Fig. 7g, h. Fourier analysis indicates the zebra-stripe formation by electric-field perturbations about 9.5 h earlier (with a FWHM of approximately 2.6 h), coincident in time with the recurrent vertical stripes observed by MSS1-A.

**Fig. 8:**
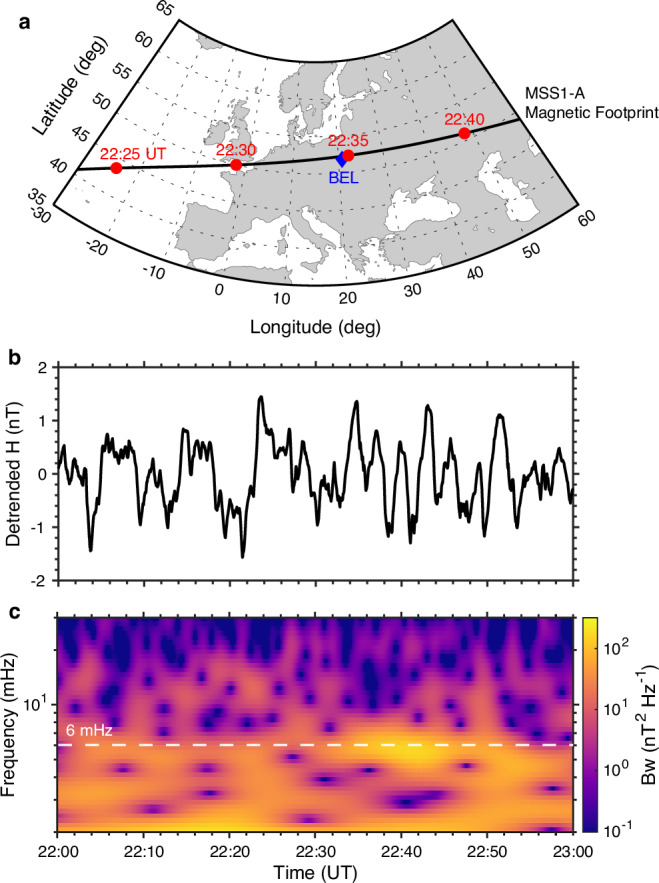
Measurements from ground-based magnetometer during Event III. **a** Geographic location of the magnetic observatory station BEL (Belsk; blue diamond) and the magnetic footprint of MSS1-A in the northern hemisphere (black line). The red dots correspond to the specific positions of MSS1-A at several moments. **b** Detrended observations of the H-components (magnetic northward) of the geomagnetic field at BEL station, with the 10-min sliding average background removed. **c** Time-frequency spectrogram of the magnetic field measurements at BEL, with the horizontal dashed line marking 6 mHz. Source data are provided as a Source data file.

To examine the hypothesis of ULF wave-induced modulation, we further analyze the ground-based magnetometer measurements. When MSS1-A observes the recurrent vertical stripes, its magnetic footprint in the northern hemisphere moves across the BEL (Belsk) station (Fig. 8a). The geomagnetic field measurements at BEL exhibit clear ULF wave signatures during its conjunction with MSS1-A, shown in Fig. 8b by the detrended H-component. The corresponding time-frequency spectrogram in Fig. 8c reveals a wave-power peak at 6 mHz (nearly 167 s). The temporal, spatial, and spectral coincidences between ground-based and space-borne observations are consistent with ULF waves contributing to the periodic distortion of the SAA radiation environments, manifesting as recurrent vertical stripes. This hypothesis is further supported by test-particle simulations in the next subsection.

### Event III: Simulations

**Fig. 9:**
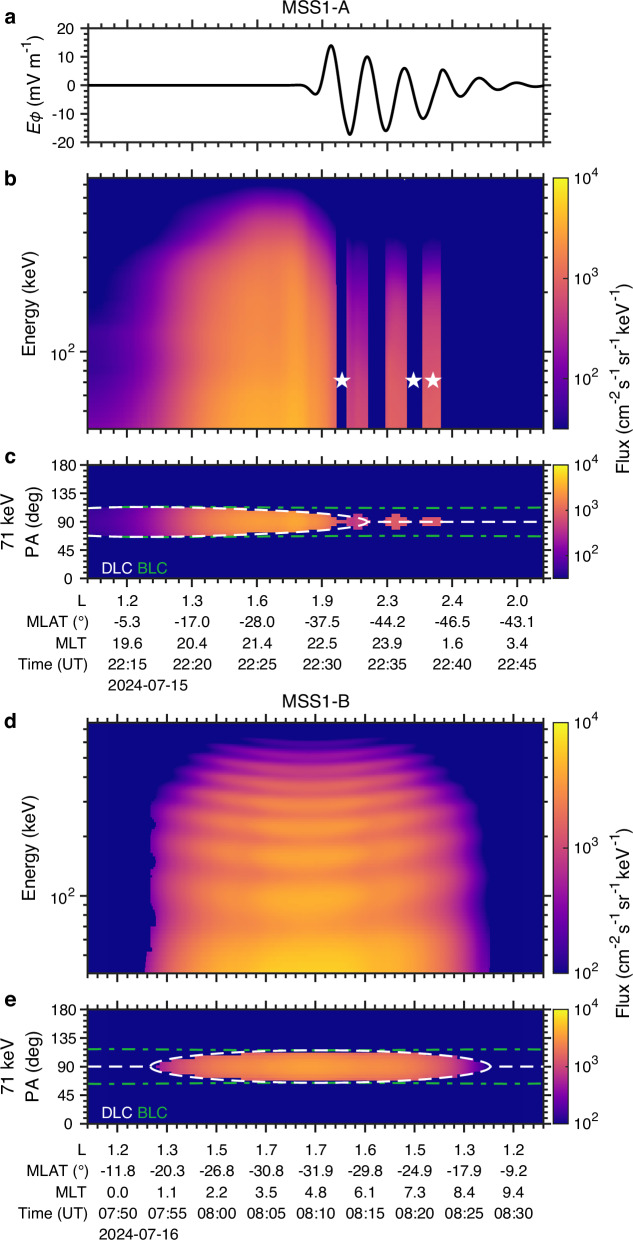
Simulation results for Event III with recurrent vertical stripes. **a** Equatorial electric-field perturbations along the trajectory magnetically connected to MSS1-A. **b** Simulated energy spectrogram of electron fluxes with local pitch angle 85^∘^ and **c** local pitch-angle (PA) distributions for 71-keV electrons along the MSS1-A trajectory. The star symbols denote three representative test electrons selected for detailed trajectory analysis below. The white dashed and green dash-dotted lines represent the drift loss cone (DLC) and bounce loss cone (BLC), respectively. **d** Simulated energy spectrograms of locally mirroring electrons and **e** local pitch-angle distributions for 71-keV electrons along the MSS1-B trajectory several hours later. The ephemeris data include the L-shell (L), magnetic latitude (MLAT), and magnetic local time (MLT). Source data are provided as a Source data file.

We follow the same simulation approach as for previous events to trace test electrons backward in time from the spacecraft, examining whether the observed flux variations can be reproduced. The electric-field perturbations are constructed as a combination of a pulsed electric field and ULF wave-carried perturbations (see Methods subsection “Electric field perturbations”). Figure 9a shows the modeled electric field along the magnetic footprints of MSS1-A on the equatorial plane, which exhibits clear ULF signatures. Under the effect of such electric-field perturbations, the simulated electron energy-time spectrogram (Fig. 9b) reproduces the recurrent vertical stripes, with flux enhancements confined near the local pitch angle of 90^∘^ (Fig. 9c). The agreement between simulations and observations demonstrates the role of ULF waves in the generation of recurrent vertical stripes. We further show in Fig. 9d, e the simulation results for zebra stripes observable along MSS1-B trajectories, again consistent with the observed features.

**Fig. 10:**
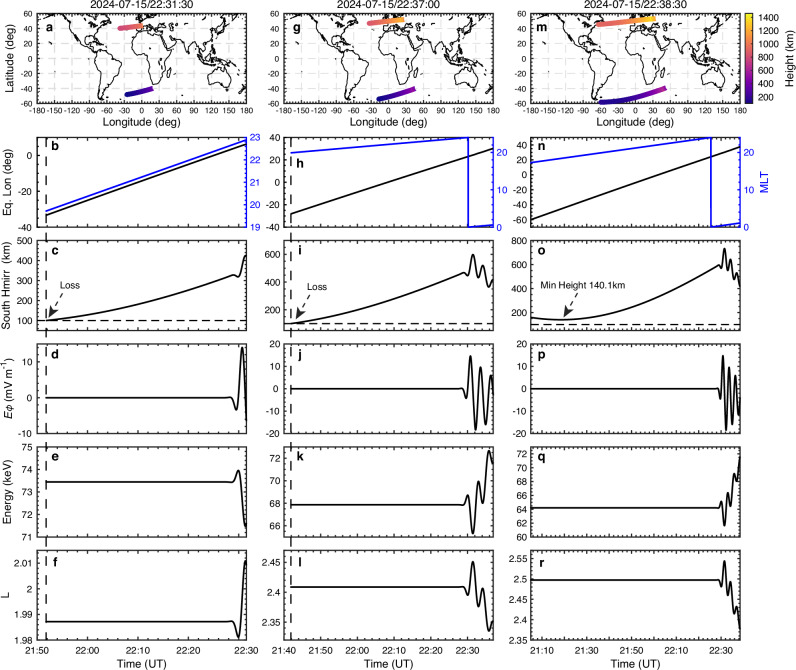
Variations of electrons in the test-particle simulation for Event III. The results displayed in the left column include **a** the geographical locations of mirror points in the northern and southern hemispheres with color representing the mirror heights, and the temporal variations of **b** the geographical longitude at magnetic equator (Eq. Lon; black) and magnetic local time (MLT; blue), **c** the mirror height (Hmirr) in southern hemisphere, **d** the encountered azimuthal electric field *E*_*ϕ*_, **e** the energy change, and **f** the L-shell. The middle column **g**–**l** and the right column **m**–**r** are in the same format as the left column, except that they display the simulation results for electrons observed at different times, as indicated at the top of each column. Source data are provided as a Source data file.

To better understand the flux reduction within the nominal SAA region and the periodic signatures outside, we show in Fig. 10 the trajectories and associated parameters of a few representative electrons until they encounter MSS1-A. The time and energy of their encounter are marked in Fig. 9b. Let us first focus on Fig. 10a–f, which correspond to the test electron associated with a reduced flux when reaching the spacecraft within the nominal SAA region. Before its spacecraft encounter, the electron’s mirror point (Fig. 10c) has been significantly elevated by the perturbed electric field (see the azimuthal electric field experienced by the electron in Fig. 10d) that decelerates the electron (Fig. 10e for energy variations). That is the reason why this electron originates from altitudes below 100 km around 21:52 UT (see Fig. 10c), which explains its low flux level in the energy spectrogram (Fig. 7b). In other words, the unusual flux reduction within the nominal SAA region is caused by the ULF wave-induced deceleration of electrons, which elevates their mirror heights so that the stably-trapped electrons can no longer access the spacecraft at lower altitudes.

The two rightmost columns of Fig. 10 display the results for electrons observed between the second and the third vertical stripes, and in the center of the third stripe, respectively. Both electrons have been accelerated (Fig. 10k, q) by the oscillatory electric-field perturbations (Fig. 10j, p) before their spacecraft encounter to the east of the nominal SAA boundary. The acceleration leads to an oscillatory decrease in their mirror heights (Fig. 10i, o). Note that the acceleration and the mirror-height reduction are more significant for the latter electron, since it experiences a westward-directed electric field during the final half-cycle of the ULF waves before reaching the spacecraft. In other words, the presence of the recurrent vertical stripes originates from the ULF wave-induced mirror-height variations of the stably-trapped electrons back-and-forth across the spacecraft altitude.

## Discussion

In this paper, we report transient distortions of the SAA radiation environments, manifesting as single/recurrent vertical stripes of energetic electron fluxes to the east of the nominal SAA boundary, or as contiguous longitudinal expansions of the SAA radiation environments. While electron-flux enhancements outside the SAA have been reported previously (like FEE events^28^), the features reported in this paper represent a distinct observational manifestation. They are interpreted as extensions of the SAA electron environments in response to large-scale electric-field perturbations. In our proposed scenario (see Fig. 1b), a westward-directed electric field leads to the acceleration of eastward-drifting electrons, and consequently, a reduction in their mirror heights. This process enables the stably-trapped electrons, originally mirroring at altitudes higher than the satellite orbit, to reach the spacecraft outside the nominal SAA boundary. As shown in the observations, the longitudinal extension could reach 30^∘^ in certain cases (Figs. 6 and 7).

The characteristics of these distortions (including their locations, durations, and energy ranges) provide significant, observation-driven constraints on the associated electric-field perturbations, which can be further quantified through comparison with modeling results. Observationally, the electron flux enhancements east of the nominal SAA boundary require that the stably-trapped electrons must have been accelerated by the perturbed electric field after reaching their lowest mirror heights (above 100 km, by definition of stable trapping) at the SAA center. Without a recent perturbation, backward tracing of the observed quasi-trapped electrons would imply mirror altitudes below 100 km within the SAA, where they would be lost by atmospheric precipitation. Their survival, therefore, requires the electric-field perturbation to occur after the electrons pass the lowest-mirror-height region, corresponding to an approximately 10-min window before the observation given the electron drift speeds. This timing requirement rules out the possibility that the spacecraft merely crosses a pre-existing electron-flux structure generated by an earlier perturbation, while the required mirror-height reduction further constrains the perturbation amplitude and duration.

Compared with the contiguous eastward expansion (Event II), the short-lived vertical stripe in Event I and its isolation from the nominal SAA region can provide further information about the electric-field perturbation. To quantitatively evaluate the robustness of these observation-driven constraints, we performed a sensitivity analysis of electric-field parameters applied in simulations for Event I (see Supplementary Fig. 5). Although the exact spatio temporal structure of the electric-field perturbations cannot be uniquely determined and the quantitative limits may be model-dependent, the results suggest a narrow domain of feasible parameter sets, with the representative field properties including an amplitude of several mV/m, a duration of about 10 min, and an onset within a restricted time window of minutes. A weaker electric-field interaction (due to a later onset, smaller amplitude, or shorter duration) is unable to produce the flux enhancements beyond the nominal SAA boundary, whereas a stronger interaction can transform the isolated vertical stripe into a contiguous expansion. These constraints reduce the margin of error in determining the onset time of electric-field perturbations compared to results derived from zebra stripes, and provide information about the electric-field amplitude and duration that previous methods could not offer.

Given that the transient distortions analyzed here are consistently accompanied by zebra stripes, the derived constraints also provide critical insights into the generation of these spectral features. The sensitivity analysis shows that a small-amplitude field (e.g., <1.5 mV/m), even if applied for a long duration, would be insufficient to lower the mirror points of stably-trapped electrons to the satellite altitude (see Supplementary Fig. 5n). This suggests that the generation of zebra stripes, at least in some events, is associated with pulsed^30^, rather than small-amplitude, long-duration, electric fields^32^. The relationship between zebra stripes and different types of electric-field perturbations is a topic for future studies.

The origin of these large-scale electric-field perturbations is another point that requires further investigation. Previous studies have utilized zebra-stripe observations to show that the penetration electric fields usually emerge during the substorm onset phase^21^. Consistent with this scenario, Events II and III indeed occur when AE index was about to rise (see Supplementary Fig. 6), indicating the upcoming substorm activity. Event I, however, corresponds to a quiet substorm condition. A notable signature possibly associated with this event is the southward turning of the interplanetary magnetic fields (IMF), which has been proposed previously as a potential source of penetration electric field^42^. Interestingly, both Events II and III are also accompanied by southward IMF. Future studies are required to determine the relative contributions of solar-wind disturbances and substorm activities to electric-field perturbations in the inner belt.

Besides pulsed electric fields, the distortions of the SAA radiation environments can also be modulated by ULF waves, manifesting as recurrent vertical stripes (Event III). The consistency between the 6-mHz wave frequency at BEL and the 175-s periodicity of the recurrent vertical stripes suggests that our simple ULF-wave model is sufficient to test the plausibility of the proposed mechanism, although the modeled wave amplitude is higher than estimates mapped directly from ground-based signals^43^. This discrepancy, however, could be partly attributed to the ionospheric screening or a significant compressional wave component. This approach provides a bridge to link the ground-based observations and in-situ particle signatures. Nevertheless, a fully self-consistent treatment of ULF wave dynamics remains an important target for future studies.

Moreover, we note that the electrons outside the nominal SAA boundary may be observed again near the western edge after drifting around the Earth, where they experience another descent in mirror heights. In this sense, the transient distortions may also appear to the west of the nominal SAA region, although it may not manifest as vertical stripes since the electrons undergo energy dispersion during their drift motion (see an example in Supplementary Fig. 7). These electrons would eventually precipitate into the atmosphere within the SAA, since their pitch angles, after the acceleration and descending process, fall within the drift loss cone. Together, these results demonstrate that radiation threats from inner-belt electrons to LEO spacecraft are not confined to the nominal SAA region but can extend dynamically beyond it.

Finally, beyond the specific case studies, this work suggests that these transient distortions of the SAA radiation environments can, in principle, serve as a diagnostic tool. By treating the eastern boundary of the nominal SAA region as a sensitive detector, one may utilize similar LEO particle observations combined with backward-tracing simulations^36^ to remotely sense and constrain the properties of transient electric fields in the inner belt, which are otherwise challenging to measure. Acknowledging our specific modeling framework and limited event sample, we present this as a promising yet still developing interpretation. While establishing a broadly applicable diagnostic framework requires future statistical studies, these findings collectively reveal a distinct mode of the inner belt variability, providing valuable insights into near-Earth space weather and critical considerations for spacecraft protection.

## Methods

### Macau Science Satellite-1

The Macau Science Satellite-1 (MSS1) mission consists of two satellites operating in low-Earth orbit with an inclination of 41^∘^ and an orbital period around 94 min. MSS1-A follows a quasi-circular orbit at an altitude of approximately 450 km, whereas MSS1-B operates in an elliptical orbit with altitudes varying between 450 and 500 km. Both satellites provide energetic electron measurements via the Medium-energy Electron Spectrometer (MES)^44^. The MES features nine sensor heads that measure electrons within 40–750 keV from different directions simultaneously, achieving high angular, temporal (1 s cadence), and energy (≤8.55% at 100 keV) resolutions. Each sensor head has a full-width half-maximum (FWHM) of approximately 10^∘^ in angular coverage. For our analysis of the transient distortions of the South Atlantic Anomaly radiation environments, we utilize the level-2 scientific data during three specific event periods: 9–10 April 2024, 15–16 July 2024, and 24–25 September 2024. These data products have been routinely processed and calibrated by the instrument team prior to public release.

### Zebra stripe analysis

To rearrange the observed zebra stripes into a spectrogram as a function of L-shell and electron drift frequency, we first set up the corresponding grids and calculate the average electron flux within each grid cell. Here, we use the McIlwain L-parameter and the equatorial pitch angle, calculated in the IGRF^37^ that corresponds to the local 90^∘^ pitch angle at the spacecraft position, to estimate the electron magnetic drift frequencies based on the following formula^2^:

1 $${\omega }_{B}=\frac{3L}{e{B}_{E}{R}_{E}^{2}}\frac{W(W+2{E}_{0})}{W+{E}_{0}}\frac{D(y)}{T(y)},$$

where *L* is the L-shell parameter, *e* is the electron charge, *W* is the electron kinetic energy, *E*_0_ = 0.511MeV is the electron rest energy, *B*_*E*_ is the equatorial magnetic field strength at Earth’s surface, *R*_*E*_ = 6371.2 km is Earth’s radius, and $y=\sin ({\alpha }_{{{{\rm{eq}}}}})$, with *α*_eq_ representing the electron’s equatorial pitch angle. Here, *T*(*y*) and *D*(*y*) are approximately expressed by

2 $$T(y)\simeq {T}_{0}-\frac{1}{2}({T}_{0}-{T}_{1})(y+{y}^{\frac{1}{2}}),$$

3 $$Y(y)\simeq 2(1-y){T}_{0}+({T}_{0}-{T}_{1})(y\ln y+2y-2{y}^{\frac{1}{2}}),$$

4 $$D(y)=\frac{1}{2}T(y)-\frac{1}{12}Y(y),$$

where *T*_0_ = 1.3802 and *T*_1_ = 0.7405. The drift frequency of energetic electrons *ω*_d_ is the sum of the magnetic drift *ω*_*B*_ and the corotation drift *ω*_c_, where the latter is approximately the same as Earth’s rotation, *π*/12 rad/h.

### Simulation setup

The trajectories of energetic electrons are traced within an eccentric tilted dipole (ETD) magnetic field in this study, which is constructed based on the Gauss coefficients of the IGRF-14 model. The dipole axis orientation in the Cartesian geographic coordinate system is given by $(-\frac{{g}_{1}^{1}}{{B}_{0}},-\frac{{h}_{1}^{1}}{{B}_{0}},-\frac{{g}_{1}^{0}}{{B}_{0}})$^45^, where ${g}_{1}^{0},{g}_{1}^{1},{h}_{1}^{1}$ are the first three IGRF coefficients, and ${B}_{0}=\sqrt{{({g}_{1}^{0})}^{2}+{({g}_{1}^{1})}^{2}+{({h}_{1}^{1})}^{2}}$ represents the equatorial magnetic strength at a radial distance of 1 *R*_*E*_, equivalent to *B*_*E*_ in Eq. (1). This provides a dipole tilt angle of approximately 9.24^∘^. To better simulate the electron motion near the SAA region, we determine the optimal displacement of the dipole center from Earth’s center by minimizing the relative magnetic strength difference at 100 km altitude between the ETD field and full IGRF model within a latitude range of [−45^∘^, −10^∘^] and a longitude range of [−100^∘^, 150^∘^]. This optimization ensures that the simulated SAA boundary location and the drift loss cone variations along the satellite orbit closely match those of the full IGRF-14 model, particularly in the eastern sector (compare Figs. 2d and 4b). While the ETD approximation reproduces the large-scale field structure of the SAA with good accuracy, it does not capture higher-order IGRF harmonics. Consequently, our simulation results should be interpreted as robust for large-scale signatures, but not as precise representations of fine-scale field structure.

We next construct the eccentric-tilted SM coordinate system based on the orientation of the dipole axis, the optical dipole center displacement, and the Sun’s orientation relative to Earth. The origin of the coordinate system is the ETD center, with the *Z*-axis parallel to the dipole axis pointing northward. The *Y*-axis is perpendicular to both the *Z*-axis and the Earth-Sun line, and the *X*-axis completes the right-handed triad.

In such an eccentric-tilted SM coordinate system, the electron guiding-center motion^46^ can be described by the temporal variations of the L-shell parameter *L*, the azimuthal angle *ϕ*, and the equatorial pitch angle *α*_eq_ as^2^

5 $$\frac{{{{\rm{d}}}}L}{{{{\rm{d}}}}t}=\frac{1}{{R}_{E}}\frac{{E}_{\phi }}{B},$$

6 $$\frac{{{{\rm{d}}}}\phi }{{{{\rm{d}}}}t}={\omega }_{B}+{\omega }_{{{{\rm{c}}}}},$$

7 $$\frac{{{{\rm{d}}}}y}{{{{\rm{d}}}}t}=-\frac{Y(y)}{4T(y)}\frac{y}{L}\frac{dL}{dt},$$

where *ω*_*B*_ is the magnetic drift frequency given by Eq. (1), *ω*_c_ = *π*/12 rad/h is the corotation drift frequency, $y=\sin ({\alpha }_{{{{\rm{eq}}}}})$, *T*(*y*) and *Y*(*y*) are functions of pitch angle given by Eqs. (2) and (3), *B* is the local magnetic field strength, and *E*_*ϕ*_ is the azimuthal electric field perturbations to be modeled in the next subsection.

In our simulation, the energetic electrons are traced backward in time^36^ from the observation point (the satellite position) to the time before the electric-field perturbations appear. At each step of backward tracing, the distance between the electron’s mirror point and the Earth’s center is calculated, so that those electrons reaching the mirror points below 100 km can be identified and assigned a zero flux (since they are considered lost to the atmosphere). For electrons completing the full backward tracing process, their fluxes are determined via Liouville’s theorem^47^ that the phase space density (PSD) is conserved along the particle’s traced trajectory. Therefore, their final phase space densities *f* are given by their initial states via $f=j/{p}^{2}={j}_{0}/{p}_{0}^{2}$, where *j* and *p* are the flux and electron momentum at the satellite position, and *j*_0_ and *p*_0_ are the values at the initial location before electric-field perturbations. The initial electron distributions are constructed based on observations from both MSS1-A and MSS1-B within the previous month, as functions of energy, equatorial pitch angle, and L-shell. Note that at each time point, a moving average of the observed fluxes is carried out over energy to smooth out the effect of zebra stripes.

### Electric field perturbations

To simulate the effect of large-scale electric field perturbations, a pulsed electric field in the dawn-dusk direction is modeled in the eccentric-tilted SM coordinates (*r*, *θ*, *ϕ*). Here we focus on its azimuthal component in the equatorial plane, given by

8 $${{{{\bf{E}}}}}_{{{{\rm{c}}}}}=(a{L}^{2}+bL+c)\cos \phi f(t){\hat{{{{\bf{e}}}}}}_{\phi },$$

9 $$f(t)=\frac{\tanh [\alpha (t-{t}_{0}+D)]+\tanh [\alpha (-t+{t}_{0}+D)]}{2\tanh (\alpha D)},$$

where *L* is the L-shell parameter, *ϕ* is the azimuthal angle, ${\hat{{{{\bf{e}}}}}}_{\phi }$ points eastward positively, *a*, *b*, *c* (in mV m^−1^) control the radial distribution of the electric perturbations (with the field magnitude truncated to zero where the quadratic term yields negative values), *f*(*t*) determines the temporal profile, *α* controls the increase rate at the onset of the electric field, *t*_0_ is the center time, and *D* is the half-duration of the electric pulse.

For Event I characterized by a single vertical stripe, we set *a* = − 91, *b* = 320, *c* = − 275.2, and *α* = 0.1. *t*_0_ is set to 21:25 UT on April 09, 2024, and *D* is 240 s. This generates an electric-field pulse with a maximum amplitude of approximately 6 mV m^−1^ and a duration of 8 min.

For Event II with contiguous SAA expansions, we set *a* = −1.5, *b* = 8, *c* = −6.5, and *α* = 0.1. *t*_0_ is set to 18:50 UT on September 24, 2024, and *D* is 240 s. This corresponds to an electric-field pulse with a maximum amplitude of approximately 4 mV m^−1^ and a duration of 8 min.

For Event III featuring recurrent vertical stripes, we set *a* = −1.5, *b* = 8, *c* = −6.5, and *α* = 0.1, same as the settings for Event II. *t*_0_ is set to 22:35:30 UT on July 15, 2024, and *D* is 210 s. This corresponds to an electric-field pulse with a maximum amplitude of approximately 4 mV m^−1^ and a duration of 7 min.

In addition to the electric-field pulse, the electric perturbations carried by ULF waves are also superposed for Event III. Here we follow the procedure in Zhou et al.^48^ to construct a wave-associated electric field during the wave growth and damping phases, expressed by

10 $${{{{\bf{E}}}}}_{{{{\rm{ULF}}}}}={E}_{0}\exp \left(-\frac{{(t-{t}_{0})}^{2}}{{\tau }^{2}}\right)\exp {{{\rm{i}}}}(m\phi -\omega t+\psi ){\hat{{{{\bf{e}}}}}}_{\phi },$$

where *E*_0_ represents the maximum wave amplitude at *t* = *t*_0_, *τ* controls the time scale of wave growth and decay, *ω* is the wave angular frequency, *m* is the azimuthal wave number, and *ψ* is a phase shift.

The ground-based magnetic observations during Event III indicate a wave frequency of 6 mHz (*ω* = 0.012*π*). Moreover, the period of the recurrent vertical stripes (approximately 175 s) could be considered the Doppler-shifted wave frequency observed by the orbiting satellite, i.e., *ω*_obs_ = 2*π*/175. Given the azimuthal satellite velocity *ω*_s_ = 0.0014, we can estimate the azimuthal wave number as *m* = (*ω* ± *ω*_obs_)/*ω*_s_, yielding two results about 1 and 53, both of which seem reasonable. However, according to the drift resonance condition^39,40^, *ω* = *mω*_d_, where d is the particle drift frequency, a larger wave number corresponds to a lower drift-resonant energy. Furthermore, the electron energy change caused by ULF waves would typically have a 180-degree phase difference across the drift-resonant energy, resulting in tilted signatures in the energy spectrogram. This is different from the vertical stripes without discernible energy dispersion at MSS1-A, which suggests that the resonant energy is not covered by the observational range within 40–200 keV. Since the azimuthal wave numbers of 1 and 53 correspond to the drift-resonant energies of about 17 MeV and 190 keV, respectively, it appears that *m* = 1 is a more realistic solution, which is applied in the simulation.

To represent the wave growth and decay stage, we also set *τ* = 80 s when *t *≤ *t*_0_ and *τ* = 500 s when *t *≥ *t*_0_, with *t*_0_ being 22:31 UT on July 15, 2024. The *E*_0_ value is set as 15 mV/m. The phase shift *ψ* is selected such that the wave phase encountered by MSS1-A at the center of the third vertical stripe (at 22:38:30 UT) is *π*/2. We note that this Gaussian-envelope, single-mode representation of ULF waves is intended as a simplified driver. It captures the frequency peak in observations and reproduces the periodicity of the recurrent vertical stripes, but should not be regarded as a self-consistent global wave model.

## Supplementary information

Supplementary Information

Transparent Peer Review file

## Source data

Source Data

## Data Availability

The observational data from the Macau Science Satellite-1 (MSS1) mission that support the findings of this study have been deposited in Zenodo^49^ (10.5281/zenodo.18277877). The magnetic measurements at the BEL (Belsk) station used in this study are accessible at INTERMAGNET\BEL Observatory\Best available\Second data (https://imag-data.bgs.ac.uk/GIN_V1/GINForms2). The solar wind parameters and geomagnetic indices used in this study are available in Coordinated Data Analysis Web (CDAWeb)\OMNI\OMNI HRO 1MIN and the GFZ Helmholtz Centre for Geosciences database\Kp and ap since 1932 (https://kp.gfz.de/en/data). The datasets generated in this study are provided in the Source Data file. Source data are provided with this paper.

## Code availability

The test-particle simulation codes that support the findings of this study are available at GitHub (https://github.com/Dexterous-Yin/Test_Particle_Simulation_Eccentric_Dipole) and have been archived at Zenodo^50^ (10.5281/zenodo.18279416). The data processing and visualization were performed using MATLAB (version R2024b). We also acknowledge the use of the IRBEM library (version 5.0.0) for data processing, the latest version of which is available at 10.5281/zenodo.6867552.
