# Supplementary material for: Transient distortions of the South Atlantic Anomaly radiation environments driven by electric fields
Source: Nat Commun. 2026 Jul 3;17:8345. doi: 10.1038/s41467-026-74984-z (PMC13473148; doi:10.1038/s41467-026-74984-z)
Supplement: Supplementary file 1 [file 41467_2026_74984_MOESM1_ESM.pdf]

Supplementary Information for

Transient Distortions of the South Atlantic Anomaly Radiation Environments Driven by Electric Fields

Ze-Fan Yin (尹泽藩)[1,2,3], Yi-Xin Sun (孙翌馨)[1,2], Xu-Zhi Zhou (周煦之)[1*],
Qiu-Gang Zong (宗秋刚)[2,1], Ying Liu (刘鹰)[2], Ze-Jun Hu (胡泽骏)[3],
Yoshiharu Omura (大村善治)[4,2], Robert Rankin[5], Hong Zou (邹鸿)[1], Yu-Guang Ye (叶雨光)[2]

[1*]School of Earth and Space Sciences, Peking University, Beijing, 100871, China.
[2]State Key Laboratory of Lunar and Planetary Sciences, Macau University of Science and Technology, Macau, 999078, China.
[3]Center for Space Physics and Astronomy, Polar Research Institute of China, Shanghai, 200136, China.
[4]Research Institute for Sustainable Humanosphere, Kyoto University, Kyoto, 611-0011, Japan.
[5]Department of Physics, University of Alberta, Edmonton, AB, T6G-2H5, Canada.

[*]Corresponding author(s). E-mail(s): xzzhou@pku.edu.cn

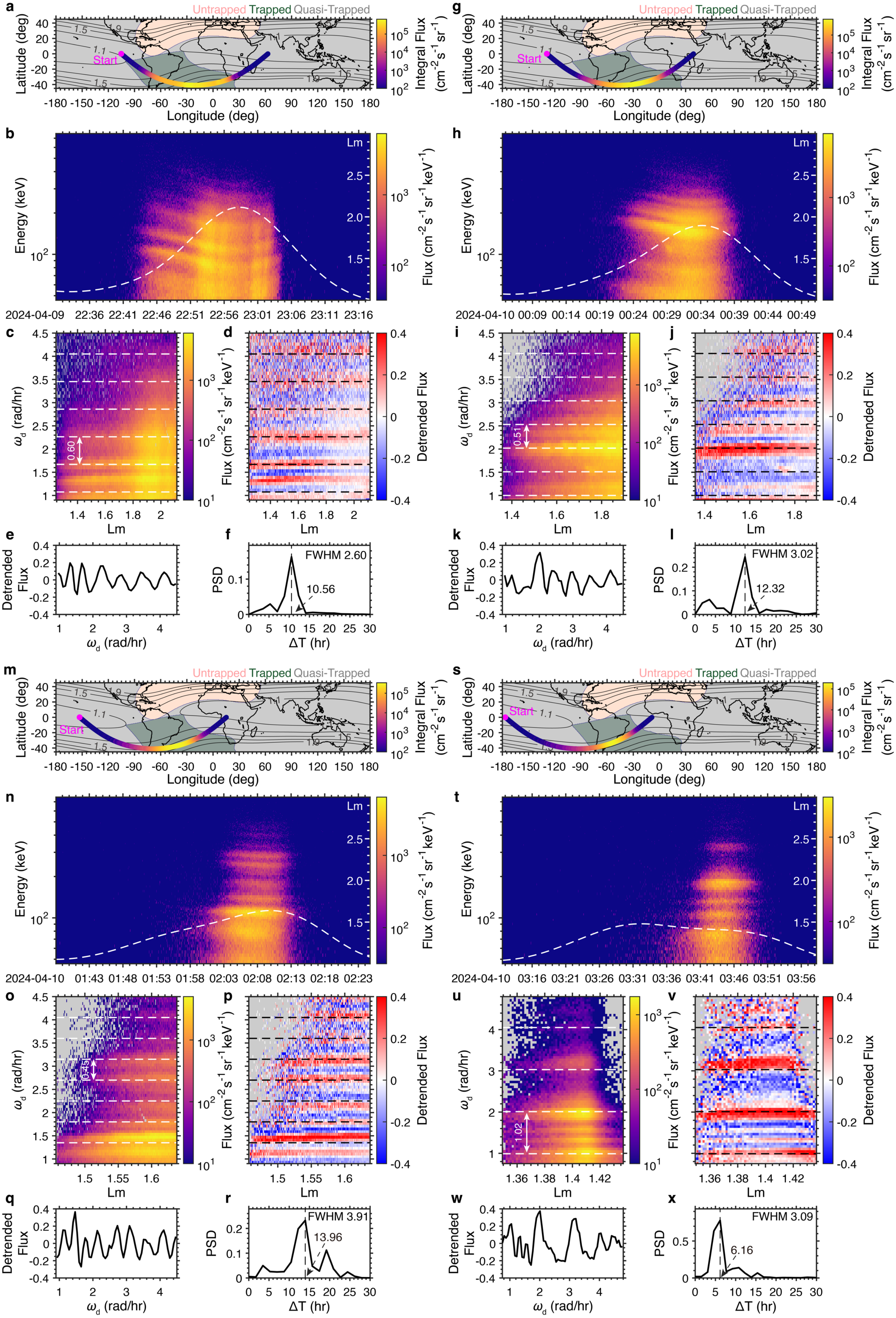
a
Untrapped Trapped Quasi-Trapped
Start
Latitude (deg)
Longitude (deg)
Integral Flux (cm^{-2}s^{-1}sr^{-1})
b
Energy (keV)
Lm
Flux (cm^{-2}s^{-1}sr^{-1}keV^{-1})
2024-04-09 22:36 22:41 22:46 22:51 22:56 23:01 23:06 23:11 23:16
c
d
ω_d (rad/hr)
0.60
Detrended Flux
Lm
e
f
ω_d (rad/hr)
PSD
FWHM 2.60
10.56
ΔT (hr)
g
Untrapped Trapped Quasi-Trapped
Start
h
2024-04-10 00:09 00:14 00:19 00:24 00:29 00:34 00:39 00:44 00:49
i
j
0.51
k
l
FWHM 3.02
12.32
m
Untrapped Trapped Quasi-Trapped
Start
n
2024-04-10 01:43 01:48 01:53 01:58 02:03 02:08 02:13 02:18 02:23
o
p
0.45
q
r
FWHM 3.91
13.96
s
Untrapped Trapped Quasi-Trapped
Start
t
2024-04-10 03:16 03:21 03:26 03:31 03:36 03:41 03:46 03:51 03:56
u
v
1.02
w
x
FWHM 3.09
6.16

Supplementary Fig. 1 Energetic electron measurements from MSS1-A for four consecutive orbits following the vertical stripe event on April 09, 2024 (Event I). The figure is organized into four panel groups, a-f, g-l, m-r, and s-x, in the same format, with each representing a distinct pass through the SAA region. For each pass (e.g., panel group a-f), the displayed observations include: **a** the satellite ground track, color-coded by the energy-integral fluxes of electrons with local pitch angle around 90° (the background colormap indicates different trapping regions at the satellite's orbital altitude, accompanied by McIlwain L-parameter contours with an interval of 0.2); **b** the energy-time spectrogram for electrons near 90° pitch angles, with the dashed white line representing L-shell variations; **c** the electron flux and **d** the detrended values as a function of particle drift frequency (ω_d) and L-shell; **e** the L-shell-averaged detrended flux values and **f** the corresponding power spectral density (PSD; in arbitrary units) from Fourier analysis. Note that the vertical stripe in Fig. 2b of the main text is no longer visible in these subsequent orbits. The zebra-stripe analysis reveals a transition in the dominant drift-periodic features. For the first three orbits (panels a-r), the stripes correspond to an earlier perturbation with an estimated onset time around 12:10 UT on April 09. By the fourth orbit (panels s-x), the zebra stripes generated by the electric field responsible for Event I become dominant, yielding an estimated onset time around 21:30 UT, consistent with the vertical stripe occurrence. Note the uncertainties for these derived onset times are on the order of hours, as indicated by the full-width half-maximum (FWHM) of the Fourier power distributions.

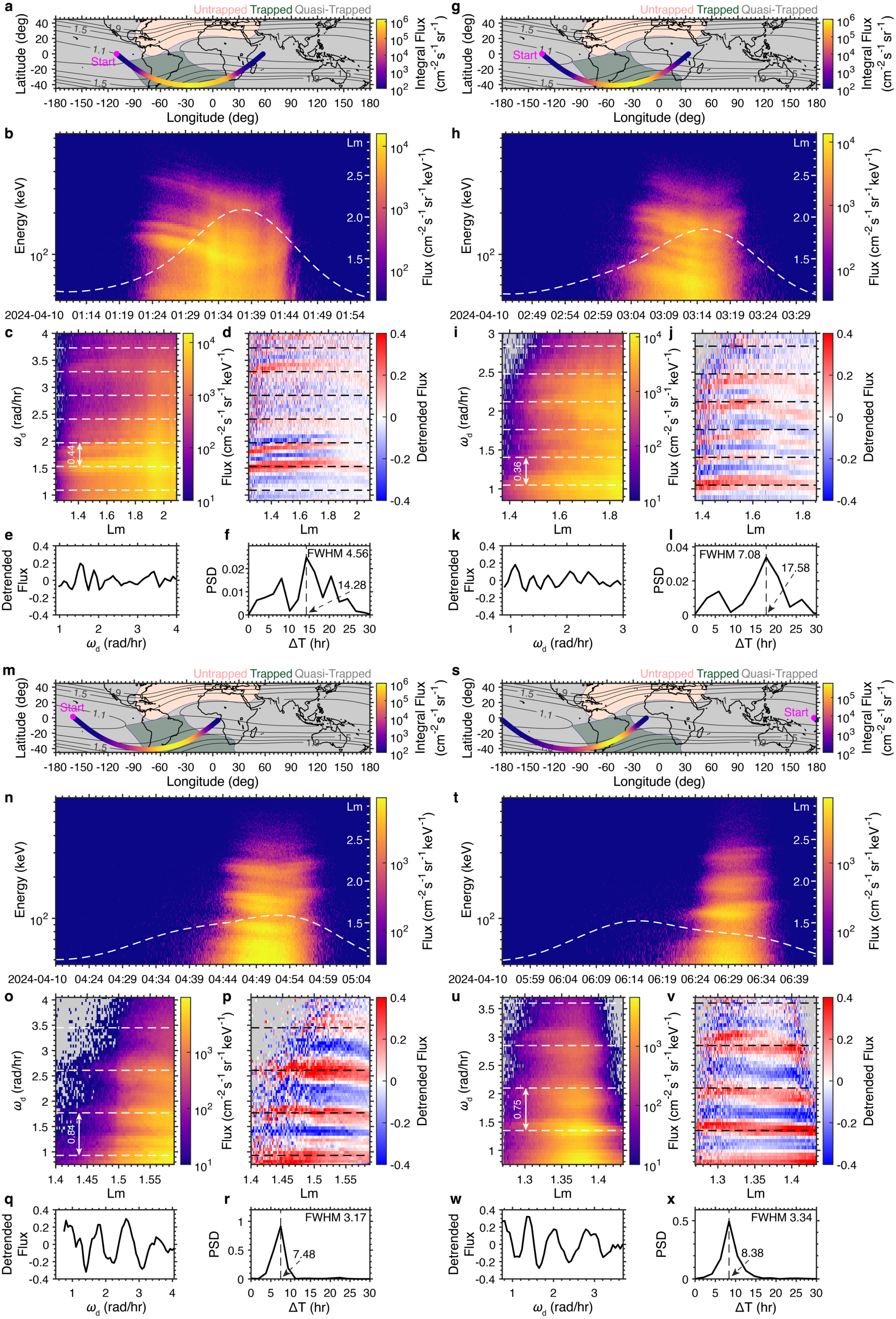
a
Untrapped Trapped Quasi-Trapped
Start
Latitude (deg)
Longitude (deg)
Integral Flux (cm^-2 s^-1 sr^-1)
b
Energy (keV)
Lm
Flux (cm^-2 s^-1 sr^-1 keV^-1)
2024-04-10 01:14 01:19 01:24 01:29 01:34 01:39 01:44 01:49 01:54
c
ω_d (rad/hr)
0.44
Lm
d
Detrended Flux
e
Detrended Flux
ω_d (rad/hr)
f
FWHM 4.56
14.28
PSD
ΔT (hr)
g
Untrapped Trapped Quasi-Trapped
Start
h
2024-04-10 02:49 02:54 02:59 03:04 03:09 03:14 03:19 03:24 03:29
i
0.36
j
k
l
FWHM 7.08
17.58
m
Untrapped Trapped Quasi-Trapped
Start
n
2024-04-10 04:24 04:29 04:34 04:39 04:44 04:49 04:54 04:59 05:04
o
0.84
p
q
r
FWHM 3.17
7.48
s
Untrapped Trapped Quasi-Trapped
Start
t
2024-04-10 05:59 06:04 06:09 06:14 06:19 06:24 06:29 06:34 06:39
u
0.75
v
w
x
FWHM 3.34
8.38

Supplementary Fig. 2 Energetic electron measurements from MSS1-B for four consecutive orbits. The figure is organized into four panel groups, a-f, g-l, m-r, and s-x, in the same format, with each representing a distinct pass through the SAA region. For each pass (e.g., panel group a-f), the displayed observations include: **a** the satellite ground track, color-coded by the energy-integral fluxes of electrons with local pitch angle near 90° (the background colormap indicates different trapping regions at the satellite's orbital altitude, accompanied by McIlwain L-parameter contours with an interval of 0.2); **b** the energy-time spectrogram for electrons around 90° pitch angles, with the dashed white line representing L-shell variations; **c** the electron flux and **d** the detrended values as a function of particle drift frequency (ω_d) and L-shell; **e** the L-shell-averaged detrended flux values and **f** the corresponding power spectral density (PSD; in arbitrary units) from Fourier analysis. The zebra stripe analysis also displays the transition of the dominant electric field perturbations. The features in the first two orbits (panels a-l) yield estimated onset times that are mostly consistent with the earlier perturbation derived from MSS1-A observations (around 12:10 UT on April 09; see Supplementary Fig. 1), considering the inherent uncertainties. As the satellite progresses to the last two orbits (panels m-x), the signatures induced by the new electric field perturbation take over, indicating an onset time around 21:30 UT on April 09. This is consistent with the vertical stripe occurrence (Event I) and the results derived from MSS1-A.

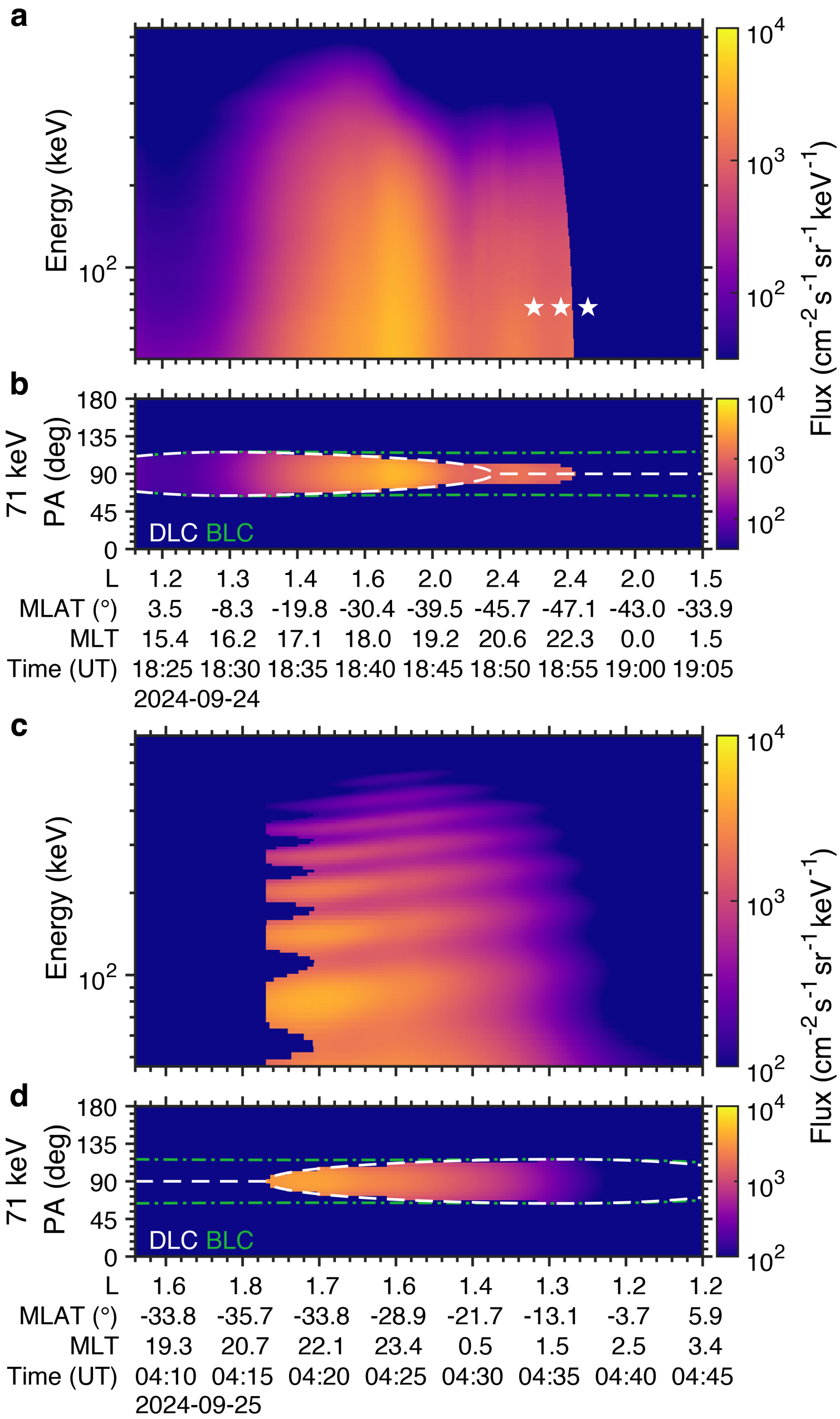

Supplementary Fig. 3 Simulation results for Event II characterized by the contiguous expansion of the SAA electron environment. **a-b** Simulated energy spectrogram of electron fluxes with local pitch angle 85° and local pitch-angle (PA) distributions for 71-keV electrons at MSS1-B's positions. The star symbols denote three representative test electrons selected for detailed trajectory analysis below. The white dashed and green dash-dotted lines represent the drift loss cone (DLC) and bounce loss cone (BLC), respectively. **c-d** Corresponding simulation results for zebra stripes observed several hours later, including the energy spectrograms of locally mirroring electrons and local pitch-angle distributions for 71-keV electrons. The ephemeris data include the L-shell (L), magnetic latitude (MLAT), and magnetic local time (MLT).

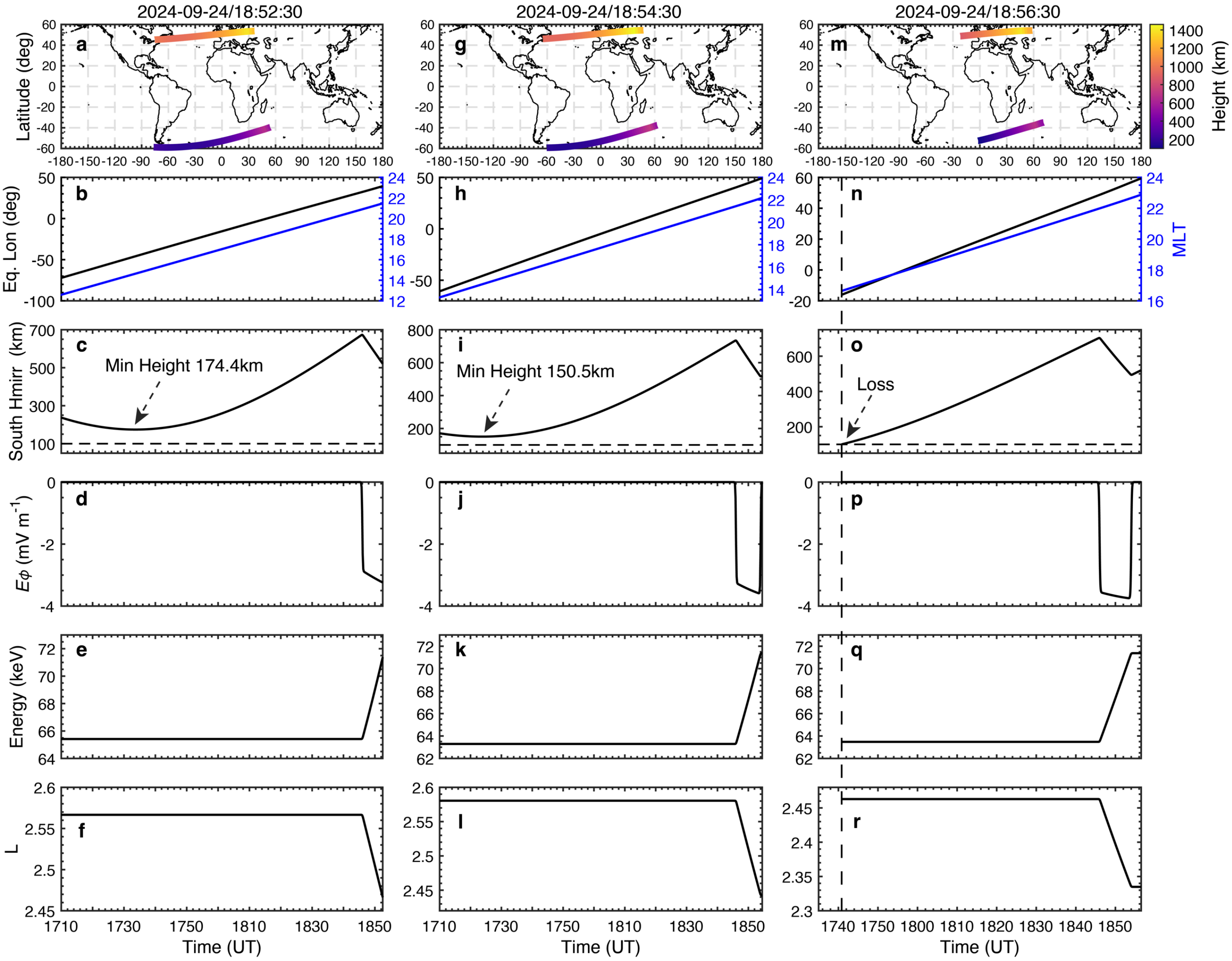

Supplementary Fig. 4 Variations of simulated electrons for Event II. The three columns present results for representative electrons marked by the star symbols in Supplementary Fig. 3a, respectively. The left column shows **a** the geographical locations of mirror points in the northern and southern hemispheres, color-coded by the mirror heights, and the temporal variations of **b** the geographical longitude at magnetic equator (Eq. Lon; black) and magnetic local time (MLT; blue), **c** the mirror height (Hmirr) in southern hemisphere, **d** the encountered azimuthal electric field, **e** the energy change, and **f** the L-shell. The middle column **g-l** and right column **m-r** follow the same format as panels a-f, except for electrons encountering the satellite at different times, as indicated at the top of each column. Compared to the simulation results for Event I with the detached vertical stripe (Figs. 4 and 5 in the main text), the test electron in the intermediate region of the seamless expansion (panels a-f) experiences a sufficiently strong electric field interaction to reduce its mirror height to the satellite altitude while remaining above the 100 km precipitation boundary (see panel c). This prevents the formation of an intermediate flux reduction, resulting in the contiguous eastward expansion of the SAA electron environment observed by the satellite.

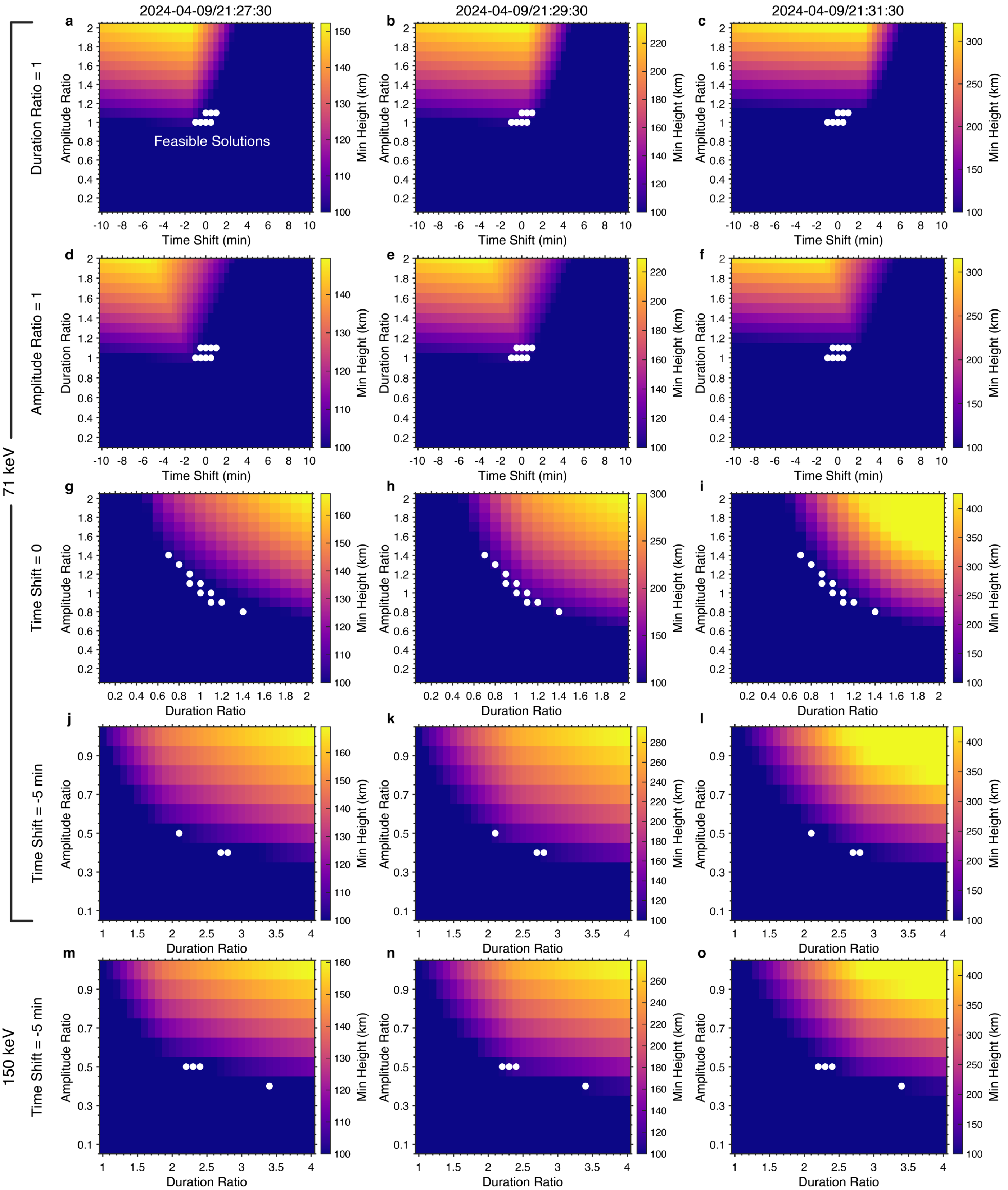

Supplementary Fig. 5 Sensitivity analysis of the electric field parameters in simulations for Event I. Each panel displays the minimum mirror height of test electrons traced backward in time under varying electric-field parameter sets. The 100 km threshold marks the atmospheric precipitation boundary. Values below 100 km (dark purple regions) indicate electron loss, corresponding to zero flux in the simulated energy-time spectrogram, while heights above 100 km indicate electrons detectable at the satellite's position. The parameters (amplitude, duration, time shift) are expressed as ratios or offsets relative to the settings used in the main text (where ratios=1 and time shift=0). The three columns correspond to representative test electrons encountering MSS1-A before (left), during (middle), and after (right) the vertical stripe appearance (same selection as in Fig. 5 of the main text). The first two rows **a-f** show the sensitivity results to time shifts for 71-

keV electrons, with **a-c** fixing Duration Ratio = 1 and **d-f** fixing Amplitude Ratio = 1. The white dots mark the feasible solutions that simultaneously satisfy the observational constraints: only the middle column shows mirror heights >100 km (vertical stripe observed), while the side columns show heights <100 km (flux reduction). These results constrain the onset time precision to the order of minutes. The third row **g-i** assesses sensitivity to the electric-field duration and amplitude under Time Shift = 0. The feasible parameters all correspond to a pulsed electric field of several mV/m and duration in minutes. The bottom two rows compare results for **j-l** 71-keV and **m-o** 150-keV electrons with Time Shift = -5 min. Note that parameter sets satisfying the 71-keV constraints may fail to satisfy the 150-keV constraints (the white dots in panels j-l and m-o do not completely overlap), as higher-energy electrons require a stronger electric field interaction due to their faster drift velocity. This sensitivity analysis reveals that a limited electric field interaction (due to a later onset, small amplitude or short duration) fails to generate a minimum mirror height above 100 km, which means it is insufficient to cause the flux enhancement outside the traditional SAA region. Crucially, panel n shows that a small-amplitude electric field (amplitude ratio <0.3), even with a very long duration, is insufficient to lower the mirror points of 150-keV electrons to the satellite altitude after they drift out of the SAA. This imposes a strict lower limit on the electric field amplitude, effectively ruling out the hypothesis that a small-amplitude field with long duration could generate the observed features.

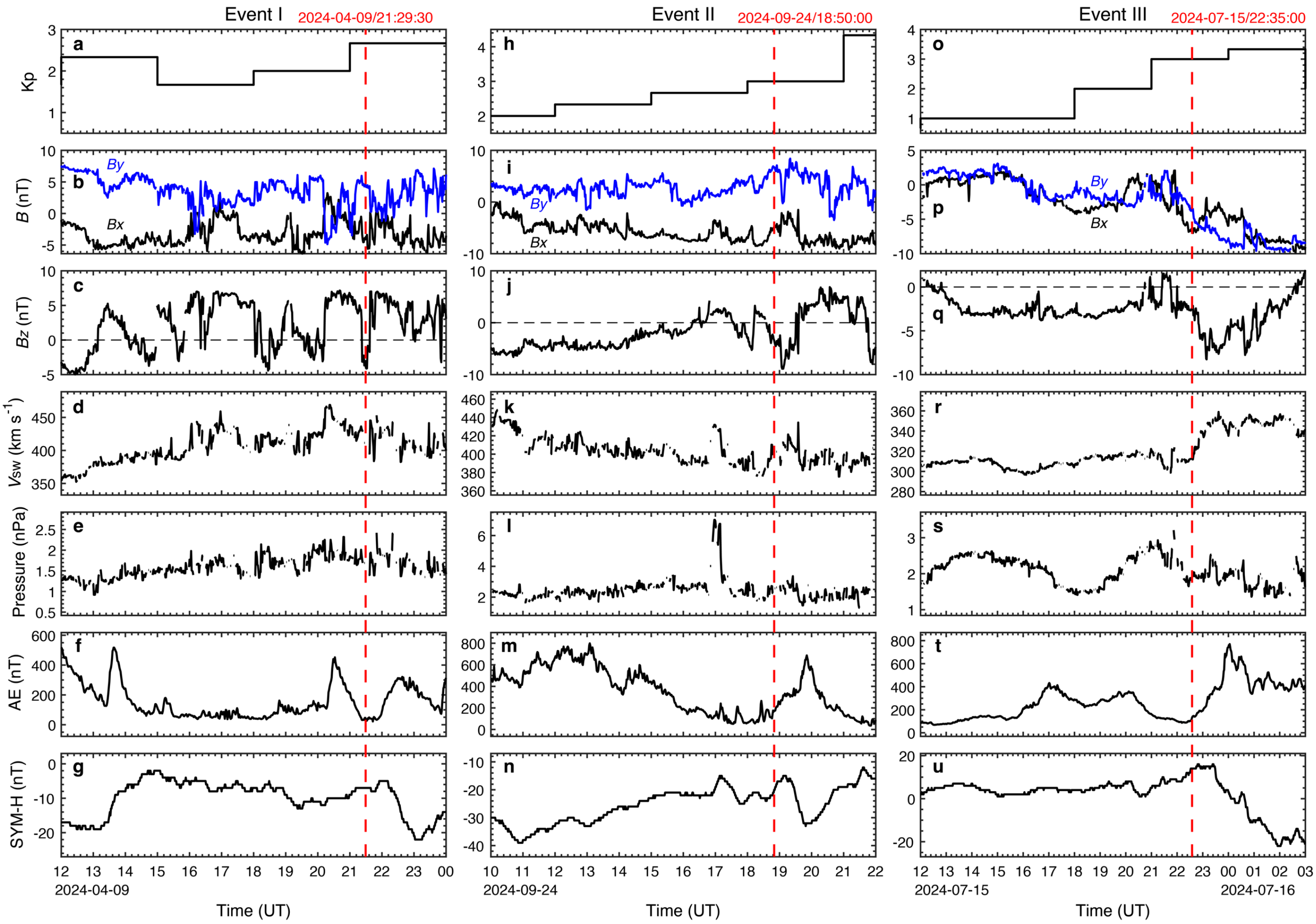

Supplementary Fig. 6 Solar wind and geomagnetic indices during the events analyzed in this study. The left column shows the indices for Event I, including the temporal variations of **a** Kp index, **b** B_x (black) and B_y (blue) components of interplanetary magnetic fields (IMF), **c** IMF B_z, **d** solar wind speed V_{sw}, **e** solar wind dynamic pressure, **f** AE, and **g** SYM-H. The vertical red line marks a characteristic time of observed SAA transient distortions. The middle column **h-n** and right column **o-u** are in the same format as in the left column, except that they display parameters for Event II and III, respectively.

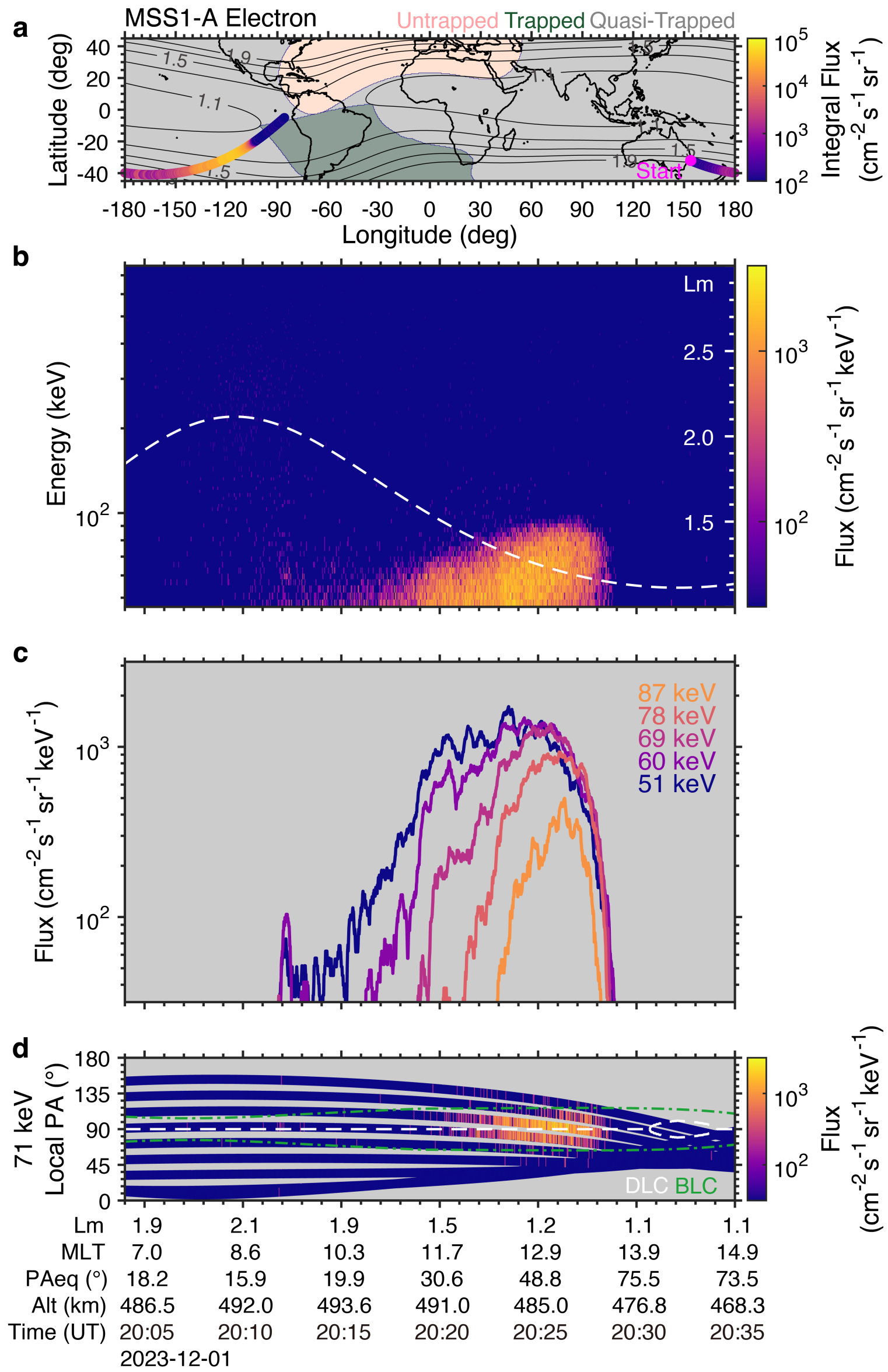

Supplementary Fig. 7 Example of MSS1-A observations for the SAA distortion on the west side. **a** Satellite ground track, with the color representing the energy-integral fluxes of electrons with pitch angle around 90°. The background colormap indicates different trapping regions at the satellite orbital altitude, accompanied by the McIlwain L-parameter contours with an interval of 0.2. **b** Energy-time spectrogram for electrons near 90° pitch angle, with the dashed white line representing L-shell variations. **c** Temporal variations of electron fluxes for several energy channels in panel b. **d** Local pitch-angle distributions for 71-keV electrons, measured by nine detectors. The white dashed and green dash-dotted lines represent the drift loss cone (DLC) and bounce loss cone (BLC), respectively. The ephemeris data listed at the bottom include the McIlwain L-parameter (Lm), magnetic local time (MLT), the equatorial pitch angle (PAeq) of a locally-mirroring electron, and the satellite geodetic altitude (Alt).
