# Supplementary material for: Transient distortions of the South Atlantic Anomaly radiation environments driven by electric fields
Source: Nat Commun. 2026 Jul 3;17:8345. doi: 10.1038/s41467-026-74984-z (PMC13473148; doi:10.1038/s41467-026-74984-z)
Supplement: Supplementary file 2 [file 41467_2026_74984_MOESM2_ESM.pdf]

nature portfolio

Peer Review File

Transient Distortions of the South Atlantic Anomaly Radiation Environments Driven by Electric Fields

Corresponding Author: Dr Xuzhi Zhou

This file contains all reviewer reports in order by version, followed by all author rebuttals in order by version.

Attachments originally included by the reviewers as part of their assessment can be found at the end of this file.

Version 0:

Reviewer comments:

Reviewer #1

(Remarks to the Author)
The manuscript entitled “Transient Distortion of the South Atlantic Anomaly Radiation Environments Driven by Electric Fields” by Yin et al. discusses two events (April 9, 2024 and July 15, 2024) during which “abrupt, short-duration enhancements” (l.40) of energetic (50keV – 200 keV) electron fluxes where observed by the Low-Earth Orbit (LEO) Macau Science Satellite (MSS1-A) in the quasi-trapped region (for L values between 1.8 and 2.3), east of the South Atlantic Anomaly (SAA). In both cases, “zebra stripes” (a certain type of transient variations in particle distributions) were observed shortly after by the other LEO spacecraft of the mission (MSS1-B) in the trapped region (SAA). Using test-particle simulations, it is shown that large-scale electric field perturbations in the inner belt could explain both observed flux enhancements and subsequent zebra stripes. It is concluded that “radiation threats from inner belt particles to LEO spacecraft are not confined to the traditional SAA region but can extend dynamically beyond it.” (l.711-712) Adding that, “this discovery reveals a previously unrecognized variability of the inner belt, providing fresh insights into near-Earth space weather and new considerations for spacecraft protection” (l.713-714).

While the reported observations made by the MSS1 are certainly of interest and worthy of publication, the significance of the results remains to be demonstrated. In this context, it is unclear whether Nature Communications is the appropriate journal to publish this work. The reasons for this evaluation are the following:

1. The manuscript asserts that the observations reveal a “less recognized” (l.45) or “previously unrecognized” (l. 712) variability of the inner belt. Yet, there are multiple studies that have shown electron flux enhancements beyond the SAA at LEO, including for regions and energies similar to what is shown in this manuscript (e.g., Sauvaud et al., 2008, their Figure 2, https://doi.org/10.1029/2008GL033194). For instance, there are many works by A.V. Suvorova et al. that focus on this topic, in particular below L = 1.2 (e.g., https://doi.org/10.1002/2017JA024556 and references therein). In that context, it is unclear what discovery this manuscript presents.

2. The manuscript claims that “these findings […] extend[] the radiation hazards beyond the SAA boundaries”. Yet, this is not proven. Electron fluxes in the SAA correspond to fluxes in the inner belt, covering tens of keV up to MeV energies at times (e.g., Baker et al.. 2007, https://doi.org/10.1029/2007GL031007). In comparison, the transient flux enhancements reported are for 50-200 keV particles. Whether such flux enhancements indeed represent a “radiation hazard” needs to be demonstrated.

3. Furthermore, because the fluxes in the SAA are not similar to the flux enhancements reported observed across the entire energy spectrum, and because they are located away from the SAA, it is questionable whether these events do actually represent “transient distortions of the SAA radiation environment” (l.39). In that context, the title is misleading and would need to be rephrased.

4. The manuscript claims that “the novel observations of transient SAA distortion offer sharper constraints on the properties of transient electric fields in the inner belt”. Yet, this is not proven. The assumed electric fields are represented by ad-hoc formulas (section 4.4), which seem to have been created so that they would yield a satisfying model-observation comparison. That said, there is no proof that these models are the only solutions that would yield a good match between

modeled and observed fluxes. In short, it is not clear how the derived electric field models could be of use in other cases besides the ones presented in this manuscript. It is also unclear how useful they could be to other researchers in the community.

Other comments:

1. Conservation of the second adiabatic invariant is also typically required during radial transport. Yet, this is missing from the discussion (e.g., l.172-176, 348). Furthermore, the spacecraft observe different equatorial pitch angles during their orbits. It would help the reader (and facilitate reproducibility of work) to provide this information when presenting observations (at least for 90 degree particles).

2. An analysis of the solar wind parameters and/or magnetic indices associated with these events could provide useful and complementary information for these case studies.

3. l.50: “to pose significant hazard”. L.336: “we select several electrons”. These awkward phrasings need to be reformulated.

(Remarks on code availability)

Reviewer #2

(Remarks to the Author)
This paper describes observations from two LEO satellites showing sudden increases in precipitating electron flux eastward of the South Atlantic Anomaly (SAA) region. Modelling demonstrates that these events are likely caused by a sudden azimuthal (westward) electric field enhancements which change the orbits and mirror height of the drifting electrons, which may also be modulated by resonance interactions with ULF plasma waves.

This is a well-written and argued paper and I have no substantive concerns. The figures are clear and the results highlight mechanisms which may lead to energetic electron precipitation to low altitudes beyond the usual SAA boundary. I recommend publication subject to the points below.

The authors have not discussed the origin of the electric field spikes which cause these perturbations, nor the solar wind and geomagnetic conditions for the two events examined. The modelling for event I has assumed a relatively large electric field transient. Previous work [ref 24] has shown that the amplitude of such transients correlates with magnetic activity. However, event I occurs on a relatively quiet Kp<=3 day. Event II was preceded by some days of very quiet conditions, with a small increase to Kp~3 triggering the event. The ULF waves are assumed to interact with 17 MeV particles, which seems like a high energy. Although not the thrust of the paper, I would like to see some comment on the cause of the electric field transients, given that they seem to have occurred on otherwise unremarkable days. This is relevant to considerations of potential space weather impacts on LEO satellites.

Minor points:
Abstract: The reference to anthropogenic disturbances is not relevant and should be removed.
Introduction, line 50: "and pose"
line 52: "Re altitude"
Fig. 6 caption: "the right panels (e) - (j)"

(Remarks on code availability)

Reviewer #3

(Remarks to the Author)
I co-reviewed this manuscript with one of the reviewers who provided the listed reports. This is part of the Nature Communications initiative to facilitate training in peer review and to provide appropriate recognition for Early Career Researchers who co-review manuscripts.

(Remarks on code availability)
The code is easily accessible via the links in the manuscript, including an informative README file.
Examining the code functions, I can see excellent commenting of the code, and instructions on how to reproduce the analysis in the article.

This code is excellently archived and is a positive resource for the community, should they intend to reproduce the results of the paper.

Reviewer #4

(Remarks to the Author)
Please see attached review.

(Remarks on code availability)

Version 1:

Reviewer comments:

Reviewer #2

(Remarks to the Author)
I have perused the authors' responses to my comments and those of the other reviewers, and examined the revised manuscript. The authors have made substantial revisions in response to address those comments and concerns. I have no fundamental concern with the paper as a result.

The paper reports new observations supported by modelling which makes some assumptions but seem reasonable. The significance of the paper is in pointing out that energetic electrons can be found beyond the 'traditional' south Atlantic anomaly region due to the effects of transient electric fields. This has important implications for spacecraft operators. Further, more detailed studies including a range of solar wind conditions will clarify the significance of this finding and the modelling but this paper is the first step.

The methodology is clear and reproducible given suitable observations. The figures are complex but well explained, and the supplemental material is very useful.

(Remarks on code availability)

Reviewer #3

(Remarks to the Author)
This manuscript presents observations from the MSS1 mission of transient energetic electron flux enhancements outside the traditional South Atlantic Anomaly (SAA) region, interpreted as distortions of the inner belt electron environment driven by large-scale electric field perturbations. The study combines multi-satellite observations with test-particle simulations to reproduce key features such as short-duration “vertical stripe” enhancements and subsequent zebra stripe structures. The revised manuscript has been substantially improved compared to the original submission, particularly through the inclusion of an additional event, an expanded discussion of potential physical drivers, and a sensitivity analysis of the modelled electric fields. These additions strengthen the overall analysis and demonstrate that the authors have engaged seriously with the previous round of review.

However, in my assessment, several central concerns remain only partially resolved. In particular, (i) the conceptual definition and use of the SAA throughout the manuscript, (ii) the degree to which the timing and properties of the electric field perturbations are uniquely constrained by the observations, and (iii) the level of generality and novelty claimed relative to prior work. As a result, while the observations are interesting and suitable for publication elsewhere, the current manuscript does not yet support the level of generality and interpretive strength expected for Nature Communications. I therefore recommend that the manuscript is not suitable for publication in its current form in this journal.

Major Comments

1. Definition and use of the SAA

The manuscript would benefit from a clearer and more consistent definition of the South Atlantic Anomaly (SAA), and in particular what is meant by its “boundary” in the context of the presented observations.

The SAA is introduced as a region defined primarily by a depression in the geomagnetic field strength, i.e., a magnetic-field-driven feature. However, the analysis throughout the manuscript is based on energetic electron flux measurements at satellite altitude, and the central claims concern “distortions” or “extensions” of the SAA inferred from these flux enhancements, which are attributed to electric (rather than magnetic) field perturbations.

While an observational definition of the SAA in terms of particle accessibility (e.g., mirror altitude relative to spacecraft altitude) is physically reasonable, this definition is not explicitly formalized or consistently applied. As a result, there is an ambiguity between a magnetic definition of the SAA and an operational definition based on particle fluxes.

This ambiguity makes it difficult to assess whether the reported features should be interpreted as true “distortions of the SAA” or as transient modifications of the electron flux distribution outside the nominal SAA region.

I recommend that the authors either:

- clearly define and consistently apply an observational definition of the SAA (with appropriate references), or
- moderate the interpretation to avoid implying a direct distortion of the SAA if the results, instead, reflect transient changes in particle fluxes at low altitudes and latitudes.

2. Timing and uniqueness of the inferred electric field perturbation

A central claim of the manuscript is that the observed vertical-stripe enhancement provides strong constraints on the timing and properties of the driving electric field perturbation.

However, it is not yet clear that the observations uniquely require an electric field occurring immediately prior to the MSS1-A pass. During the interval in which the vertical stripe is observed, the spacecraft samples a changing trajectory in L-shell, magnetic local time, and pitch-angle space. It is therefore possible that the spacecraft intersects a pre-existing drift-shell/longitude structure, rather than observing the immediate formation of the enhancement.

The manuscript uses the subsequent zebra-stripe observations to constrain the timing of the perturbation, but it is also noted that the inferred onset time carries an uncertainty of order hours. Given this uncertainty, the observations alone do not appear to exclude a scenario in which the enhanced population was transported much earlier than 10 minutes and later sampled along the spacecraft trajectory.

The simulations and sensitivity analysis demonstrate that a narrow range of electric field parameters can reproduce the observed features. However, these constraints are inherently model-dependent, and it is not demonstrated that the proposed solution is unique, or that alternative temporal or spatial configurations could not produce similar signatures.

I recommend that the authors:

- more clearly distinguish between constraints derived directly from observations and those inferred through modelling, and
- explicitly address whether alternative scenarios (e.g., earlier transport followed by spatial sampling) can be ruled out.

3. Level of novelty and generality

The manuscript presents compelling case studies of transient flux enhancements and their possible interpretation in terms of electric field perturbations. However, the level of novelty relative to prior observations of flux enhancements outside the SAA remains somewhat interpretational, rather than observational.

Similarly, the manuscript introduces the idea that these features may provide a “diagnostic tool” for constraining electric field properties. While this is an interesting prospect, the current analysis is based on a small number of events and a specific modelling framework. The extent to which this approach can be generalized is not yet demonstrated.

As such, several of the broader claims regarding novelty, generality, and diagnostic capability appear stronger than is supported by the current evidence.

I suggest that the authors moderate these claims and more clearly position the work as a promising but still developing interpretation, rather than a broadly established diagnostic framework.

In summary, this manuscript presents interesting and well-executed observations, and the authors have made meaningful improvements in response to prior reviews. However, key aspects of the interpretation remain insufficiently constrained, and several central claims are stronger than can currently be supported.

(Remarks on code availability)
The code is easily accessible via the links in the manuscript, as expected, as it is virtually unchanged from the previous review round. This includes an informative README
file.

As previous, upon examination of the code functions, I can see good commenting of the code, and
instructions on how to reproduce the analysis.
This code is well archived and is a positive resource for the community, should they
intend to reproduce the results of the paper.

Reviewer #4

(Remarks to the Author)
Please see attached review.

(Remarks on code availability)

Version 2:

Reviewer comments:

Reviewer #3

(Remarks to the Author)
I have reviewed the author responses to my previous review, and their updated manuscript. The authors have provided substantial reassurance and clarifications based on the comments I made, which is very much appreciated. I am satisfied that they have been addressed.

I am happy to now recommend the publication of this manuscript in its present form.

(Remarks on code availability)

Open Access This Peer Review File is licensed under a Creative Commons Attribution 4.0 International License, which permits use, sharing, adaptation, distribution and reproduction in any medium or format, as long as you give appropriate credit to the original author(s) and the source, provide a link to the Creative Commons license, and indicate if changes were made.
In cases where reviewers are anonymous, credit should be given to 'Anonymous Referee' and the source.
The images or other third party material in this Peer Review File are included in the article's Creative Commons license, unless indicated otherwise in a credit line to the material. If material is not included in the article's Creative Commons license and your intended use is not permitted by statutory regulation or exceeds the permitted use, you will need to obtain permission directly from the copyright holder.
To view a copy of this license, visit https://creativecommons.org/licenses/by/4.0/

Response to Reviewers

We are grateful to the reviewers for their constructive comments, which indeed provided us the opportunity to further improve our paper. We have carefully considered the comments and accordingly revised the manuscript. Please find below the responses to the comments, in which the reviewers' comments are shown in blue and our replies are in black.

To Reviewer #1

The manuscript entitled "Transient Distortion of the South Atlantic Anomaly Radiation Environments Driven by Electric Fields" by Yin et al. discusses two events (April 9, 2024 and July 15, 2024) during which "abrupt, short-duration enhancements" (l.40) of energetic (50keV – 200 keV) electron fluxes where observed by the Low-Earth Orbit (LEO) Macau Science Satellite (MSS1-A) in the quasi-trapped region (for L values between 1.8 and 2.3), east of the South Atlantic Anomaly (SAA). In both cases, "zebra stripes" (a certain type of transient variations in particle distributions) were observed shortly after by the other LEO spacecraft of the mission (MSS1-B) in the trapped region (SAA). Using test-particle simulations, it is shown that large-scale electric field perturbations in the inner belt could explain both observed flux enhancements and subsequent zebra stripes. It is concluded that "radiation threats from inner belt particles to LEO spacecraft are not confined to the traditional SAA region but can extend dynamically beyond it." (l.711-712) Adding that, "this discovery reveals a previously unrecognized variability of the inner belt, providing fresh insights into near-Earth space weather and new considerations for spacecraft protection" (l.713-714).

While the reported observations made by the MSS1 are certainly of interest and worthy of publication, the significance of the results remains to be demonstrated. In this context, it is unclear whether Nature Communications is the appropriate journal to publish this work. The reasons for this evaluation are the following:

Thank you for your time and efforts reviewing our manuscript. We appreciate your recognition that the reported observations are "certainly of interest and worthy of publication." We also take your concern regarding the significance of the results and the journal suitability very seriously. To address your concerns, we have performed a major revision of the manuscript. The key improvements include:

1. New evidence of SAA expansion (new Event II): We have added a new event (September 24, 2024) that exhibits a contiguous eastward expansion of the SAA electron environment (see revised Fig. 6 and Section 2.4). This is a crucial addition because it displays another manifestation of transient distortions of the SAA electron environment. It demonstrates that the phenomenon is not merely a transient local anomaly but represents a significant distortion and extension of the SAA boundary driven by electric fields.
2. A generalizable diagnostic method: We have significantly deepened the discussion to

propose that these SAA distortions can serve as a novel and sensitive remote-sensing tool. Unlike the traditional "zebra stripes" which often yield non-unique solutions with large uncertainties, the transient distortions of the SAA electron environment can, in principle, provide much tighter constraints on the onset time, amplitude and duration of the electric field perturbations in the inner magnetosphere. Future statistical work will assess how widely this diagnostic can be applied (see the revised Discussion section).

3. Clarification of radiation hazards: We have expanded the introduction and discussion to explicitly address the practical implications for LEO spacecraft (e.g., surface charging and sensor interference). Our findings reveal that radiation risks extend dynamically beyond the traditional SAA boundaries, necessitating an evaluation of spacecraft protection strategies in LEO.

We believe that with these new results and deeper physical interpretations, the revised manuscript now demonstrates the broad significance and novelty suitable for the readership of *Nature Communications*.

1. The manuscript asserts that the observations reveal a "less recognized" (l.45) or "previously unrecognized" (l. 712) variability of the inner belt. Yet, there are multiple studies that have shown electron flux enhancements beyond the SAA at LEO, including for regions and energies similar to what is shown in this manuscript (e.g., Sauvaud et al., 2008, their Figure 2, https://doi.org/10.1029/2008GL033194). For instance, there are many works by A.V. Suvorova et al. that focus on this topic, in particular below L = 1.2 (e.g., https://doi.org/10.1002/2017JA024556 and references therein). In that context, it is unclear what discovery this manuscript presents.

Thank you for highlighting these important papers. We agree that electron flux enhancements outside the SAA at LEO have been reported previously. In fact, in the original version of our manuscript, we have already cited a few relevant studies attributing such observations to either VLF transmitter waves (Hua et al., 2020; Meredith et al., 2019) or radial electron transport (Selensnik et al., 2016; Lejosne and Mozer, 2020). These mechanisms align with the two papers you mentioned (i.e., Sauvaud et al., 2008; Suvorova, 2017), respectively. However, the phenomenon reported in our study is distinctly different from those described before, both in terms of generation mechanism and observational characteristics.

More specifically, the structures reported in Sauvaud et al. (term as "wisps") are caused by the interaction between electrons and VLF transmitter emissions. This is a pitch-angle scattering process occurring near a series of transmitters (most significantly, the US Navy transmitter NWC in Australia), which leads to the precipitation of energetic electrons. In contrast, the transient distortions of the SAA radiation environment, manifesting as vertical stripes or contiguous expansions as shown in our study, are driven by large-scale electric fields. This is primarily an adiabatic process, during which electrons are accelerated by large-scale electric fields. To conserve the first and second adiabatic invariants, the magnetic field strength at the mirror point must increase, causing the mirror height reduction of the electrons below the satellite altitude.

For the Forbidden Energetic Electron (FEE) events reported in Suvorova (2017), they mainly focus on the presence of electrons at very low L-shells (L<1.2). Therefore, their presence was attributed to the radially-inward electron transport associated with the ExB drift motion. In contrast, our study highlights the immediate modulation of electron mirror heights within the inner belt (L~1.2-2), which enables an eastward (rather than inward) extension of the SAA radiation environments. Moreover, the FEE events often represent an integrated response formed over hours, whereas the transient SAA distortions we observe are an immediate response to the electric field perturbations, providing much stronger constraints on the electric field properties.

In summary, the adiabatic SAA distortions reported in our paper represent a new mechanism that has never been discussed before, and the associated new manifestation of inner belt dynamics (as electron flux enhancements outside the traditional SAA boundary to exhibit a longitude extension) serves as a more direct indicator of transient electric fields than any phenomena studied previously.

We have revised the Abstract (Lines 42-43, 46-47) and Introduction section (Lines 83, 147-149) to reflect this context more clearly. Regarding the two papers you mentioned, Sauvaud et al. (2008) is now cited in the introduction section, and the Suvorova (2017) paper (cited already in the original version) is also kept in the revised manuscript.

2. The manuscript claims that "these findings [···] extend[] the radiation hazards beyond the SAA boundaries". Yet, this is not proven. Electron fluxes in the SAA correspond to fluxes in the inner belt, covering tens of keV up to MeV energies at times (e.g., Baker et al.. 2007, https://doi.org/10.1029/2007GL031007). In comparison, the transient flux enhancements reported are for 50-200 keV particles. Whether such flux enhancements indeed represent a "radiation hazard" needs to be demonstrated.

Thank you for the valuable comments. The electron radiation hazards in the radiation belts indeed cover a wide energy range from tens of keV up to MeV, although we agree that it is important to specify the energy dependence of the hazards. The MeV population has long been associated with deep dielectric charging, whereas at lower energies (tens or a few hundreds of keV), the electrons still constitute a significant radiation hazard and operational risk through three distinct mechanisms below.

The first one is surface and (shallow) internal charging (e.g., Garrett & Whittlesey, 2012, https://doi.org/10.1002/9781118241400). The electrons below 100 keV primarily contribute to surface charging, which can lead to differential charging of spacecraft surfaces and discharge damage. Those electrons above 100 keV can penetrate thin protective layers (like thermal blankets) and lead to shallow internal charging of dielectric materials, which can cause electrostatic discharge close to victim circuits.

The second one is the cumulative total ionizing dose (TID; e.g., de Groh et al, 2018,

https://doi.org/10.1016/B978-0-323-52472-8.00029-0). The ~50-200 keV electrons can deposit most of their energy within the surface materials and sensitive external payloads (like solar arrays), accelerating their degradation. The expansion of the SAA electron environments reported in our work implies an accelerated accumulation of TID for LEO satellites traversing these regions.

The third one is instrument interference. These energetic electrons can act as a significant source of background noise for scientific payloads like X-ray or gamma-ray telescopes (e.g., Aslanyan et al., 2019, https://doi.org/10.1063/1.5120410). The transient extension of SAA-level fluxes into a region theoretically considered "quiet" poses a direct risk of data contamination.

Here, we would also note from a theoretical perspective that the proposed mechanism in our paper is not intrinsically limited to the flux enhancements within the 50-200 keV range. The electric field-induced mirror point reduction is physically capable of acting on higher-energy electrons, provided that the interaction conditions are suitable (though rather demanding), such as a sufficiently large amplitude or an optimal spatio-temporal distribution of the electric field. Specifically, given the rapid drift of relativistic electrons, the electric field perturbation must be pulsed with a large amplitude to ensure a sufficient net energy gain. An ideal case is when the electric field pulse aligns with the electron drift path. Considering that the gradient-curvature drift velocity is approximately proportional to the particle energy (W), the energy gain from the pulsed electric field can be proportional to the particle's initial energy ($\Delta W \propto W$). Consequently, the relative energy change ($\Delta W/W$) and the resulting mirror height reduction (proportional to $\Delta W/W$) can remain significant across a broad energy spectrum under this ideal condition. Moreover, the observability of such effects also depends on the inherent distribution of the inner radiation belt. For example, the flux enhancements in the revised Event II exhibit a continuous spectral transition from inside to outside the SAA, which is related to the spatial distributions of the trapped population. The absence of ~MeV signatures is partially attributed to the low abundance of relativistic electrons observable by MSS1 satellites. To remain strictly data-driven, however, we have confined our manuscript revisions to the hazards explicitly supported by our MSS1 observations (i.e., the ~50-200 keV population).

We have accordingly revised the Introduction (Lines 67-73) to detail the specific risks from the ~50–200 keV electron population. We also clarified the wording in the Abstract (Line 47), from "radiation hazards" to "electron radiation risks".

3. Furthermore, because the fluxes in the SAA are not similar to the flux enhancements reported observed across the entire energy spectrum, and because they are located away from the SAA, it is questionable whether these events do actually represent "transient distortions of the SAA radiation environment" (l.39). In that context, the title is misleading and would need to be rephrased.

We agree that in our observations (Fig. 2), the vertical stripe was separated from the

traditional SAA region, which may appear different from "transient distortions of the SAA radiation environments". However, this apparent inconsistency can be attributed to the timing of the perturbed electric field, which appears when the MSS1-A satellite is about to exit the SAA. In this case, the resulting SAA distortion (that is, the mirror-height reduction of the stably-trapped electrons) can only be manifested in MSS1-A observations as electron flux enhancements detached from the traditional SAA region. In other words, if the electric field perturbations appear earlier, one could expect a more seamless expansion of the SAA.

To further illustrate this point, we have added another event displaying contiguous expansion of the SAA radiation environments, as the revised Event II in the main text (Section 2.4). As shown in the revised Fig. 6, the enhanced electron fluxes extend seamlessly outward from the SAA region. Unlike the detached structure (the "vertical stripe") in Event I, Event II displays an overall eastward expansion of the SAA eastern boundary. Our simulations (Supplementary Fig. 3) confirm that this continuous expansion is driven by the same mechanism. This new evidence supports our scenario that the observed events are not independent phenomena located "away from the SAA," but are dynamic extensions of the SAA boundary. Depending on the electric field properties, this distortion can manifest as either "detached vertical stripes" (Event I) or "contiguous expansions" (Event II). In fact, Event III may also be interpreted as "contiguous expansions" if the ULF waves were not present.

We have also accordingly revised the abstract, and added a new section 2.4 (Lines 484-542) and a new figure (Figure 6) to make this point clear.

4. The manuscript claims that "the novel observations of transient SAA distortion offer sharper constraints on the properties of transient electric fields in the inner belt". Yet, this is not proven. The assumed electric fields are represented by ad-hoc formulas (section 4.4), which seem to have been created so that they would yield a satisfying model-observation comparison. That said, there is no proof that these models are the only solutions that would yield a good match between modeled and observed fluxes. In short, it is not clear how the derived electric field models could be of use in other cases besides the ones presented in this manuscript. It is also unclear how useful they could be to other researchers in the community.

You make an important point that the specific constraints on electric field properties should have been articulated more clearly and quantitatively. To achieve this, we have performed a comprehensive sensitivity analysis (presented in Supplementary Fig. 4), to show that the vertical stripe in Event I is extremely sensitive to electric field parameters.

In this analysis, we have tested the sensitivity of simulation results to three parameters: electric field onset time, amplitude, and duration, represented by the time shift, amplitude ratio, and duration ratio to the settings used in the main text (where time shift=0 and ratios=1). The vertical stripe observation imposes constraints on parameters such that test electrons before and after the stripe must precipitate into the 100-km boundary during their backward tracing, while test electrons within the stripe must have a minimum mirror height above 100 km. The parameter sets satisfying these requirements are indicated by magenta dots in Supplementary

Fig. 4, which indicate that the feasible domain of electric field parameters is extremely small.

First, the sensitivity results regarding the time shifts (panels a-f) indicate the onset time of the perturbed electric field with a precision of minutes. An earlier onset of the electric field would allow electrons ahead of the vertical stripe to interact with the field for a longer duration. This would result in the extension of the stripe's leading edge, even connecting it to the main SAA body to form a contiguous expansion (as seen in the newly-added Event II). Conversely, a later appearance would be insufficient to accelerate electrons to produce the observed flux enhancements within the vertical stripe.

Second, the constraints on the coupled duration and amplitude (panels g-i) show the feasible ratios can vary from (0.7, 1.4) to (1.1, 0.9), all corresponding to a pulsed electric field of several mV/m and duration in minutes. An electric field with larger amplitude and/or longer duration would cause a stronger distortion of the SAA region, again resulting in an enlargement of the observed vertical stripe, even into a contiguous expansion. On the other hand, an electric field with smaller amplitude and/or shorter duration would be insufficient to reduce the mirror point of energetic electrons to the satellite altitude to form the observed flux enhancements.

Moreover, the energy range of the observed vertical stripe also imposes constraints on the properties of electric field perturbations. As shown in the bottom two rows in Supplementary Fig. 4, a combination of parameters, namely an earlier onset time (time shift of -5 minutes), weaker amplitude, and longer duration, can satisfy the constraints for ~71 keV electrons (panels j-l). However, for higher-energy electrons with faster drift motion, the flux enhancements within the vertical stripe require acceleration large enough over a shorter time period, necessitating a sufficiently strong electric field (panels m-o). Therefore, the energy range of the observed vertical stripe further narrows the feasible parameter space (note the feasible solutions for two energies do no overlap).

In summary, although the ad-hoc formula is a simplified tool, the simulation results prove that any electric field model capable of explaining these observations must possess a comparable amplitude (several mV/m), duration (~10 minutes), and similar onset time within a short window of minutes. This is useful to the community because it provides significantly strong constraints on the electric field perturbations, whereas previous methods (like derivation from zebra stripes) suffer from large uncertainties on the derived onset time. They provide even less information for the electric-field duration and amplitude.

We have added the sensitivity analysis results in Supplementary Fig. 4 and have accordingly revised the Discussion section in Lines 734-750 to quantitatively describe these constraints, and in Lines 798-805 to state the applicability of our findings to the community.

Other comments:

1. Conservation of the second adiabatic invariant is also typically required during radial transport. Yet, this is missing from the discussion (e.g., l.172-176, 348). Furthermore, the

spacecraft observe different equatorial pitch angles during their orbits. It would help the reader (and facilitate reproducibility of work) to provide this information when presenting observations (at least for 90 degree particles).

Thank you for pointing out these omissions. We have accordingly modified the text in the Lines 242 and 446, to explicitly state that the electron motion is governed by the conservation of both the first and second adiabatic invariants.

We also agree that providing the equatorial pitch angle context is crucial for reproducibility. We have updated the observational plots (Figures 2, 3, 6, 7, and supplementary Fig. 6) to include the equatorial pitch angle that corresponds to the locally mirroring electrons (see the ephemeris tables below the corresponding panels).

2. An analysis of the solar wind parameters and/or magnetic indices associated with these events could provide useful and complementary information for these case studies.

We have followed this recommendation to plot the variations of solar wind parameters and magnetic indices for all events in Supplementary Figure 5. We have also expanded the Discussion (Lines 763-774) to interpret these signatures and discuss the potential mechanisms for these electric field perturbations.

For Events II and III, the observations coincide with a period of rising AE index, consistent with the scenario of electric field penetration during the substorm onset phase. Interestingly, Event I occurs during a relatively quiet substorm condition. However, the solar wind data reveals a distinct northward-to-southward turning of the IMF Bz prior to the event, which suggests the electric field perturbation for Event I may be associated with the solar wind disturbances. The relative contributions of solar wind disturbances and substorm activities to electric field perturbations in the inner radiation belt require further investigations.

3. l.50: "to pose significant hazard". L.336: "we select several electrons". These awkward phrasings need to be reformulated.

Thank you for pointing out these awkward phrasings. We have rephrased these sentences to improve clarity and formal tone:

1. Original: "…to pose significant hazards…"
   Revised (Line 55): "…posing significant hazards…"
2. Original: "…we select several electrons…"
   Revised (Lines 358-359): "…we examine the trajectories of representative test electrons encountering MSS1-A…"

Once again, thank you for the time and effort spent in evaluating our work. The constructive comments regarding the significance of the findings, the distinction from previous studies, and the physical constraints on the electric field have led to a much stronger and more

rigorous manuscript. We hope that our modifications, especially the newly-added event and the sensitivity analysis, have satisfactorily addressed your concerns.

To Reviewer #2

This paper describes observations from two LEO satellites showing sudden increases in precipitating electron flux eastward of the South Atlantic Anomaly (SAA) region. Modelling demonstrates that these events are likely caused by a sudden azimuthal (westward) electric field enhancements which change the orbits and mirror height of the drifting electrons, which may also be modulated by resonance interactions with ULF plasma waves.

This is a well-written and argued paper and I have no substantive concerns. The figures are clear and the results highlight mechanisms which may lead to energetic electron precipitation to low altitudes beyond the usual SAA boundary. I recommend publication subject to the points below.

Thank you for your kind support. We have followed your comments to revise the manuscript, and we hope that your concerns are satisfactorily addressed.

The authors have not discussed the origin of the electric field spikes which cause these perturbations, nor the solar wind and geomagnetic conditions for the two events examined. The modelling for event I has assumed a relatively large electric field transient. Previous work [ref 24] has shown that the amplitude of such transients correlates with magnetic activity. However, event I occurs on a relatively quiet Kp<=3 day. Event II was preceded by some days of very quiet conditions, with a small increase to Kp~3 triggering the event. The ULF waves are assumed to interact with 17 MeV particles, which seems like a high energy. Although not the thrust of the paper, I would like to see some comment on the cause of the electric field transients, given that they seem to have occurred on otherwise unremarkable days. This is relevant to considerations of potential space weather impacts on LEO satellites.

Thank you for the constructive suggestion. We agree that the inclusion of solar wind and geomagnetic indices is beneficial for our understanding of such transient processes and their space weather impacts. We have added Supplementary Fig. 5 to show the variations of solar wind parameters and magnetic indices for the events analyzed in this study.

Please note that we have added a new event (September 24, 2024) as Event II in the revised manuscript, which shows an eastward expansion of SAA electron environment. Consequently, the recurrent stripe event (July 15, 2024) referred to as "Event II" in your review is now labeled as Event III.

For Event I, it indeed occurs on a relatively quiet day. The Kp index is relatively small and AE index indicates no significant substorm activity. However, as shown in the Supplementary Fig. 5, there is a distinct IMF Bz reversal from northward to southward prior to the event. As

suggested by previous studies (e.g., Huang, 2019, https://doi.org/10.1029/2019JA026793), such reversals may drive penetration electric fields into low latitudes. The duration of this Bz reversal (approximately 10 minutes) aligns well with the constraints on the electric fields from our analysis of the particle observations, which makes it a plausible driver of the electric field perturbations.

For Events II and III, they occur when the Kp index gradually increases. The AE indices are also about to rise, indicating they are in an early stage of a substorm activity. These events are also accompanied by a southward interplanetary magnetic field (IMF). Therefore, these events are likely associated with substorm activity, which agrees with previous studies suggesting the correlation between the substorms and electric field perturbations in the inner radiation belt (e.g., Lejosne & Mozer, 2020, https://doi.org/10.1029/2020JA027889). On the other hand, the potential role of the southward IMF may need further investigation. As proposed in our paper, the distortions of the SAA eastern boundary can be utilized to constrain the properties of electric field perturbations. Building upon this concept, we are planning to conduct statistical analysis to further investigate the relationship between electric field properties and various solar wind and geomagnetic indices, thereby gaining deeper insights into the driving mechanisms of these electric field perturbations.

For the ULF waves in Event III, you are right that the drift resonance energy is very high. This property indicates a small azimuthal wavenumber, which reduces the probability of an internal wave driver. Therefore, it is likely that they are driven externally, either by Kelvin-Helmholtz instability or variations of solar wind dynamic pressure. Unfortunately, there are no satellites located near the magnetospheric flank region at this time, and it is difficult to make a concrete conclusion on the occurrence of K-H instability. On the other hand, we note a rapid decrease in solar wind dynamic pressure prior to the event, although the overall magnitude of the decrease is not significant (~1.5 nPa within ~30 min). This may be a trigger of the ULF waves. Obviously, further investigations are required for more comprehensive understanding of the ULF waves in this low L-shell region.

In addition to the Supplementary Fig. 5, we have also revised the Discussion section (Lines 763-774) to discuss the potential driving mechanisms for these electric field perturbations, hoping to provide further insights for related research and space weather applications.

Minor points:
Abstract: The reference to anthropogenic disturbances is not relevant and should be removed.
Introduction, line 50: "and pose"
line 52: "Re altitude"
Fig. 6 caption: "the right panels (e) - (j)"

Thank you for pointing them out. We have accordingly revised the text in the manuscript.

To Reviewer #3

I co-reviewed this manuscript with one of the reviewers who provided the listed reports. This is part of the Nature Communications initiative to facilitate training in peer review and to provide appropriate recognition for Early Career Researchers who co-review manuscripts.

Reviewer #3 (Remarks on code availability):

The code is easily accessible via the links in the manuscript, including an informative README file. Examining the code functions, I can see excellent commenting of the code, and instructions on how to reproduce the analysis in the article.

This code is excellently archived and is a positive resource for the community, should they intend to reproduce the results of the paper.

Thank you for your positive evaluation and for your time taken in thoroughly examining our code and documentation. We are committed to the principles of open science and reproducibility, and we are delighted to know that our efforts in archiving and documenting the code are recognized as a valuable resource for the community.

In line with this commitment, we have updated the code repository (and the associated DOI) to include the simulation scripts for the newly added Event II (September 24, 2024). We have also included the source code for the Geopack interface to further enhance cross-platform reproducibility. This ensures that the new results presented in the revised manuscript are fully accessible and reproducible.

To Reviewer #4

Dear Editor,

I reviewed the article entitled "Transient Distortions of the South Atlantic Anomaly Radiation Environments Driven by Electric Fields" written by Ze-Fan Yin and co-authors.

The authors use the measurements from both satellites of the Macau Science Satellite mission, MSS1 and MSS2, which orbit at ~450-500 km, with which they report what they called *"transient distortions of the South Atlantic Anomaly (SAA) radiation environment"*. This *"transient distortions"* can be seen as additional patch of electron radiation in the vicinity of the SAA region, where radiation is the highest due to the lower values of the Earth magnetic field. One impacting conclusion of the authors is that *"radiation threats from inner belt particles to LEO spacecraft are not confined to the traditional SAA region but can extend dynamically beyond it."*.

The discovery the author would like to publish in Nature Communications is the observation and explanation of these additional radiation regions by large-scale westward electric field perturbations. The latter cause electron acceleration and change the mirror point of the electrons, which remain more trapped, forming additional radiation patches. The authors add that these electric field perturbations are influenced and modulated by ultra-low-frequency waves. There is a difficulty which the authors note in their introduction which is the current lack of knowledge of the amplitude of transient electric fields in the ionosphere and near-Earth space. We will see below that the authors do not succeed at well justifying their choice for the values and shape of the transient electric fields, which undermines greatly their article and its impact. The detection of the vertical high flux region in the vicinity of the SAA, associated by the authors to some electric field, is made by MMS1 while the second satellite, MSS2, helps to identify the longer-term claimed effect on the SAA electron flux, which should produce some structuration of the radiation flux (called zebra stripes), indeed observed. I find several major lacks or questions which should prevent publication in Nature Communications.

Thank you for the thorough assessment and for summarizing the core contributions of our work regarding the dynamic extension of radiation threats. We appreciate your constructive comments, particularly regarding the need for a more rigorous justification of our analysis and the demonstration of the phenomenon's significance. To directly address your concerns about the "major lacks" and "limited demonstration," we have performed a major revision of the manuscript with two crucial additions:

1. New evidence of contiguous SAA expansion: We have added a new event (September 24, 2024) in the main text as the new Event II. Unlike the "separated patch" in Event I, this event exhibits a seamless eastward expansion of the SAA electron environment (see revised Fig. 6 and section 2.4). It directly addresses your concern about the nature of these distortions, confirming that the observed features are not coincidental occurrences caused by satellite orbit variations or magnetic field model artifacts, but rather represent the dynamic distortions of the SAA electron environment itself.
2. Rigorous parameter justification: We have added a comprehensive sensitivity analysis (Supplementary Fig. 4). This analysis quantitatively demonstrates that the "vertical stripe" morphology imposes strict constraints on the electric field (similar onset time as the settings in the main text, amplitude of several mV/m, duration of ~10 minutes). This proves that our parameter choice is not arbitrary but is a probable solution under observational constraints.

We believe these additions provide the solid demonstration required for *Nature Communications*. Below are our point-by-point responses.

From the map of Figure 1a, the radiation patch is indeed separated from the main radiation region. The patch (i.e the vertical stripe) is located east of the tip of Africa at longitude close to Madagascar (see below more comments on the location). This is interesting. However, I note, first the zebra stripe structures were also measured in the first place by MSS1 and,

second, MSS2 has a different trajectory travelling within a different region of the SAA. Figure 3 shows the MSS2 data in the region 7h later, while this spacecraft has a 94 mins orbit, meaning 4 orbits separate the observations and we do not know what the spacecraft did measure in between the two measurements.

This is a very good question. Indeed, we should have presented the observations from MSS1-A and -B during the 7h-interval between the occurrences of vertical stripe and zebra stripes. These observations and associated analysis are now given in the Supplementary Figs. 1 and 2, in which only the data in the southern hemisphere are shown since the flux levels are very low in the northern hemisphere. We can also find that the enhanced fluxes, as expected, are concentrated within the SAA region.

From this figure, one may find the presence of zebra stripes each time the satellites traverse the SAA region. However, as demonstrated in many previous papers (e.g., Lejosne & Mozer, 2020, https://doi.org/10.1029/2020JA027889), the zebra stripes are formed rather gradually, with discernible features starting to appear several hours after the electric field perturbations. In other words, the zebra stripes in place before (as shown in Fig. 2b) and a few hours after the vertical stripe (like those in Supplementary Figs. 1a, 1b and 1c) must be attributed to the perturbations several hours earlier. As time goes on, the new series of zebra stripes (formed by the perturbed electric field that leads to the vertical stripe) would gradually take over. That is why we use the MSS1-B observations near 0450 UT (~7.5 hours after the vertical stripe) to infer the perturbation time. To examine the accuracy of the inferred time, we have also used the zebra stripe observations displayed in Supplementary Figs. 1 and 2 to infer the perturbation time independently. The results indicate two separated perturbations, occurring near 1210 UT and 2130 UT, respectively. It is the first one (near 1210 UT) that contributes to the zebra stripes in place before the vertical stripe occurs, and the second one near 2130 UT that leads to the formation of the vertical stripe in Fig. 2 and the zebra stripes in Fig. 3. These results are consistent with the analysis in the main text.

Besides, MMS1 and MSS2 altitude orbits are different by 50 km, which is crucial here according to the claim made in Figure 1. Altitude is not mentioned in the text or plotted in the figure. The role it can have is yet unclear. The authors discuss (cf. Figure 1 and associated text) an effect that is associated to variations within *"a few of hundreds of kilometers"*, which is in the range of the 50 km of variation of the orbit of MMS2. The *"few hundreds of kilometers"* is ~200 km reading Figure 1a and 100 km according to Figure 5c, 5i, 5o. This is in the range of the s/c altitude variation. The authors discuss that electrons cannot anymore *"access the spacecraft at lower altitudes"*. Maybe it is just the s/c since the s/c altitude has changed. This is not discussed or accounted for.

Thank you for carefully checking the orbital configurations and for raising the critical question regarding the altitude effect. We certainly agree that the electron fluxes depend on altitude, with the region of enhanced fluxes being smaller at lower altitudes. We have updated all observational figures to include the spacecraft altitudes in the ephemeris labels.

However, the observation of the vertical stripe cannot be attributed to altitude variations. As you can see in the revised Figure 2, the orbital altitude of MSS1-A gradually decreases during this event, which would indicate a shrinking region of enhanced electron fluxes. This is not consistent with the observations of a vertical stripe outside the traditional SAA boundary. Moreover, when we calculate the SAA extents (defined by finite drift loss cone different from 90 degrees, see the white dashed line in Fig. 2d), the spacecraft altitudes have already been taken into account. Therefore, the vertical stripe must represent a real distortion of the SAA radiation environment. This issue has been clarified in Lines 231-236 in the revised paper.

Also, we would clarify that the characteristic electron precipitation altitude (below which atmospheric collision becomes so strong that energetic electrons can be considered lost) is 100 km, which is used consistently in most previous studies as well as in our paper. In fact, the purple surface in Figure 1a is defined based on this scenario (rather than using 200 km as you may think), by ensuring that the electrons mirroring at the purple surface would descend to 100 km in the absence of electric field perturbations as they drift to the SAA center with minimum magnetic field. In other words, the purple surface is tangent to the 100-km plane to mark the boundary between the mirror points of the stably-trapped electrons and those within the drift loss cone. Therefore, a LEO satellite can only expect to observe the enhanced electron fluxes within the time interval between its orbital intersections with the purple surface. In our study, the spacecraft altitude variations have already been accounted for in determining the expected region of enhanced electron fluxes, and therefore, there is hardly any ambiguity in association with the spacecraft altitudes.

We understand that one may be confused by the term "to descend towards altitudes of a few hundred kilometers", since "a few hundred kilometers" appears to be a broad altitude range. But this term refers to the altitudes where trapped electrons can access (and therefore can be observed should there be a spacecraft), rather than the mirror points or the precipitation boundary of these electrons. In the revised manuscript, we have clarified this issue in Lines 64-65, and have rewritten the caption of Figure 1 for a clearer description.

The simulated process by which the spectrograms change from Figure 4a-b to Figure 4c-d is not described in the article. We only read *"Figures 4c-4d show the simulation results at MSS1-B locations several hours afterwards, which reproduce the observations of zebra stripes for locally mirroring electrons and confined pitch-angle distributions outside the local DLC (compared to observations in Fig. 3)."* This is not an analysis. This is a statement without justification. Between both states, there are 7 hours which are spent and we do not see or read about what is happening during that time (from both observations and simulations point of views). This is intriguing since the claimed process of electric field increase is just 9 minutes long. Also the flux measurements of MSS1 et MSS2 are not made in the same region in (Lat, Long) while I believe the simulation from Figure 4a-b to Figure 4c-d is likely made. Not only both s/c do not have the same altitude but also not the same trajectory, with the second not passing through the region of the vertical stripes. This should be more discussed and justified. Why not looking at MSS1 7 or more hours later, then, in the same place? It is unclear what MSS-1 measures over a few orbits: are the vertical stripes gone in the next orbit? The authors

should provide snapshots of the electron flux for each pass in the Supp Mat. Same comment holds for MSS2.

First of all, we clarify that the simulation results in Figures 4a-4b and 4c-4d represent the virtual observations along MSS1-A and MSS1-B trajectories, respectively. This is to ensure that direct comparison can be made between observations and simulations. Sorry for the confusion one may encounter when reading our original manuscript, and we have made proper revisions (see Lines 353-357 and the caption of Figure 4) to make sure that such information is delivered clearly.

On the other hand, we note that the location difference between MSS1-A and -B does not affect our conclusion. This is because, for zebra-stripe observations (from MSS1-B), it is the time elapsed from the electric field perturbations that determines the inter-stripe separations in the drift-frequency space (see Liu et al., 2016 or Lejosne & Moser, 2020 for interpretations of zebra-stripe patterns). As long as the zebra stripes can be observed, this property does not depend on the spacecraft location (other than the dependence of electron drift frequency on location, which has been inherently considered as the observational pattern is transformed towards the drift-frequency space). In other words, the zebra-stripe observations at any location (including at MSS1-B, as is done in our paper) can be used to estimate the perturbation onset time.

Also, we have followed your suggestion to present, in Supplementary Figures 1 and 2, the zebra-stripe observations and associated analysis from MSS1-A and -B satellites during the 7-hour interval in between the observations shown in Figures 2 and 3. The estimation of perturbation onset based on these observations, as discussed in our response to your previous comments, is consistent with the one derived in our paper. We have also accordingly revised the text in Lines 255-258 and 327-328 to clarify this point.

About the method, the authors employ a "backward-tracing simulations of test electrons launched from the spacecraft location to reproduce the observational characteristics", which is central to the demonstration. The main equations are given in the Methods section. I find the method quite old. It is mentioned that Liouville's equation are also solved, no detail given. This whole numerical code has no references, no test cases, and has not been used for other publication. This is not fine to me for Nature Communications. The numerical method is not enough supported.

Thank you for the comments. The simulation approach we employed may indeed be "quite old", but we respectfully disagree with your assessments that the method is "not enough supported" or "not been used for other publication".

There have been numerous studies based on similar approaches (backward-tracing Liouville simulation, see detailed descriptions of test-particle simulation techniques in Marchand, 2010, https://doi.org/10.4208/cicp.201009.280110a). The backward-tracing Liouville approach is introduced in their Section 3.4, which states that the major strength of this method is the

"ability to produce detailed distribution functions at precise locations without statistical sampling errors". Therefore, it is particularly suited for our study in the simulation of electron distributions at specific locations along spacecraft trajectories (those displayed in Fig. 4). Examples of other papers utilizing this approach in the radiation belt physics include Ukhorskiy et al. (Nature, 2014, https://doi.org/10.1038/nature13046) and Lejosne & Mozer (GRL, 2020, https://doi.org/10.1029/2020GL088564), among many others.

Regarding your comments that "Liouville's equation are also solved", we clarify that we do not directly solve the Liouville PDE equation. Instead, we utilize the core principle from Liouville's theorem that the phase-space density is conserved along particle trajectories in the absence of dissipative processes like collisions. In our backward-tracing simulation, we obtain the particle phase-space density at the spacecraft position based on the particle's state before electric field perturbations. This is the standard procedure as described in Marchand (2010) and applied in many previous papers, which ensures that our results are physically rigorous and directly comparable to the body of literature. To avoid confusion, we have added a clearer explanation of this procedure in Section 4.3 (Lines 896-898), together with a citation to Marchand (2010) in the main text.

We also wish to highlight that, as noted by Reviewer 3, the simulation code is fully open-sourced, well-documented, and archived with a DOI, ensuring full reproducibility.

In their method, the authors do not use a full and accurate magnetic field model such as the IGRF model, which they state and justify. They use instead (for simplicity) an eccentric dipole. They do not tilt the magnetic field model either for unknown reason. Because of their approximation, they write that their results *"should be interpreted as robust for large-scale signatures, but not as precise representations of fine-scale field structure"*. I do not think that it is true. How can they assess this is true? They are dealing with fine-scale effects of 100 km and authorize themselves rough approximations, which matter greatly for the definition of the SAA. The SAA is defined by lower magnetic field. If one looks at the low magnetic field region in the SAA at 100 km of altitude, the edge of the SAA as modeled by an eccentric tilted dipole is roughly at the tip of Africa (roughly corresponding with the region plotted as the trapped region in Figure 2 or 3). If one does now look at the IGRF model, the edge of the SAA reaches now the longitude of Madagascar, exactly where the authors find their additional patch. There is an abrupt change in magnetic field in the eastern tip of Africa, not captured by an eccentric tilted dipole. The whole south-eastern tip of Africa falls in the main SAA with the IGRF and not with an eccentric tilted dipole. This means the vertical stripe could simply still be in the SAA, contrarily to what is claimed by the authors. The detail effect of the magnetic field matters a lot in this region. Again, the method here seems insufficient and possibility misleads the authors in their conclusions.

We understand the concern that an inaccurate magnetic field model could lead to a misinterpretation of the "vertical stripe" as an artifact of the SAA edge definition. Below, we clarify two critical points regarding our methodology: (1) how the observational location is identified, and (2) how the simulation model is constructed and validated.

First and foremost, our identification of the "vertical stripe" as being outside the SAA is based on in-situ observations processed with the full IGRF-14 model. The eccentric dipole model is not involved in observational data processing. As shown by the white dashed lines in Figure 2d, we calculate the local drift loss cone (DLC) angle using IGRF model along the satellite orbit. This is the key parameter that allows us to determine whether the satellite is inside or outside the SAA region. When DLC<90°, the satellite is inside the SAA region (in which the stably-trapped particles are expected to be present). When DLC=90°, the satellite exits the SAA and enters the quasi-trapping region. According to Figure 2d, the "vertical stripe" appears ~4 min after the IGRF-calculated DLC becomes 90°. This confirms that, according to the standard IGRF model, the event is located east of the stably-trapping boundary. In addition, we have shown the IGRF-based SAA region (stably-trapping region) near the satellite orbital altitude in Figure 2a as the yellow part of the background, together with an imposed satellite track. It also reveals that the vertical stripe, represented by the small red part along the satellite track, is located outside of the SAA region.

Regarding the eccentric dipole model, as noted in the Method Section 4.3 (see revised text in Lines 856-867), it does include a tilt angle (~9.24°) given by the Gauss coefficients of the standard IGRF-14 model (Fraser-Smith, 1987). To capture the SAA anomaly, we determine the optimal displacement of the dipole center from Earth's center, by minimizing the relative magnetic field difference at 100 km altitude between the eccentric dipole field and full IGRF model in the vicinity of the south Atlantic Ocean (within a latitude range of [−45°, −10°] and a longitude range of [−100°, 150°]). This procedure provides a good agreement between the variations of drift loss cone (DLC) along the satellite orbit in the eccentric dipole field and in the full IGRF model (see the comparison below). Especially, the time when the satellite exits the SAA eastern boundary (when DLC becomes 90°) is estimated rather accurately (with difference within 1 min). We have also revised text in Lines 861-864 to make this point clear.

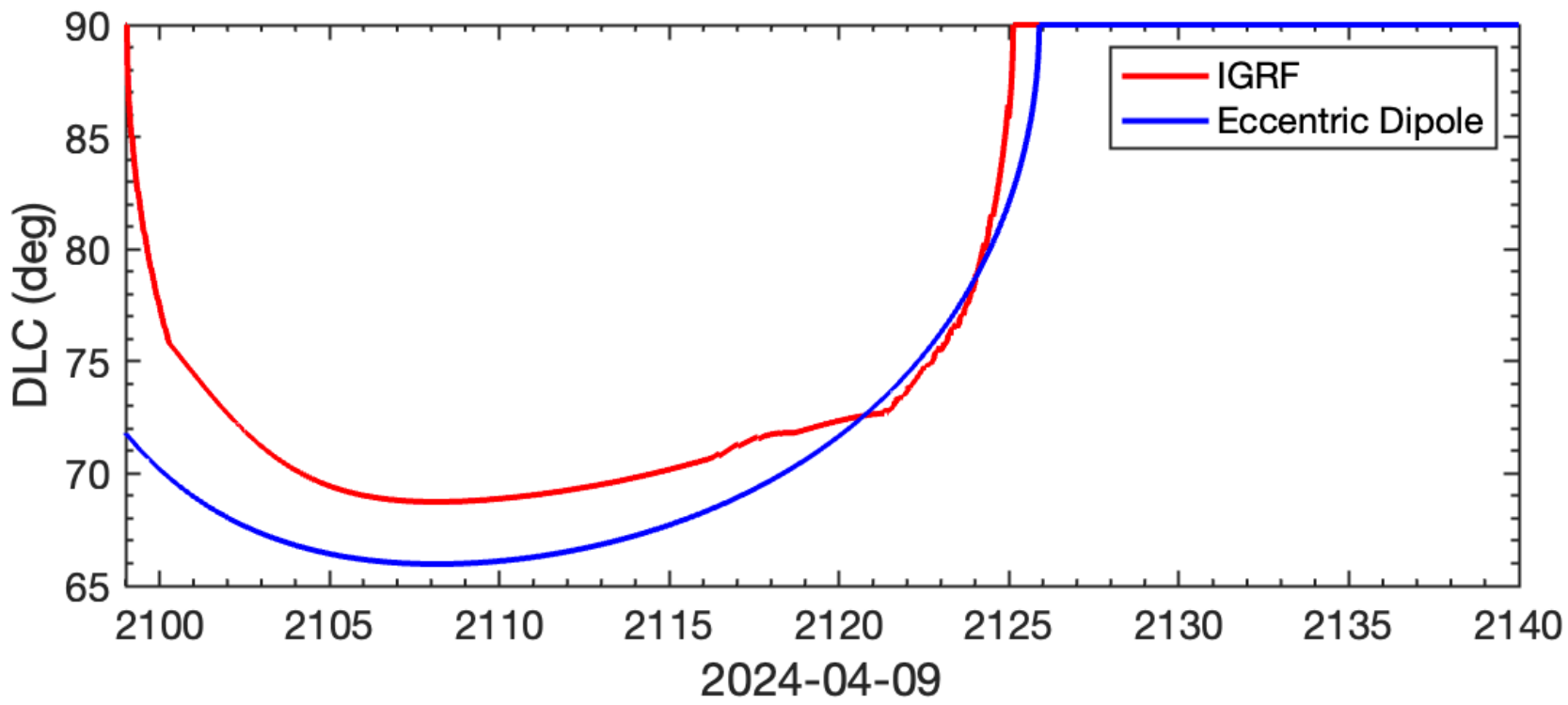

We also show below the magnetic field strengths at 100 km altitude in the IGRF model and in the eccentric dipole model we used, together with a comparison between the SAA eastern boundary at satellite orbital height in both field models. It shows that our eccentric dipole model captures the magnetic field variations near Madagascar, and the SAA eastern boundary in the eccentric dipole field (red dashed curves) matches well with the boundary in the IGRF model (with the yellow background representing the SAA region determined based on IGRF) around the satellite orbit height. Therefore, our optimized model ensures that the simulation

results are physically robust and not artifacts of a “misplaced” SAA edge.

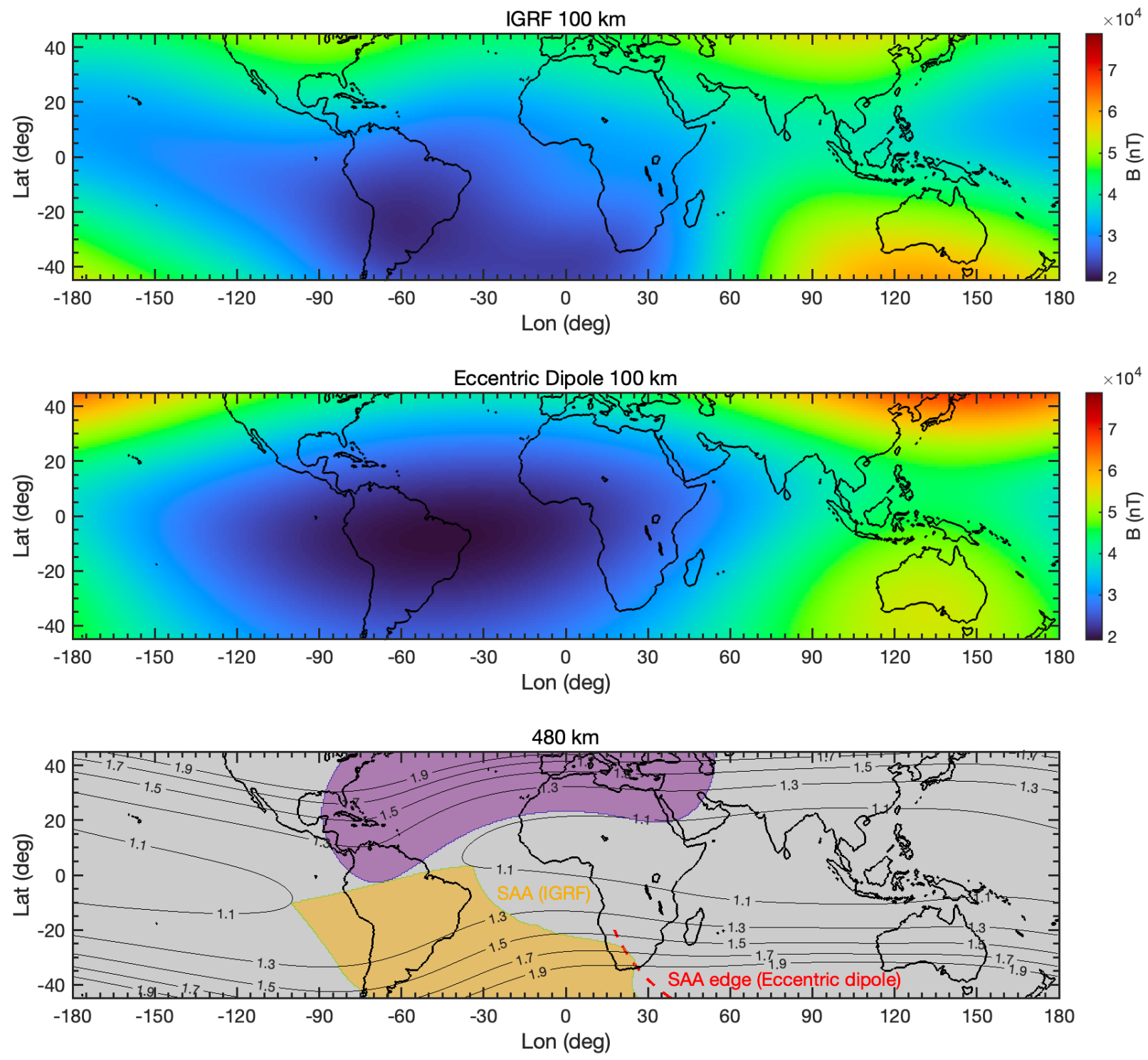

Throughout the article, the authors are careful with not overstating their results and balancing them, which I appreciated reading. However, there is a loss of impact, which is actually supported by an incomplete method. They write *“the recurrent stripes are likely caused by periodic electric field perturbations ……”.* The point of the whole article would be to demonstrate that effect, with a demonstration that is acknowledged as not done and indeed not well supported. The authors also write *“Moreover, since the three vertical stripes exhibit a periodicity of ~175 seconds (falling within the ULF range), it is natural to speculate that the electric field perturbations are carried, at least partially, by ULF waves.”.* I think nobody wants speculations in Nature Communications, in particular regarding a main claim.

Thank you for the comments, and we agree that the original phrasing (“likely caused by”, “speculate”) weakened the argument.

In the revised manuscript, we have restructured the logical flow in the revised Section 2.5, to follow a rigorous “Hypothesis-Verification” framework. We first present the recurrent vertical stripes and their periodicity. Comparing them with the newly added contiguous extension case (revised Fig. 6), we argue that the fragmentation implies a periodic modulation. Based on the ~175-s period observed, we hypothesize that this modulation is driven by ULF waves. We then explicitly state that this hypothesis is supported by the independent ground-based magnetometer observations (revised Fig. 8), which detect coherent wave signatures at the exact same frequency and time, and also by test-particle simulation results shown in Fig. 9. In other words, the link between recurrent vertical stripes and ULF waves is no longer a speculation, but a hypothesis supported by independent data and simulation results.

In the revised manuscript, the relevant paragraphs in Section 2.5 (Lines 589-597) has been rewritten to reflect this logical flow.

Another major lack in this article is that there is not enough support for justifying the choice of *"electric field with an amplitude of several mV/m"* which is 4-6 mV/m for event I and II and *"a duration of a few 15 minutes"* which is very short for effects that meant to last for hours. While this choice is central to the article and affect drastically the solution, the values of the electric field amplitude in the method section is never justified and discussed. This is a major issue. Nowhere a comparison of this value with measurements or/and previous literature is made. This is critic holds for both events. The parameters choice seems to rather be an arbitrary choice made since the beginning of the article.

Thank you for your critical questions. We would first clarify that short-duration electric field perturbations can indeed have long-duration effects in particle distributions, i.e., the zebra stripes. The electric field perturbations act as a trigger that causes inhomogeneous particle distributions along the azimuthal direction. This process does not need to take long. It is the inhomogeneity, together with the energy-dependent drift motion of the particles, that lead to the gradual formation of zebra stripes in the following hours. In other words, the zebra stripes are essentially the drift-echo signatures of the initial, short-lived perturbations in particle distributions.

Also, we note that the electric field amplitude of several mV/m is not very unusual. In fact, such a strong electric field has been proposed to explain the energetic electron dynamics in/below the inner belt (e.g., Selesnick et al., 2019, https://doi.org/10.1029/2019JA026718).

On the other hand, we understand your concern that the values adopted might seem arbitrary without context. However, we note that the chosen electric-field parameters (amplitude of several mV/m, duration ~10 min) are not arbitrarily selected, but inferred from backward-tracing simulation constrained by the observational features. To demonstrate its robustness, we have performed a comprehensive sensitivity analysis on electric field parameters applied in the simulations for Event I (see Supplementary Fig. 4). In this event, the key constraints are that the test electrons outside (both before and after) the stripe must reach the 100-km precipitation boundary when they are traced backward in time, while test electrons within the stripe must have a minimum mirror height above 100 km. The parameter sets satisfying these requirements are indicated by the magenta dots in Supplementary Fig. 4. The results indicate a small feasible domain of electric field parameters. The sensitivity results regarding time shifts indicate the onset time of the perturbed electric field with a precision of minutes. The coupled duration and amplitude ratios relative to the settings used in the main text can vary from (0.7,1.4) to (1.1,0.9), all corresponding to a pulse electric field of several mV/m and duration in minutes.

Moreover, the energy range of the observed vertical stripe also imposes constraints on properties of electric field perturbations. As shown in Supplementary Figs. 4j-4l, a combination of parameters, namely an earlier onset time (time shift of -5 minutes), weaker

amplitude, and longer duration, may also cause a flux enhancement of ~71 keV electrons. However, for higher-energy electrons, due to their faster drift velocity, their flux enhancements within the vertical stripe require acceleration large enough over a shorter time period, necessitating a sufficiently strong electric field. Therefore, the energy range of the observed vertical stripe further narrows the feasible parameter space (note the feasible solutions for two energies do no overlap in the bottom two rows in Supplementary Fig. 4). Thus, the chosen parameter values represent the necessary physical conditions required to reproduce the observations.

Besides the newly addition of the Supplementary Figure 4, we have also revised the Discussion section in Lines 734-750 to quantitatively describe these constraints on electric field properties.

The authors write *"For both events analyzed in this study, the presence of vertical stripes indicates a pulsed electric field with an amplitude of several mV/m and a duration of a few 15 minutes. This supports the generation of zebra stripes by a short-lived electric field pulse [24] rather than a long-duration and small-amplitude electric field [25]."* The authors' method and this choice cannot rule out a "long-duration and small-amplitude electric field" effect which is not considered or studied at all in the article. This is actually strange the authors make such a claim and then bypass justifying or explaining it. The authors could try to perform *"long-duration and small-amplitude electric field"* and discuss what it gives and why this would not be conformed with the observations. The authors did not write if there is a field amplitude threshold above it they can reproduce the observation and below which they do not. There is more demonstration needed to support their choice and ruling out a "long-duration and small-amplitude electric field" effect, and, therefore justify even more the present approach of the authors.

Thank you for the constructive suggestion. As discussed in our response to your previous comment, the addition of Supplementary Fig. 4 (especially the results for 150-keV electrons in the bottom row) provides insights into this issue. More specifically, the requirement that the minimum mirror height must be higher than 100 km for ~150-keV electrons within the "vertical stripe" (Supplementary Fig. 4n) provides a lower amplitude threshold for the electric field perturbations (amplitude ratio ~0.3, corresponding to ~1.8 mV/m). Below this threshold, the electric field would not be strong enough to reduce the electron mirror point to be detected at the satellite orbit height, even if applied for a duration long enough for electron to encounter the electric field throughout the entire drift process from its minimum mirror point within SAA to the satellite position. In other words, this lower limit for electric field amplitude arises because it is simultaneously constrained by the drift duration of high-energy electrons. Besides this lower amplitude threshold, as mentioned above, the observational signatures before, during, and after the vertical stripe appearance provide the combined constrains on the electric field properties, which further limit the feasible solution domain.

We have accordingly revised Lines 754-762 in Discussion section to detail these constrains and discuss its implication for zebra-stripe studies.

For Event II, the magnetic measurements made by the Belk station located at the footprint of the observation (L~2.2) shows proof of the ULF wave modulation and power. This is convincing. What is happening for Event I? This is not discussed at all. Why? I note the location of the footprint of the observation is L~1.9 for event I: is there any other station at this L-shell, which could report ULF fluctuations as well? I am assuming the authors did not find any trace of these waves otherwise they would be mentioned.

The reason why we do not present the ground-based magnetic field observations for Event I is that ULF waves are not required to explain the observations. For Event II in the original manuscript (or Event III in the revised manuscript as we have added another event as the new Event II), the observed electron fluxes exhibit recurrent stripes with a ~175s periodicity. This feature indicates a periodic driver (like ULF waves), which motivates us to examine the existence of ULF waves using the BEL station data. On the other hand, only a single vertical stripe appears in Event I, indicating a single, impulsive driver. In this context, we do not expect to see coherent ULF wave signatures in ground magnetometers for Event I. We do check the observations from ground-based magnetometers near the satellite footprint for Event I (see below; with only 1-min magnetic field data available) though. The results do not show ULF wave signatures, which agrees with our conclusion that the single vertical stripe is generated by an impulsive electric field, distinct from the mechanism in Event III.

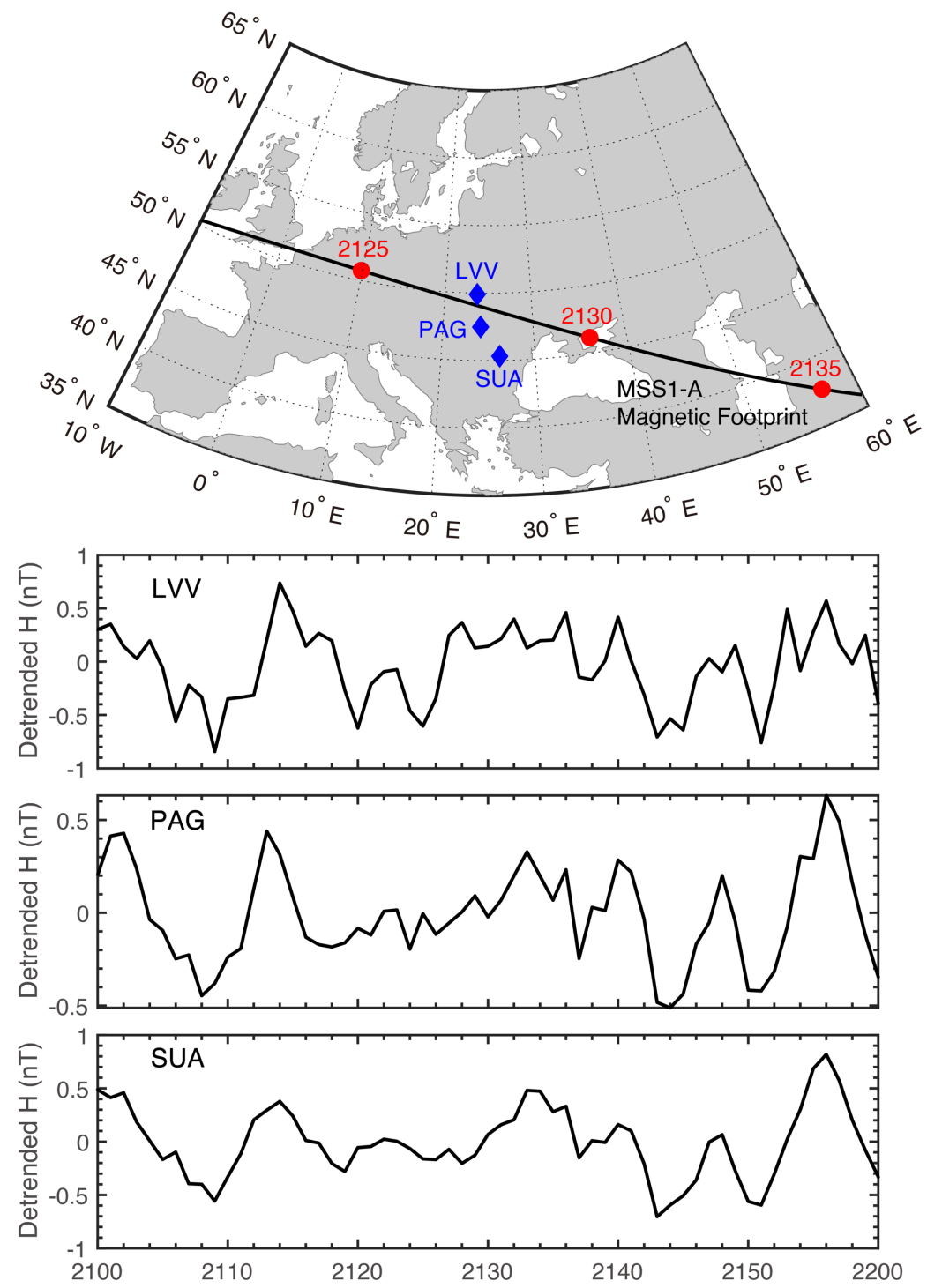

About the Belk ULF measurements: could the authors discuss the amplitude (or power) of the observed ULF waves shown in Figure 7 and relate the measured amplitude (or power) to the strength of the applied electric field fluctuation used in the simulation?

We have indeed analyzed the amplitude of the ULF waves observed at the BEL station. The

peak-to-peak amplitude of the detrended H-component is approximately 2-3 nT (as shown in Fig. 8b). One may assume an ideal mapping of guided Alfvén waves, which would suggest an equatorial electric field amplitude much smaller than the 15 mV/m amplitude used in our simulation.

However, we argue that the in-situ particle observations provide a more direct and reliable constraint on the equatorial electric field strength than the remote ground-based measurements for this specific event. The justification for using the larger amplitude in the simulation is twofold.

The first one is the constraint from particle observations. The simulation amplitude (15 mV/m) is utilized to reproduce the modulation depth of the observed electron fluxes (i.e., the contrast between the peaks and valleys of the stripes). A significantly weaker electric field would not generate the observed recurrent vertical-stripe features, or the deep flux depletion inside the SAA.

Second, it is well known that ground-based magnetometers can underestimate the wave power, due to several factors such as the ionosphere screening effect. Moreover, if the ULF waves have a significant compressional magnetic component (rather than ideal shear Alfven waves), the signatures would be weak on the ground but strong in the equatorial plane.

Therefore, while the ground station confirms the existence and frequency of the wave, we rely on the MSS1 particle observations to constrain the amplitude. We have revised the Discussion (Lines 779-784) to explicitly state this discrepancy, which could be partly attributed to ionospheric screening or a significant compressional component of the wave.

To conclude, I think this article is interesting on the observation, ideas, concepts and some of the method. There is an interest at focusing on these events and try to understand them and maybe deduce some properties of the transient electric field associated to them. However, the method and demonstration are very limited and there are some unexplained major gaps, which, to me, prohibit the publication in Nature Communications. The format of the article is also quite long with many figures and would have to be adapted to match Nature Communications format, which is secondary to me though. About the science, I feel that this article would be better suited for JGR. But the authors will still face some of the issues I mention above in JGR or any other journals and they should reinforce their method and analysis to better demonstrate their claims.

We sincerely appreciate your recognition on the observations, ideas and concepts presented in this paper. As you can see, we have undertaken serious efforts to address your concerns, and we hope that you would agree that the revised manuscript meets the standards of *Nature Communications*.

More specifically, we have added a new event (September 24, 2024) in the main text to address the concern about “limited demonstration” and “unexplained major gaps”. This event

links the isolated “vertical stripe” to a contiguous eastward expansion of the SAA, proving that the reported phenomenon is indeed a fundamental dynamic of the inner radiation belt rather than a marginal case. We have also added a rigorous sensitivity analysis (Supplementary Fig. 4) to quantitatively demonstrate that our backward-tracing method yields sharp constraints on the electric field, which allows us to propose that such SAA distortions can, in principle, serve as a valuable remote-sensing tool for probing and constraining the otherwise elusive inner-belt electric field dynamics (Lines 798-805 in Discussion).

We have also streamlined the manuscript. It now contains 9 figures and is under 5000 words, strictly adhering to the Nature Communications formatting guidelines. We believe that with these significant additions and rigorous improvements, the revised manuscript now provides a solid demonstration and broad impact required for publication in this journal.

Response to Reviewers

We are grateful to the reviewers for their constructive comments, which provided us the opportunity to further improve our paper. We have carefully considered the comments and accordingly revised the manuscript. Please find below the responses to the comments, in which the reviewers' comments are shown in blue and our replies are in black.

To Reviewer #2

I have perused the authors' responses to my comments and those of the other reviewers, and examined the revised manuscript. The authors have made substantial revisions in response to address those comments and concerns. I have no fundamental concern with the paper as a result.

The paper reports new observations supported by modelling which makes some assumptions but seem reasonable. The significance of the paper is in pointing out that energetic electrons can be found beyond the 'traditional' south Atlantic anomaly region due to the effects of transient electric fields. This has important implications for spacecraft operators. Further, more detailed studies including a range of solar wind conditions will clarify the significance of this finding and the modelling but this paper is the first step.

The methodology is clear and reproducible given suitable observations. The figures are complex but well explained, and the supplemental material is very useful.

Thank you for the positive evaluation and for your efforts in reviewing our work. We are glad to know that our revisions have addressed your previous concerns.

Also, we agree with your insightful perspective regarding the future direction of our research. As you pointed out, this paper serves as an essential first step. Building upon the framework established in this paper, we have started conducting more comprehensive, statistical studies that incorporate a wider range of solar wind and geomagnetic conditions. Thanks again for your guidance and support throughout the review process.

To Reviewer #3

This manuscript presents observations from the MSS1 mission of transient energetic electron flux enhancements outside the traditional South Atlantic Anomaly (SAA) region, interpreted as distortions of the inner belt electron environment driven by large-scale electric field perturbations. The study combines multi-satellite observations with test-particle simulations to reproduce key features such as short-duration "vertical stripe" enhancements and subsequent zebra stripe structures. The revised manuscript has been substantially improved compared to the original submission, particularly through the inclusion of an additional event,

an expanded discussion of potential physical drivers, and a sensitivity analysis of the modelled electric fields. These additions strengthen the overall analysis and demonstrate that the authors have engaged seriously with the previous round of review.

Thank you for stepping in to review our manuscript during the second round. We are encouraged by your recognition of the major improvements we made in our previous revision, including the addition of the new contiguous expansion event, the expanded discussion on physical drivers, and the sensitivity analysis of the modeled electric fields.

However, in my assessment, several central concerns remain only partially resolved. In particular, (i) the conceptual definition and use of the SAA throughout the manuscript, (ii) the degree to which the timing and properties of the electric field perturbations are uniquely constrained by the observations, and (iii) the level of generality and novelty claimed relative to prior work. As a result, while the observations are interesting and suitable for publication elsewhere, the current manuscript does not yet support the level of generality and interpretive strength expected for Nature Communications. I therefore recommend that the manuscript is not suitable for publication in its current form in this journal.

We take your remaining concerns very seriously. In this revision, we have carefully addressed all of them, which we believe has significantly elevated the conceptual rigor and interpretive strength of the manuscript. Please find our detailed point-by-point responses below.

Major Comments

1. Definition and use of the SAA

The manuscript would benefit from a clearer and more consistent definition of the South Atlantic Anomaly (SAA), and in particular what is meant by its "boundary" in the context of the presented observations.

The SAA is introduced as a region defined primarily by a depression in the geomagnetic field strength, i.e., a magnetic-field-driven feature. However, the analysis throughout the manuscript is based on energetic electron flux measurements at satellite altitude, and the central claims concern "distortions" or "extensions" of the SAA inferred from these flux enhancements, which are attributed to electric (rather than magnetic) field perturbations.

While an observational definition of the SAA in terms of particle accessibility (e.g., mirror altitude relative to spacecraft altitude) is physically reasonable, this definition is not explicitly formalized or consistently applied. As a result, there is an ambiguity between a magnetic definition of the SAA and an operational definition based on particle fluxes.

This ambiguity makes it difficult to assess whether the reported features should be interpreted as true "distortions of the SAA" or as transient modifications of the electron flux distribution outside the nominal SAA region.

I recommend that the authors either:

- clearly define and consistently apply an observational definition of the SAA (with appropriate references), or
- moderate the interpretation to avoid implying a direct distortion of the SAA if the results, instead, reflect transient changes in particle fluxes at low altitudes and latitudes.

Thank you for pointing out this conceptual issue. We fully agree that the SAA, in its strict sense, is a geomagnetic feature, and that the transient electric-field perturbations discussed in this study cannot distort the magnetic anomaly itself. What is modified in our observations and simulations is indeed the transient distortions of the energetic electron distributions (i.e., the radiation environments) that are typically confined within this magnetic anomaly at LEO altitudes.

To remove this ambiguity, we have revised the manuscript to clearly distinguish between the magnetic SAA and the SAA radiation region relevant to spacecraft observations. In the Introduction, we now define the SAA radiation region for LEO spacecraft observations as the area where stably-trapped inner-belt electrons become accessible to the spacecraft. In the unperturbed state, its boundary is determined by the intersection between the minimum mirror-altitude surface of stably-trapped electrons and the spacecraft orbital plane, as illustrated in Fig. 1a. This reference boundary is now referred to as the nominal SAA boundary, see the revised text in Lines 65-71 and 148-149.

We have also revised Fig. 1 and its caption to visually clarify this definition and formalized the definition of nominal SAA boundary later in Section 2.1 (the location where DLC expands to include electrons at 90-degree pitch angle, see Lines 177-178). Then, we have reviewed the manuscript to refine our terminology throughout, by avoiding the misleading phrases like "SAA distortion". Instead, we use terms such as "outside the nominal SAA boundary" and "transient distortions of the SAA radiation environments." These revisions are intended to clarify that our analysis concerns transient modifications of energetic electron accessibility and distributions at LEO altitudes, not changes in the underlying geomagnetic anomaly.

We believe that these revisions resolve the ambiguity between the magnetic definition of the SAA and the operational particle-flux definition relevant to spacecraft observations, which also makes the interpretation of the reported features clearer and more rigorous.

2. Timing and uniqueness of the inferred electric field perturbation

A central claim of the manuscript is that the observed vertical-stripe enhancement provides strong constraints on the timing and properties of the driving electric field perturbation.

However, it is not yet clear that the observations uniquely require an electric field occurring immediately prior to the MSS1-A pass. During the interval in which the vertical stripe is

observed, the spacecraft samples a changing trajectory in L-shell, magnetic local time, and pitch-angle space. It is therefore possible that the spacecraft intersects a pre-existing drift-shell/longitude structure, rather than observing the immediate formation of the enhancement.

The manuscript uses the subsequent zebra-stripe observations to constrain the timing of the perturbation, but it is also noted that the inferred onset time carries an uncertainty of order hours. Given this uncertainty, the observations alone do not appear to exclude a scenario in which the enhanced population was transported much earlier than 10 minutes and later sampled along the spacecraft trajectory.

The simulations and sensitivity analysis demonstrate that a narrow range of electric field parameters can reproduce the observed features. However, these constraints are inherently model-dependent, and it is not demonstrated that the proposed solution is unique, or that alternative temporal or spatial configurations could not produce similar signatures.

I recommend that the authors:

- more clearly distinguish between constraints derived directly from observations and those inferred through modelling, and
- explicitly address whether alternative scenarios (e.g., earlier transport followed by spatial sampling) can be ruled out.

Thank you for your insightful comment. We agree that it is important to resolve the spatio-temporal ambiguity, namely, whether the spacecraft observed the immediate response to a recent electric-field perturbation, or instead sampled a pre-existing electron-flux structure generated by an earlier perturbation. We also agree that constraints derived directly from observations should be distinguished from those quantified through modeling. Following your suggestion, we have revised the Discussion section, mainly in Lines 730–757, to address these points explicitly.

We first clarify that the timing constraint is not based solely on the subsequent zebra-stripe observations. Although zebra stripes support the occurrence of electric-field perturbations around the relevant time, their onset-time estimates carry uncertainties on the order of hours. We therefore added an independent, observation-driven argument based on the survival of the observed electrons to the east of the nominal SAA boundary. For these electrons to survive, they must have passed through the lowest-mirror-height region with weakest magnetic field near the SAA center while remaining above the atmospheric precipitation boundary at 100 km. If the electric-field perturbation had occurred much earlier, the associated energy gain and mirror-height reduction would have occurred before the electrons reached this lowest-mirror-height region, driving their mirror altitudes below 100 km and causing atmospheric loss. Equivalently, backward tracing the observed quasi-trapped electrons without a recent perturbation would imply mirror altitudes below 100 km within the SAA, which is physically precluded by atmospheric precipitation.

This survival requirement constrains the perturbation timing: the electric-field perturbation must occur after the electrons pass the lowest-mirror-height region but before reaching the spacecraft. Given the electron drift speeds, this corresponds to a time window of approximately 10 minutes before the MSS1 observation. This timing requirement rules out the possibility that the spacecraft merely crossed a preexisting electron-flux structure generated by a much earlier perturbation.

We also distinguish the observation-driven timing constraints from those quantified through modeling. The observed locations, durations, energy ranges, and morphologies of the transient electron-flux distortions impose physical requirements independent of the model specified. For example, the electric field must provide sufficient energy gain to lower the mirror heights of stably-trapped electrons to the spacecraft altitude. While the exact numerical thresholds may depend on the model formulation, the existence of such a physical threshold is model-independent.

In the revised manuscript, we state explicitly that the exact spatio-temporal structure of the electric field cannot be uniquely determined and that the quantitative limits are model-dependent (see Lines 751-752). Nevertheless, the feasible parameter range identified through the sensitivity analysis is bounded by the observed morphology rather than by numerical simulations alone. Electric-field perturbations that are too weak, too late, or too short-lived cannot lower the mirror heights sufficiently to produce the observed flux enhancements outside the nominal SAA boundary, whereas perturbations that are too strong or too long-lasting tend to produce a contiguous expansion rather than an isolated vertical stripe (Lines 755–759). Therefore, although the specific numerical thresholds depend on the adopted model, the need for lower and upper physical limits on the perturbation is imposed by the observed morphology.

We believe these revisions address your concern by separating the observational timing argument from the model-assisted quantification of electric-field properties, and by explicitly ruling out the alternative scenario of spatial sampling of a structure produced by a much earlier perturbation.

3. Level of novelty and generality

The manuscript presents compelling case studies of transient flux enhancements and their possible interpretation in terms of electric field perturbations. However, the level of novelty relative to prior observations of flux enhancements outside the SAA remains somewhat interpretational, rather than observational.

Similarly, the manuscript introduces the idea that these features may provide a “diagnostic tool” for constraining electric field properties. While this is an interesting prospect, the current analysis is based on a small number of events and a specific modelling framework. The extent to which this approach can be generalized is not yet demonstrated.

As such, several of the broader claims regarding novelty, generality, and diagnostic capability appear stronger than is supported by the current evidence.

I suggest that the authors moderate these claims and more clearly position the work as a promising but still developing interpretation, rather than a broadly established diagnostic framework.

Thank you for your constructive evaluation. Regarding the observational novelty, we acknowledge that the presence of electron flux enhancements outside the SAA is not unprecedented. As we discussed in the manuscript and during the previous round of review, previous studies have documented flux enhancements driven by other mechanisms, such as VLF transmitter wave scattering (forming "wisps") or inward radial transport (forming Forbidden Energetic Electrons at L<1.2).

However, we respectfully clarify that our novelty is not solely interpretational, but also observational. The transient distortions of the SAA radiation environments reported here, manifesting as vertical stripes or contiguous expansions, are observationally distinct from these previously-reported phenomena. Therefore, our primary novelty lies both in reporting this new observational manifestation of inner-belt dynamics and in providing a physical interpretation for these features. To explicitly acknowledge prior works while precisely positioning our novelty without exceeding the word limits, we have inserted a concise clarifying statement at the beginning of the Discussion section (Lines 720-722).

Regarding the "diagnostic tool" claim, we agree that, based on a small number of events and the specific modeling approach, such an application should be regarded as a promising possibility rather than a broadly established framework. Although our manuscript did not intend to claim a generalized diagnostic framework, we have further refined the relevant statements to make this limitation explicit and to avoid possible overinterpretation. In particular, we added a sentence in the final paragraph (Lines 812–815) acknowledging that further studies are required to establish a broadly applicable diagnostic framework. We also replaced stronger terms such as "validate", "verify" and "demonstrate" with more cautious wording such as "evaluate", "examine" or "suggest" where appropriate.

In summary, this manuscript presents interesting and well-executed observations, and the authors have made meaningful improvements in response to prior reviews. However, key aspects of the interpretation remain insufficiently constrained, and several central claims are stronger than can currently be supported.

We believe that the revised manuscript has carefully addressed your concerns, by (1) providing an operational definition of the nominal SAA boundary based on particle accessibility, (2) distinguishing the observation-driven constraints from those further quantified through modeling, and (3) moderating our statements regarding the diagnostic framework and interpretational novelty of our work. Thank you once again for your time and insights.

Remarks on code availability:

The code is easily accessible via the links in the manuscript, as expected, as it is virtually unchanged from the previous review round. This includes an informative README file.

As previous, upon examination of the code functions, I can see good commenting of the code, and instructions on how to reproduce the analysis. This code is well archived and is a positive resource for the community, should they intend to reproduce the results of the paper.

Thank you for taking the time to review our code repository again and for your highly positive remarks. We deeply appreciate your recognition of our efforts.

To Reviewer #4

I reviewed the revised version of the article entitled "Transient Distortions of the South Atlantic Anomaly Radiation Environments Driven by Electric Fields" written by Ze-Fan Yin and co-authors.

I recall the authors use the measurements from both satellites of the Macau Science Satellite mission, MSS1 and MSS2, in orbit at ~450-500 km, with which they report what they called "transient distortions of the South Atlantic Anomaly (SAA) radiation environment". This "transient distortions" can be seen as additional patch of electron radiation in the vicinity of the SAA region, where radiation is the highest due to the lower values of the Earth magnetic field. One impacting conclusion of the authors is that "radiation threats from inner belt particles to LEO spacecraft are not confined to the traditional SAA region but can extend dynamically beyond it.". The authors' thesis is the explanation of these additional radiation regions by large-scale westward electric field perturbations. The latter cause electron acceleration and change the mirror point of the electrons, which remain more trapped, forming additional radiation patches. The authors add that these electric field perturbations are sometimes influenced and modulated by ultra-low-frequency waves (cf. their Event 3).

In my last review, I had indicated unexplained major gaps, which were, to me, prohibiting the publication. It turns out the authors have addressed all of them in their revised version, in a very thorough and convincing manner. I note that some of my comments were also raised by the other reviewers. Reading all rebuttals, I can see the authors have answered all comments in a very precise manner. I also appreciated how detailed is the reasoning brought in the rebuttal associated to my review.

Let please me come back specifically and briefly on their corrections:

- Lack a knowledge of transient electric fields in the ionosphere: the authors now provide a better description of their modeling as well as a sensitivity study (in Supp Mat) of the model response in order to bracket their answer. They specifically write the parameter choice is not arbitrary but guided by the observations.

- Lack of measurements during 7 hours in between the two figures shown: the authors followed my advice and now give the intermediate snapshots (in the Supp Mat Fig 1 and 2) so that readers cannot doubt what is happening in between the two observations. In particular, we now see (from both MSS1 A and B) that the enhanced fluxes are concentrated within the SAA region, i.e. in a normal manner.
- Precision of the altitude orbit and its possible importance: the authors answer in a convincing manner that the difference in altitude do not impact their conclusions.
- Numerical method not enough supported: I thought the numerical method was not enough supported. I think I better understand now what they do thanks to the various clarification, including a newly added reference (Marchand, 2010). It is pretty straight forward. They clarified how Liouville's theorem is used too. The code is well available and clearly coded if needed.
- Incorrect magnetic field: I had spotted that the authors were not using a correct magnetic field model: an "eccentric dipole" while it is important to also tilt the dipole to get an accurate approximation of the full model provided by the IGRF. It turns out the authors explain in their rebuttal that their "eccentric dipole" is also actually tilted and they prove it with a few plots and a discussion of quality. That issue is thus now resolved. Except that the text is not corrected. This is one of the corrections I ask below.
- Writing: there were speculations that have been rephrased and some expressions undermining the results. This has been corrected.
- "long-duration and small-amplitude electric field" effect are ruled out in a more balanced manner, based on the sole observed events, and not generally. With this limitation, I now agree.
- Lack of ULF ground measurement for Event I: this is now well stated that this event did not have ULF wave acting while Event 3 is chosen because it does.
- About the ground-based ULF measurements (in Belk): the authors now discuss the amplitude of the observed ULF waves and relate it well to the strength of the applied electric field fluctuation used in the simulation.

In addition to the previous results, the authors have added two major points:
- They added Event 2 and its analysis (the previous Event 2 became Event 3). This is very interesting because the new Event 2 shows a continuous extension of the SAA and again the authors apply their theory to try to infer the electric field fluctuations associated to that new flux. Event 2 directly addresses my concern about the nature of these distortions, shown previously only as disjoint flux peaks.
- They better put forward another impactful application of their work: a statistical analysis of that type of flux event could allow to infer electric field fluctuations and their occurrence rate, that are hard to measure and quantify. I agree.

To conclude, I think this article is interesting on the observation, ideas, concepts and the method. The authors have made a tremendous solid and rigorous work to support their claims, in the rebuttals and in the main text, in order to remove all potential critics. That makes me conclude that it is possible to consider the publication of this study in Nature Communications, if the other reviewers did not find critical objections. The format of the article remains quite

long with (5000 words with 9 figures). It has now an inflated supplemental material, which was necessary to bring all the additional information. I think the 3 events that are chosen are required because they all show a distinct feature so that one cannot be removed. So there maybe formatting issue your publication team will need to deal with. Not my concern. Below follow the last minor corrections for the authors I found.

We are grateful for your positive evaluation of our manuscript and for taking the time to write such a comprehensive and accurate summary of our revisions. We are so happy to hear that our revisions have addressed all your previous concerns in a thorough and convincing manner.

Regarding the manuscript's format and length, we agree with your assessment that all three events are essential for demonstrating the distinct features of the phenomenon and cannot be removed. We are also pleased to share that the handling editor has kindly reviewed our format and confirmed that our current main file aligns with the length and formatting limits of the journal. Please find below our point-by-point responses to your minor suggestions.

Minor corrections for the authors

Line 264 : "Seven hours after the appearance of the vertical stripe, MSS1-B traverses" Again, the readers do not know why the authors pick 7 hour after, why not 5 mins or 24h. they can write something, either before this sentence or after, to introduce why they pick that time (likely inferred from simulations made in the next section).

Thank you for pointing this out. As you pointed out, it usually takes several hours for the energy-dependent drift motions to fully develop into clear zebra-stripe patterns. We have modified the relevant sentence (in Lines 260-262) to explain that zebra-stripe patterns are fully developed seven hours later.

Line 345 reads "an eccentric dipole model that provides a good approximation". The authors have to write they use an "eccentric tilted dipole model" so that it is clear. This correction remains to be done Line 345, 850, 864, 870, 875 in the Methods section. If the authors want to save words, they can define "ETD" as "eccentric tilted dipole". I note that they newly added in the Methods section the value of the tilt angle which does clarify the modeling, but too late at the end of the article. An "eccentric dipole" may or may not be tilted. Usually it is not tilted, because if it was, one would write "eccentric tilted dipole"…. Please correct.

We fully agree. We have corrected the terminology to "eccentric tilted dipole" in the main text (Lines 346 and 349), and in the Methods section adopted "ETD" as the abbreviation of "eccentric tilted dipole."

Line 533 : figure 6 caption: "except that the pitch-angle distributions are not shown". They can write in which figure (->Fig6d).

We have updated the caption of Figure 6 (and similarly for Figure 7) to explicitly indicate

which panel from the referenced format was not included. Thank you once again for your insights throughout the revision process.

Review for Nature Communications
Reference of the article: NCOMMS-25-75431
Title: Transient Distortions of the South Atlantic Anomaly Radiation Environments Driven by Electric Fields
Authors: Ze-Fan Yin and co-authors

Dear Editor,

I reviewed the article entitled "Transient Distortions of the South Atlantic Anomaly Radiation Environments Driven by Electric Fields" written by Ze-Fan Yin and co-authors.

The authors use the measurements from both satellites of the Macau Science Satellite mission, MSS1 and MSS2, which orbit at ~450-500 km, with which they report what they called "*transient distortions of the South Atlantic Anomaly (SAA) radiation environment*". This "*transient distortions*" can be seen as additional patch of electron radiation in the vicinity of the SAA region, where radiation is the highest due to the lower values of the Earth magnetic field. One impacting conclusion of the authors is that "*radiation threats from inner belt particles to LEO spacecraft are not confined to the traditional SAA region but can extend dynamically beyond it*.".

The discovery the author would like to publish in Nature Communications is the observation and explanation of these additional radiation regions by large-scale westward electric field perturbations. The latter cause electron acceleration and change the mirror point of the electrons, which remain more trapped, forming additional radiation patches. The authors add that these electric field perturbations are influenced and modulated by ultra-low-frequency waves. There is a difficulty which the authors note in their introduction which is the current lack of knowledge of the amplitude of transient electric fields in the ionosphere and near-Earth space. We will see below that the authors do not succeed at well justifying their choice for the values and shape of the transient electric fields, which undermines greatly their article and its impact. The detection of the vertical high flux region in the vicinity of the SAA, associated by the authors to some electric field, is made by MMS1 while the second satellite, MSS2, helps to identify the longer-term claimed effect on the SAA electron flux, which should produce some structuration of the radiation flux (called zebra stripes), indeed observed. I find several major lacks or questions which should prevent publication in Nature Communications.

From the map of figure 1a, the radiation patch is indeed separated from the main radiation region. The patch (i.e the vertical stripe) is located east of the tip of Africa at longitude close to Madagascar (see below more comments on the location). This is interesting. However, I note, first the zebra stripe structures were also measured in the first place by MSS1 and, second, MSS2 has a different trajectory travelling within a different region of the SAA. Figure 3 shows the MSS2 data in the region 7h later, while this spacecraft has a 94 mins orbit, meaning 4 orbits separate the

observations and we do not know what the spacecraft did measure in between the two measurements. Besides, MMS1 and MSS2 altitude orbits are different by 50 km, which is crucial here according to the claim made in Figure 1. Altitude is not mentioned in the text or plotted in the figure. The role it can have is yet unclear. The authors discuss (cf. Figure 1 and associated text) an effect that is associated to variations within "*a few of hundreds of kilometers*", which is in the range of the 50 km of variation of the orbit of MMS2. The "*few hundreds of kilometers*" is ~200 km reading Figure 1a and 100 km according to Figure 5c, 5i, 5o. This is in the range of the s/c altitude variation. The authors discuss that electrons cannot anymore "*access the spacecraft at lower altitudes*". Maybe it is just the s/c since the s/c altitude has changed. This is not discussed or accounted for.

The simulated process by which the spectrograms change from Figure 4a-b to Figure 4c-d is not described in the article. We only read "*Figures 4c-4d show the simulation results at MSS1-B locations several hours afterwards, which reproduce the observations of zebra stripes for locally mirroring electrons and confined pitch-angle distributions outside the local DLC (compared to observations in Fig. 3*)." This is not an analysis. This is a statement without justification. Between both states, there are 7 hours which are spent and we do not see or read about what is happening during that time (from both observations and simulations point of views). This is intriguing since the claimed process of electric field increase is just 9 minutes long. Also the flux measurements of MSS1 et MSS2 are not made in the same region in (Lat,Long) while I believe the simulation from Figure 4a-b to Figure 4c-d is likely made. Not only both s/c do not have the same altitude but also not the same trajectory, with the second not passing through the region of the vertical stripes. This should be more discussed and justified. Why not looking at MSS1 7 or more hours later, then, in the same place? It is unclear what MSS-1 measures over a few orbits: are the vertical stripes gone in the next orbit? The authors should provide snapshots of the electron flux for each pass in the Supp Mat. Same comment holds for MSS2.

About the method, the authors employ a "*backward-tracing simulations of test electrons launched from the spacecraft location to reproduce the observational characteristics*", which is central to the demonstration. The main equations are given in the Methods section. I find the method quite old. It is mentioned that Liouville's equation are also solved, no detail given. This whole numerical code has no references, no test cases, and has not been used for other publication. This is not fine to me for Nature Communications. The numerical method is not enough supported.

In their method, the authors do not use a full and accurate magnetic field model such as the IGRF model, which they state and justify. They use instead (for simplicity) an eccentric dipole. They do not tilt the magnetic field model either for unknown reason. Because of their approximation, they write that their results *"should be interpreted as robust for large-scale signatures, but not as precise representations of fine-scale field structure".* I do not think that it is true. How can they assess this is true? They are dealing with fine-scale effects of 100 km and authorize themselves rough approximations, which matter greatly for the definition of the SAA. The SAA is defined by lower magnetic field. If one looks at the low magnetic field region in the SAA at 100 km of altitude, the edge of the SAA as modeled by an eccentric tilted dipole is roughly at the tip of Africa (roughly corresponding with the region plotted as the trapped region in Figure 2 or 3).

If one does now look at the IGRF model, the edge of the SAA reaches now the longitude of Madagascar, exactly where the authors find their additional patch. There is an abrupt change in magnetic field in the eastern tip of Africa, not captured by an eccentric tilted dipole. The whole south-eastern tip of Africa falls in the main SAA with the IGRF and not with an eccentric tilted dipole. This means the vertical stripe could simply still be in the SAA, contrarily to what is claimed by the authors. The detail effect of the magnetic field matters a lot in this region. Again, the method here seems insufficient and possibility misleads the authors in their conclusions.

Throughout the article, the authors are careful with not overstating their results and balancing them, which I appreciated reading. However, there is a loss of impact, which is actually supported by an incomplete method. They write "*the recurrent stripes are <u>likely caused</u> by periodic electric field perturbations …..*". The point of the whole article would be to demonstrate that effect, with a demonstration that is acknowledged as not done and indeed not well supported. The authors also write "*Moreover, since the three vertical stripes exhibit a periodicity of ∼ 175 seconds (falling within the ULF range), it is natural <u>to speculate</u> that the electric field perturbations are carried, at least partially, by ULF waves*.". I think nobody wants *speculations* in Nature Communications, in particular regarding a main claim.

Another major lack in this article is that there is not enough support for justifying the choice of "*electric field with an amplitude of several mV/m"* which is 4-6 mV/m for event I and II and *"a duration of a few 15 minutes*" which is very short for effects that meant to last for hours. While this choice is central to the article and affect drastically the solution, the values of the electric field amplitude in the method section is never justified and discussed. This is a major issue. Nowhere a comparison of this value with measurements or/and previous literature is made. This is critic holds for both events. The parameters choice seems to rather be an arbitrary choice made since the beginning of the article.

The authors write "*For both events analyzed in this study, the presence of vertical stripes indicates a pulsed electric field with an amplitude of several mV/m and a duration of a few 15 minutes. This supports the generation of zebra stripes by a short-lived electric field pulse [24] rather than a long-duration and small-amplitude electric field [25]."* The authors' method and this choice cannot rule out a "*long-duration and small-amplitude electric field*" effect which is not considered or studied at all in the article. This is actually strange the authors make such a claim and then bypass justifying or explaining it. The authors could try to perform "*long-duration and small-amplitude electric field*" and discuss what it gives and why this would not be conformed with the observations. The authors did not write if there is a field amplitude threshold above it they can reproduce the observation and below which they do not. There is more demonstration needed to support their choice and ruling out a "*long-duration and small-amplitude electric field*" effect, and, therefore justify even more the present approach of the authors.

For Event II, the magnetic measurements made by the Belk station located at the footprint of the observation (L~2.2) shows proof of the ULF wave modulation and power. This is convincing. What is happening for Event I? This is not discussed at all. Why? I note the location of the footprint of the observation is L~1.9 for event I: is there any other station at this L-shell, which could report

ULF fluctuations as well? I am assuming the authors did not find any trace of these waves otherwise they would be mentioned.

About the Belk ULF measurements: could the authors discuss the amplitude (or power) of the observed ULF waves shown in Figure 7 and relate the measured amplitude (or power) to the strength of the applied electric field fluctuation used in the simulation?

To conclude, I think this article is interesting on the observation, ideas, concepts and some of the method. There is an interest at focusing on these events and try to understand them and maybe deduce some properties of the transient electric field associated to them. However, the method and demonstration are very limited and there are some unexplained major gaps, which, to me, prohibit the publication in Nature Communications. The format of the article is also quite long with many figures and would have to be adapted to match Nature Communications format, which is secondary to me though. About the science, I feel that this article would be better suited for JGR. But the authors will still face some of the issues I mention above in JGR or any other journals and they should reinforce their method and analysis to better demonstrate their claims.

Review for Nature Communications
Reference of the article: NCOMMS-25-75431A
Title: Transient Distortions of the South Atlantic Anomaly Radiation Environments Driven by Electric Fields
Authors: Ze-Fan Yin and co-authors

Dear Editor,

I reviewed the revised version of the article entitled "Transient Distortions of the South Atlantic Anomaly Radiation Environments Driven by Electric Fields" written by Ze-Fan Yin and co-authors.

I recall the authors use the measurements from both satellites of the Macau Science Satellite mission, MSS1 and MSS2, in orbit at ~450-500 km, with which they report what they called "*transient distortions of the South Atlantic Anomaly (SAA) radiation environment*". This "*transient distortions*" can be seen as additional patch of electron radiation in the vicinity of the SAA region, where radiation is the highest due to the lower values of the Earth magnetic field. One impacting conclusion of the authors is that "*radiation threats from inner belt particles to LEO spacecraft are not confined to the traditional SAA region but can extend dynamically beyond it*.". The authors' thesis is the explanation of these additional radiation regions by large-scale westward electric field perturbations. The latter cause electron acceleration and change the mirror point of the electrons, which remain more trapped, forming additional radiation patches. The authors add that these electric field perturbations are sometimes influenced and modulated by ultra-low-frequency waves (cf. their Event 3).

In my last review, I had indicated unexplained major gaps, which were, to me, prohibiting the publication. It turns out the authors have addressed all of them in their revised version, in a very thorough and convincing manner. I note that some of my comments were also raised by the other reviewers. Reading all rebuttals, I can see the authors have answered all comments in a very precise manner. I also appreciated how detailed is the reasoning brought in the rebuttal associated to my review.
Let please me come back specifically and briefly on their corrections:

- Lack a knowledge of transient electric fields in the ionosphere: the authors now provide a better description of their modeling as well as a sensitivity study (in Supp Mat) of the model response in order to bracket their answer. They specifically write the parameter choice is not arbitrary but guided by the observations.
- Lack of measurements during 7 hours in between the two figures shown: the authors followed my advice and now give the intermediate snapshots (in the Supp Mat Fig 1 and 2) so that readers cannot doubt what is happening in between the two observations. In particular, we now see (from both MSS1 A and B) that the enhanced fluxes are concentrated within the SAA region, i.e. in a normal manner.
- Precision of the altitude orbit and its possible importance: the authors answer in a convincing manner that the difference in altitude do not impact their conclusions.

- Numerical method not enough supported: I thought the numerical method was not enough supported. I think I better understand now what they do thanks to the various clarification, including a newly added reference (Marchand, 2010). It is pretty straight forward. They clarified how Liouville's theorem is used too. The code is well available and clearly coded if needed.
- Incorrect magnetic field: I had spotted that the authors were not using a correct magnetic field model: an "eccentric dipole" while it is important to also tilt the dipole to get an accurate approximation of the full model provided by the IGRF. It turns out the authors explain in their rebuttal that their "eccentric dipole" is also actually tilted and they prove it with a few plots and a discussion of quality. That issue is thus now resolved. Except that the text is not corrected. This is one of the corrections I ask below.
- Writing: there were speculations that have been rephrased and some expressions undermining the results. This has been corrected.
- "*long-duration and small-amplitude electric field*" effect are ruled out in a more balanced manner, based on the sole observed events, and not generally. With this limitation, I now agree.
- Lack of ULF ground measurement for Event I: this is now well stated that this event did not have ULF wave acting while Event 3 is chosen because it does.
- About the ground-based ULF measurements (in Belk): the authors now discuss the amplitude of the observed ULF waves and relate it well to the strength of the applied electric field fluctuation used in the simulation.

In addition to the previous results, the authors have added two major points:

- They added Event 2 and its analysis (the previous Event 2 became Event 3). This is very interesting because the new Event 2 shows a continuous extension of the SAA and again the authors apply their theory to try to infer the electric field fluctuations associated to that new flux. Event 2 directly addresses my concern about the nature of these distortions, shown previously only as disjoint flux peaks.
- They better put forward another impactful application of their work: a statistical analysis of that type of flux event could allow to infer electric field fluctuations and their occurrence rate, that are hard to measure and quantify. I agree.

To conclude, I think this article is interesting on the observation, ideas, concepts and the method. The authors have made a tremendous solid and rigorous work to support their claims, in the rebuttals and in the main text, in order to remove all potential critics. That makes me conclude that it is possible to consider the publication of this study in Nature Communications, if the other reviewers did not find critical objections. The format of the article remains quite long with (5000 words with 9 figures). It has now an inflated supplemental material, which was necessary to bring all the additional information. I think the 3 events that are chosen are required because they all show a distinct feature so that one cannot be removed. So there maybe formatting issue your publication team will need to deal with. Not my concern. Below follow the last minor corrections for the authors I found.

Minor corrections for the authors

Line 264 : « Seven hours after the appearance of the vertical stripe, MSS1-B traverses “ Again, the readers do not know why the authors pick 7 hour after, why not 5 mins or 24h. they can write something, either before this sentence or after, to introduce why they pick that time (likely inferred from simulations made in the next section).

Line 345 reads “an eccentric dipole model that provides a good approximation”. The authors have to write they use an “eccentric tilted dipole model” so that it is clear. This correction remains to be done Line 345, 850, 864, 870, 875 in the Methods section. If the authors want to save words, they can define “ETD” as “eccentric tilted dipole”. I note that they newly added in the Methods section the value of the tilt angle which does clarify the modeling, but too late at the end of the article. An “eccentric dipole” may or may not be tilted. Usually it is not tilted, because if it was, one would write “eccentric tilted dipole”…. Please correct.

Line 533 : figure 6 caption : « except that the pitch-angle distributions are not shown”. They can write in which figure (→Fig6d).
